# *Djebelemur*, a Tiny Pre-Tooth-Combed Primate from the Eocene of Tunisia: A Glimpse into the Origin of Crown Strepsirhines

**DOI:** 10.1371/journal.pone.0080778

**Published:** 2013-12-04

**Authors:** Laurent Marivaux, Anusha Ramdarshan, El Mabrouk Essid, Wissem Marzougui, Hayet Khayati Ammar, Renaud Lebrun, Bernard Marandat, Gilles Merzeraud, Rodolphe Tabuce, Monique Vianey-Liaud

**Affiliations:** 1 Laboratoire de Paléontologie, Institut des Sciences de l’Évolution de Montpellier (ISE-M, UMR-CNRS 5554), Université Montpellier 2, Montpellier, France; 2 Section of Vertebrate Paleontology, Carnegie Museum of Natural History, Pittsburgh, Pennsylvania, United States of America; 3 Office National des Mines (ONM), Tunis, Tunisia; 4 Géosciences Montpellier (UMR-CNRS 5243), Université Montpellier 2, Montpellier, France; Team ‘Evo-Devo of Vertebrate Dentition’, France

## Abstract

**Background:**

Molecular clock estimates of crown strepsirhine origins generally advocate an ancient antiquity for Malagasy lemuriforms and Afro-Asian lorisiforms, near the onset of the Tertiary but most often extending back to the Late Cretaceous. Despite their inferred early origin, the subsequent evolutionary histories of both groups (except for the Malagasy aye-aye lineage) exhibit a vacuum of lineage diversification during most part of the Eocene, followed by a relative acceleration in diversification from the late Middle Eocene. This early evolutionary stasis was tentatively explained by the possibility of unrecorded lineage extinctions during the early Tertiary. However, this prevailing molecular view regarding the ancient origin and early diversification of crown strepsirhines must be viewed with skepticism due to the new but still scarce paleontological evidence gathered in recent years.

**Methodological/Principal Findings:**

Here, we describe new fossils attributable to *Djebelemur martinezi*, a≈50 Ma primate from Tunisia (Djebel Chambi). This taxon was originally interpreted as a cercamoniine adapiform based on limited information from its lower dentition. The new fossils provide anatomical evidence demonstrating that *Djebelemur* was not an adapiform but clearly a distant relative of lemurs, lorises and galagos. Cranial, dental and postcranial remains indicate that this diminutive primate was likely nocturnal, predatory (primarily insectivorous), and engaged in a form of generalized arboreal quadrupedalism with frequent horizontal leaping. *Djebelemur* did not have an anterior lower dentition as specialized as that characterizing most crown strepsirhines (i.e., tooth-comb), but it clearly exhibited a transformed antemolar pattern representing an early stage of a crown strepsirhine-like adaptation (“pre-tooth-comb”).

**Conclusions/Significance:**

These new fossil data suggest that the differentiation of the tooth-comb must postdate the djebelemurid divergence, a view which hence constrains the timing of crown strepsirhine origins to the Middle Eocene, and then precludes the existence of unrecorded lineage extinctions of tooth-combed primates during the earliest Tertiary.

## Introduction

Lorisiformes (Afro-Asian lorises and African galagos) and Lemuriformes (Malagasy lemurs) make up the Strepsirhini *sensu stricto* (S.*s.s.*), the living “tooth-combed” primates. The extinct Adapiformes, which were non tooth-combed, are commonly viewed as the “lemur-like” primates of the Eocene epoch, and represent the closest out-group of crown Strepsirhini (or Strepsirhini *sensu lato* [S.*s.l.*]) [1]–[13]. Reconstructing the origin and early evolutionary history of strepsirhines is a current focus of paleoprimatology. Although the fossil record of early lorisiforms has rapidly improved over the last decade, notably for the Paleogene of Africa [8], [14], [15], lemuriform fossil evidence has proven to be elusive, with the exception of Malagasy subfossils [16], [17]. In addition to new fossils, genetic data on extant Euprimate species have also substantially increased. Together, both have enabled researchers to trace the geographic origin of crown strepsirhines back to Africa, leading to the suggestion that lemuriforms colonized Madagascar by crossing the Mozambique Channel [8], [18]–[24]. In a notable implication, this recently expanded lorisiform fossil record [8], [14] has provided substantial paleontological credence to the hypothesis of a late Middle or Late Eocene African divergence between lorises (Lorisidae) and galagos (Galagidae), as estimated by DNA sequence data [19], [20], [25]–[29]. Likewise, molecular clock estimates have also shown that the main radiation of lemuriforms at the origin of almost all extant Malagasy lineages (Indriidae, Lepilemuridae, Cheirogaleidae, and Lemuridae, except Daubentoniidae [*Daubentonia*]) occurred in a time window coeval to that of the African lorisiform radiation [19], [20], [23], [28], [29]. However, the timing of the Lorisiformes-Lemuriformes divergence is still a matter of debate. Molecular estimates of crown strepsirhine origins generally advocate an early origin for both groups near the onset of the Tertiary [25], [27], [29], [30], but most often extending back to the Late Cretaceous [19], [20], [23], [26], [28], [31]–[33] – estimates that far precede the appearance of Euprimates in the global fossil record [34]–[37]. Such an inferred ancient antiquity of crown strepsirhines would imply that the differentiation of the tooth-comb occurred before or during the Paleocene (or even the earliest Eocene), and that the ancestral lemuriform colonized Madagascar by the earliest Tertiary [19], [20], [23], [28], [32]. However, this prevailing molecular view regarding the ancient origin and early diversification of crown strepsirhines must be viewed with skepticism due to the new paleontological evidence gathered in recent years, and the re-evaluation of some previously described but poorly known taxa from the Paleogene of Africa.

The Eocene Djebelemuridae (*Djebelemur martinezi*, “*Anchomomys*” *milleri*), Azibiidae (*Azibius trerki*, *Algeripithecus minutus*), and Plesiopithecidae (*Plesiopithecus teras*), although initially not recognized as such, now appear to be closely related to crown Strepsirhini to the exclusion of Adapiformes or any other primate groups (i.e., anthropoids) [8], [11], [14], [21], [35], [36], [38]–[40]. The presence of “pre-tooth-combed” primates or “advanced” stem strepsirhines as early as the late Early Eocene in Africa (*Djebelemur*, *Azibius* and *Algeripithecus*) better constrains the timing of crown strepsirhine origins (i.e., Lorisiformes and Lemuriformes [S.*s.s.*]) to the Middle Eocene, rather than much earlier as estimated on molecular ground. These African stem taxa occupy a key position in strepsirhine phylogeny. Given the fragmentary nature of their fossil record, additional evidence is needed to further our understanding of this paleontological scenario.

**Figure 1:**
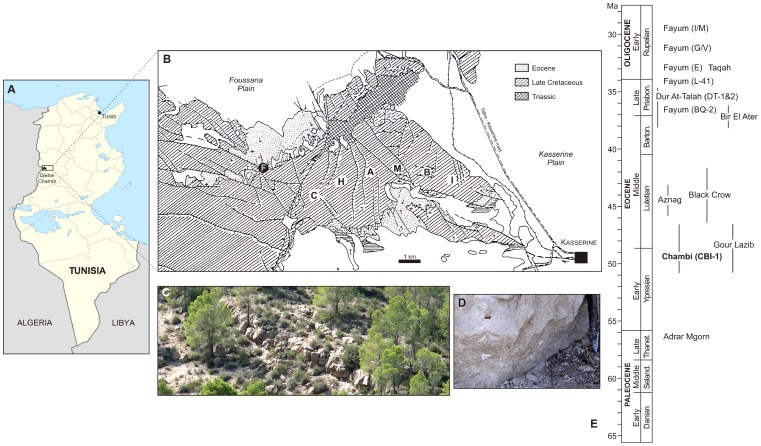
Location map of the primate-bearing Chambi locus 1 locality (CBI-1) in Tunisia. **A**, map of Tunisia locating the Natural Park of Djebel Chambi, a mountain situated in the western part of Central Tunisia (Kasserine region); **B**, geological map of Djebel Chambi (modified after Hartenberger et al. [42]) showing the position of the fossiliferous CBI-1 locality in the Eocene deposits (white “F” in a black filled circle); **C**, landscape photograph of the lacustrine limestone bed (freshwater deposits), which has yielded CBI-1; **D**, photograph of the indurated limestone bed of CBI-1 showing a fossiliferous spot (Pictures by Laurent Marivaux); **E**, temporal distribution of primate-bearing localities from the Paleogene of Afro-Arabia (modified after Seiffert [38] and Coster et al. [64]).

Here, we describe new fossils attributable to *Djebelemur martinezi*, a≈50 Ma primate from the late Early to early Middle Eocene deposits of Djebel Chambi, Tunisia (Fig. 1) [41]–[43]. This taxon was originally interpreted as a cercamoniine adapid primate (i.e., Adapiformes) based on limited fossil evidence primarily from its lower dentition (a mandible preserving p3-m3 [41]). Subsequently, *Djebelemur* was interpreted as a possible basal anthropoid [44] or as a cercamoniine lying “near the prosimian-anthropoidean transition” [45]. In addition to the recently described strepsirhine petrosal bones from Chambi (Fig. 1), tentatively attributed to *Djebelemur* [40], new fossils of this species from the same locality, including a facial fragment (maxilla) preserving P3-M3, a lower jaw fragment preserving m1-2 and p3, a couple of isolated teeth, and an isolated talus provide additional morphological evidence for demonstrating the “advanced” stem strepsirhine status of *Djebelemur*, which is clearly more closely related to tooth-combed primates (crown Strepsirhini; S.*s.s.*) than any adapiforms (S.*s.l.*) [8], [9], [14], [35], [36]. In this paper, we describe and discuss these new fossils with a special emphasis on phylogenetic and evolutionary implications. Some critical aspects of the paleobiology (locomotion, activity pattern, diet) of this ancient primate from North Africa are also discussed, as they provide new insights into the emergence of the tooth-combed primates.

## Materials and Methods

### Fossil Recovery, Extraction, Repository, and Digitalization

#### Fossil recovery and extraction

Fossils were recovered in the framework of our paleontological program in the early Tertiary of North Africa. Since 2008, we have focused some of our field researches in Tunisia, notably on the geological outcrops exposed in the Natural Park of Djebel Chambi (Fig. 1). We have returned to the original primate-bearing locality (Chambi locus 1: CBI-1) and extracted several hundred kilograms of sediments (lacustrine limestone; Fig. 1C). The fossil material presented in this paper was obtained after several rounds of acid processing and screen-washings of the indurated calcareous matrix (Fig. 1D). These repetitive operations have led to the recovery of several dental, cranial and postcranial remains of mammals including marsupials, rodents, bats, eulipotyphlans, creodonts, elephant-shrews, hyracoids, and primates. According to the Tunisian legislation, all necessary permits were obtained for the described field studies from the relevant authorities, namely the “*Office National des Mines*” of Tunis and the “*Office National des Forêts*” of Kassérine.

#### Fossil repository

All fossils referenced (CBI-1-xx; holotype [41] and hypodigm), described and figured in this paper are housed in the paleontological collections of the museum of the “*Office National des Mines*” of Tunis, Tunisia.

#### High-resolution micro-CT scan

**Figure 2:**
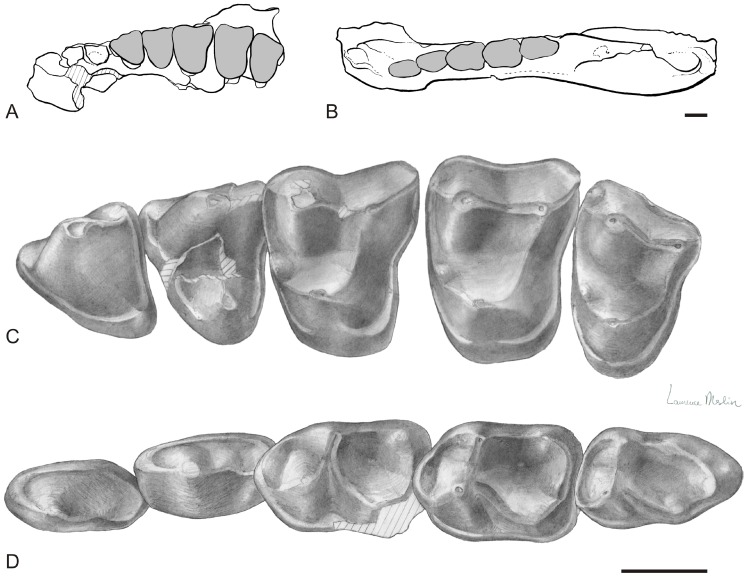
Upper and lower jaws of *Djebelemur martinezi* from the Djebel Chambi CBI-1 locality. **A**, outline of the CBI-1-565 maxilla in occlusal view; **B**, outline of the CBI-133 mandible (holotype) in occlusal; **C**, drawings of the upper toothrow (left P3-M3) of CBI-1-565; **D**, drawings of the lower toothrow (left p3-m3) of CBI-1-33. Scale bars: 1 mm. Original scientific drawings by Laurence Meslin (© CNRS-Meslin).

**Figure 3:**
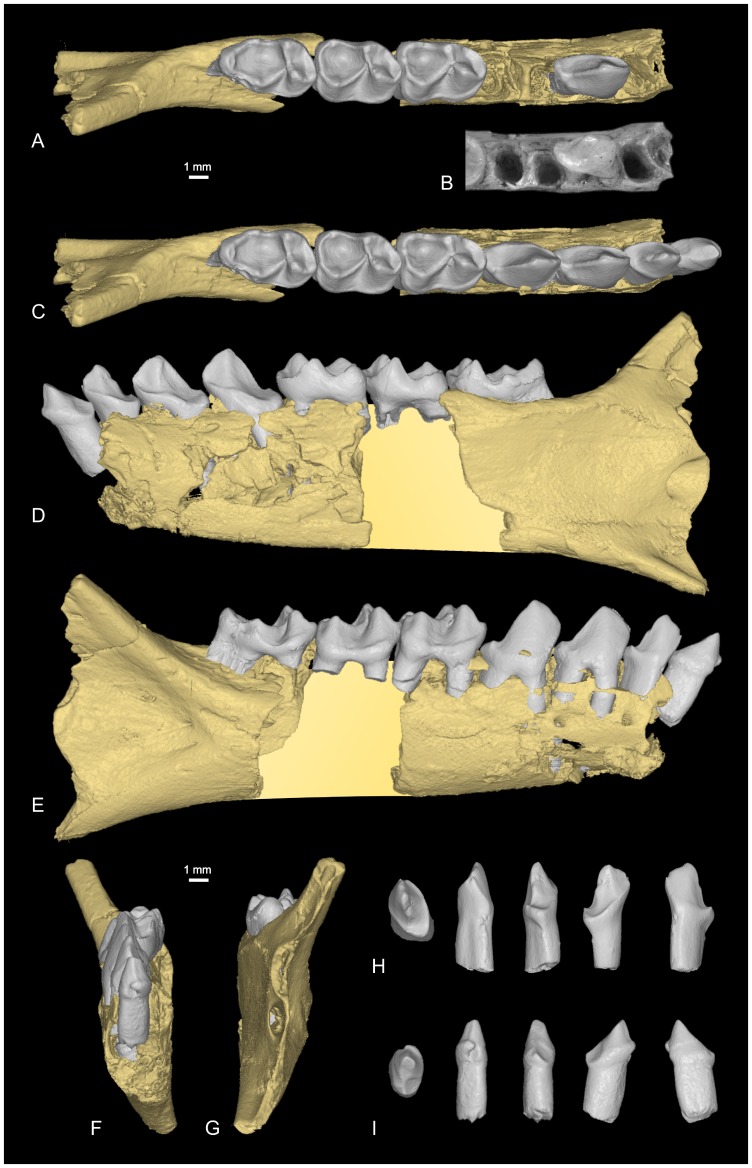
Lower jaw of *Djebelemur martinezi* from the Djebel Chambi CBI-1 locality. **A**, CBI-1-565, fragments of right mandible, which consists of three isolated pieces found together and reassembled here: the anterior part of the dentary bears the p3 and m1, and alveoli for p4, p2 and c, while the posterior part preserves m3 and a portion of the ascending ramus; m2 was found isolated but in the same small calcareous block treated by acid processing; **B**, photograph of the proximal part of CBI-1-565 (for p4 and p3, note that their mesial alveolus is slightly offset buccally with respect to their distal alveolus; the single alveolus for p2 is mesiodistally compressed and oblique, while the alveolus for the canine, although only partially preserved, appears slightly larger, suboval, and more lingually positioned with respect to the main axis of the toothrow). **C–G**, composite lower toothrow with the CBI-1-565 mandible, CBI-1-580 canine (reversed), CBI-1-587 p2 (reversed), and CBI-1-577 p4, in occlusal (**C**), lingual (**D**), frontal (**F**), and distal (**G**) views; **H**, CBI-1-587, left p2 in (from left to right) occlusal, buccal, lingual, distal, and mesial views (not reversed); **I**, CBI-1-580, left canine in (from left to right) occlusal, buccal, lingual, distal, and mesial views (not reversed). The 3D representations of CBI-1-565 (**A**, **C**–**G**), CBI-1-587 (**H**), and CBI-1-580 (**I**) have been obtained by X-ray µCT surface reconstruction. The crown and roots of teeth of the mandible have been virtually delimited by manual segmentation.

**Figure 4:**
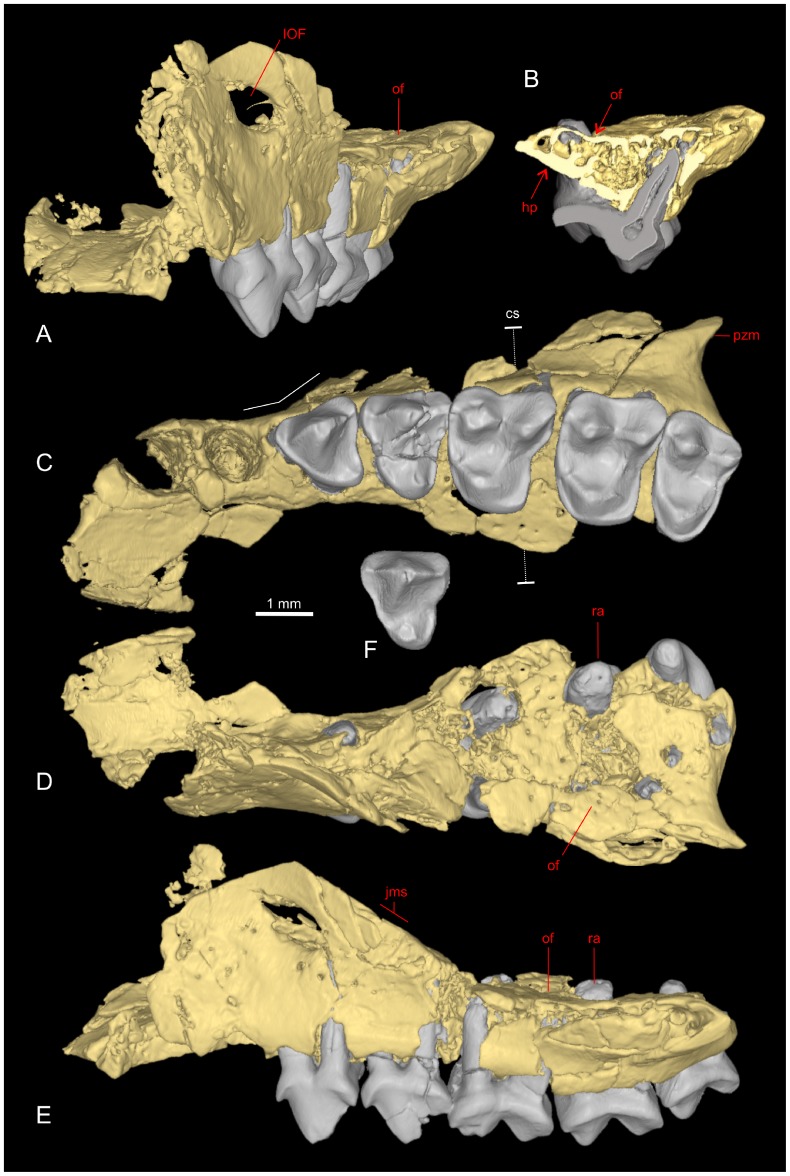
Facial fragment of *Djebelemur martinezi* from the Djebel Chambi CBI-1 locality. **A–E**, CBI-1-544, left maxilla preserving P3-M3 and alveoli for P2 and C1, in frontal (**A**), coronal section (cs) through M1 (**B**), palatal (**C**), dorsal (**D**), and lateral (**E**) views; **F**, CBI-1-567, left P4 in occlusal view. The 3D representations of CBI-1-544 and CBI-1-567 have been obtained by X-ray µCT surface reconstruction. On the maxilla, the crown and roots of teeth have been virtually delimited by manual segmentation. Abbreviations: **IOF**, infraorbital foramen; **of**, orbital floor; **hp**, hard palate; **pzm**, *processus zygomaticus maxillae*; **ra**, root apex; **jms**, jugo-maxillary suture.

In addition to the conventional scientific drawings of the fossil specimens (Fig. 2), we used X-ray microtomography (µCT scan) for obtaining three-dimentional digital model (3D rendering) of the fossils (Fig. 3 and Fig. 4). Each specimen was scanned with a resolution of 9 µm using a μ-CT-scanning station *Skyscan 1076* (Montpellier RIO Imaging). The crown and roots of each tooth have been virtually delimited by manual segmentation under Avizo software (VSG).

### Phylogenetic Reconstructions

The phylogenetic position of *Djebelemur* was investigated in a high-level primate phylogeny with a cladistic assessment of the craniodental and postcranial evidence. We mainly employed the morphological characters listed by Marivaux in Tabuce et al. ([11], modified after Kay et al. [3] and Marivaux et al. [7]). Characters were scored for 106 living and extinct taxa (see Datasets S1, S2 and S3, which are published as Supporting Information). We performed two kind of cladistic analyses, one considering some multistate characters as ordered, and another considering all characters as unordered. In the first case, multistate characters were considered as ordered if changes from one state to another required passing through intermediate states [46]. With such an *ad hoc* assumption, character state assignments do not convey *a priori* judgments about character polarity (unconstrained parsimony). With the same data set and character state assumptions (ordered *versus* unordered characters), we performed a second set of analyses, which were constrained by a molecular scaffold [47] to recover those primate clades that are supported by genomic sequences. The gene-based tree of modern taxa used as a scaffold was that published by Perelman et al. [28] (Dataset S4), which enforces the monophyly of the Lemuriformes (including a Lemuridae clade, a *Lepilemur*-Cheirogaleidae clade, and an Indriidae clade) and that of the Lorisiformes (including a Galagidae clade and a Lorisidae clade with an *Arctocebus*-*Perodicticus* subclade and a *Loris*-*Nycticebus* subclade). The phylogenetic reconstructions were performed with PAUP 4.0B10 [48] by heuristic searches using random step-wise addition (1000 replications with randomized input order of taxa) and tree-bisection-reconnection branch swapping options.

### Diet Reconstruction

For the analyses described below, we have observed and measured teeth of a set of extant primate specimens, which were available in the collections “*Mammifères & Oiseaux*” of the “*Muséum National d’Histoire Naturelle*” [MNHN], Paris, and in the collections of the Anthropological Institute and Museum [AIM], Zürich (Dataset S5). All necessary permits were obtained for the described study (measurements and molding), which complied with all relevant regulations.

#### Shearing quotient

Based on dental morphology, dietary habits can be predicted with some accuracy based upon the degree of development of molar shearing crests, quantified using a shearing quotient (SQ) [49]–[54]. Insects and leaves are mostly composed of chitin and cellulose, respectively, both of which are more resistant to digestion than fruit. Primates that eat them have long, sharp crests so as to be able to cut leaves and perforate chitinous exoskeletons. Conversely, frugivores have shorter crests and shallower basins so as to squash fruit. For each taxon considered in this study (extant and extinct [including *Djebelemur*]), six principal shearing crests were measured on the second lower molar (m2). For these measurements, we followed the protocol laid out by Anthony and Kay [53] and Kirk and Simons [54]. The sum of the lengths of the six shearing crests divided by the length of m2 corresponds to the SQ.

#### Microwear

The diet of *Djebelemur* was also reconstructed using dental microwear analysis, which used the combination of low magnification microscopy with digital capture for dental microwear analysis. It followed a strict casting protocol, designed to maximize image quality [55], [56]. This study follows the procedures described in Ramdarshan et al. [57], [58]. Photos were taken at 100 x using an optical stereomicroscope (Leica M 205C) connected to a camera (Leica DFC 420C). Image analysis was conducted with the open source software ImageJ (http://rsbweb.nih.gov/ij/) [59] and the plug-in ObjectJ (http://simon.bio.uva.nl/objectj/) [60]. For each specimen, a 100 µm×100 µm square was analyzed: every microwear feature was categorized as a pit, scratch, large pit or wide scratch. Features were then counted and measured (number of pits [Np], number of scratches [Ns], number of large pits [Nlp], number of wide scratches [Nws], and scratch length [Ls]).

#### Discriminant analysis

The resulting microwear data, associated with shearing quotients and body mass estimations were then analyzed using linear discriminant analysis (LDA). The LDA matrix (Dataset S5) consists of 7 quantitative variables (shearing quotients [SQ], body masses [BM], and microwear data [Np, Ns, Nlp, Nws, Ls]) and two qualitative variables (family and dietary category) measured on 15 taxa (strepsirhines and tarsiers): 38 Cheirogaleidae, 17 Lepilemuridae, 15 Indriidae, 20 Lemuridae, 43 Lorisidae, 46 Galagidae and 2 Tarsiidae. All dietary categories (Leaf-eaters, Insect-eaters, Fruit-eaters, and Gum-eaters) were considered as grouping factors in the discriminant analysis. These dietary categories have been shown to be statistically different in previous studies [58], [61]. The LDA matrix was analyzed with R-3.0.1 (R Development Core Team [62]).

## Results

### Systematic Paleontology

Order PRIMATES Linnaeus, 1758.

Suborder Strepsirhini Geoffroy, 1812.

Family Djebelemuridae Hartenberger and Marandat, 1992 (*sensu* Godinot, 2010 [36]).

Genus *Djebelemur* Hartenberger and Marandat, 1992.

#### Type species

*D. martinezi*, Hartenberger and Marandat, 1992.

#### Holotype

CBI-1-33 [41], a left dentary preserving p3-m3 and alveoli for p2 and c1, and partial ascending ramus (Fig. 2D).

#### Hypodigm

**Figure 5:**
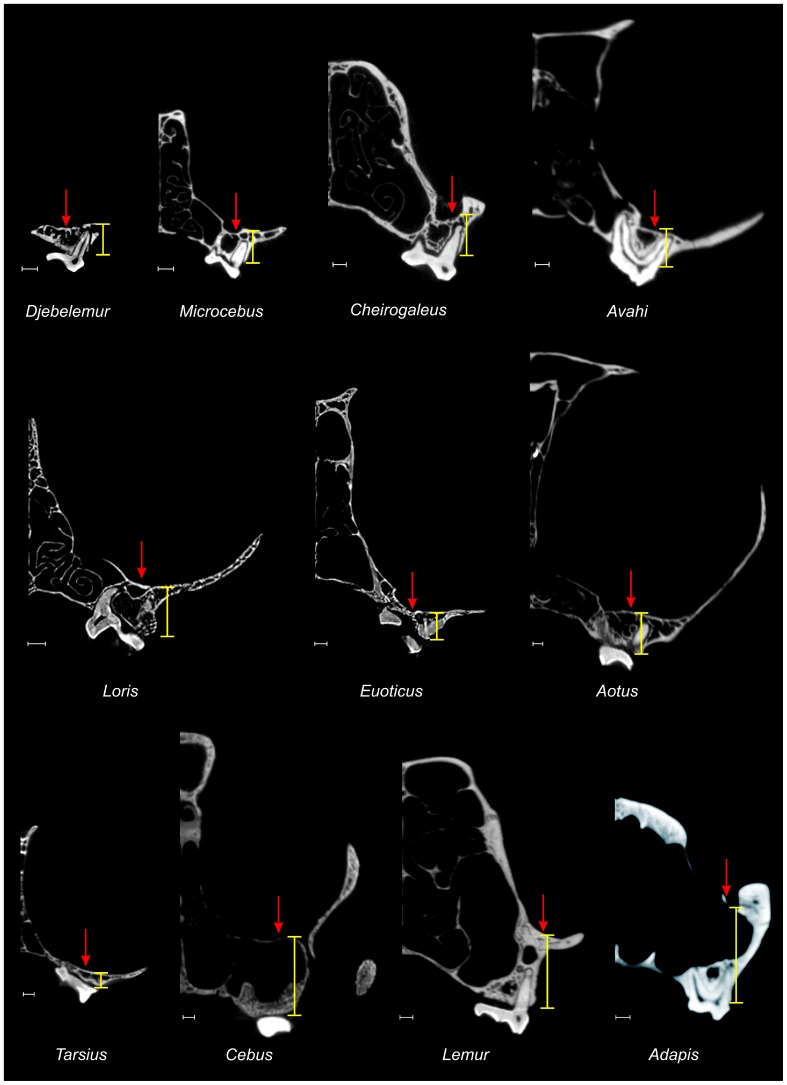
Comparative high-resolution micro-CT scans of the maxilla through the distal root of M1 in some selected nocturnal *versus* diurnal primates. *Microcebus*, *Cheirogaleus*, *Avahi*, *Loris*, *Euoticus*, *Aotus* and *Tarsius* are nocturnal primates, while *Cebus* and *Lemur* are diurnal (*Adapis* was most likely diurnal). The red arrows indicate the orbital floor, and the yellow bars provide an approximation of the suborbital depth of the maxilla. Scale bars: 1 mm.

**Figure 6:**
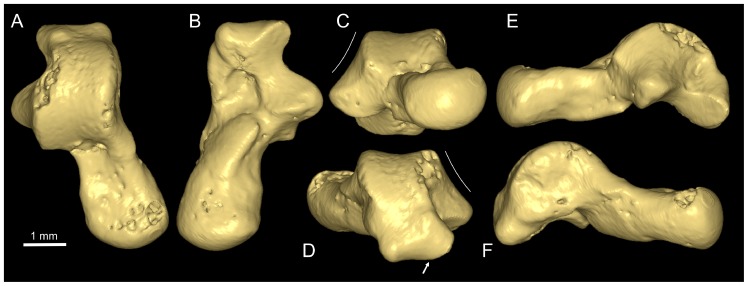
Ankle bone (or talus) of primate from the Djebel Chambi CBI-1 locality. **A–F**, CBI-1-545, right talus in dorsal (**A**), ventral (**B**), proximal (**C**), distal (**D**), lateral (**E**), and medial (**F**) views. The oblique white line (**C** and **D**) indicates the slope of the lateral talofibular facet. The white arrow (**D**) indicates the passage of the *flexor hallucis longus* tendon. The images are 3D digital models of the CBI-1-545 talus, which have been obtained by X-ray µCT surface reconstruction.

CBI-1-565, a damaged right dentary preserving m1–m2, p3, and alveoli for p4, p2 and c1 (Fig. 3A–B); CBI-1-14, a lower left canine; CBI-1-580, a lower left canine (Fig. 3I); CBI-1-579, a left p2; CBI-1-587, a left p2 (Fig. 3H); CBI-1-577, a right p4 (Fig. 3CE, included in the composite toothrow); CBI-1-578, a right p3; CBI-1-582, a right m1; CBI-1-584, a talonid of right m3; CBI-1-584, a talonid of right m2; CBI-1-544, a left maxilla preserving P3-M3 and alveoli for P2 and C1 (Figs. 2C, 4A–E and 5); CBI-1-566, a left P4 (Fig. 4F); CBI-1-567, a left P4; CBI-1-581, a left P3; CBI-1-545, a right talus (Fig. 6A–F); ? CBI-1-569-570, right petrosals; ? CBI-1-571, a left petrosal [40]. The specimens CBI-1-35-36 (M3 and M2), formerly attributed to *D. martinezi* by Hartenberger and Marandat [41] are no longer referred to this genus [38], [44], [63]. Metrics of the specimens are provided in Table 1.

**Table 1:** Measurements (in millimetres) of upper and lower teeth of *Djebelemur martinezi* from the Djebel Chambi CBI-1 locality.

Fossil cataloguenumber		Specimen	LengthMD	WidthBL
CBI-1-33	Left mandible(holotype)	p3	1.62	0.82
		p4	1.63	0.92
		m1	1.96	1.27
		m2	1.89	1.35
		m3	1.92	1.14
CBI-1-565	Right mandible(broken)	p3	1.64	0.93
		m1	2.01	1.38
		m2	1.99	1.40
		m3	2.19	1.25
CBI-1-580	Left lower canine	c	0.75	1.09
CBI-1-587	Left lower premolar	p2	1.10	0.77
CBI-1-579	Left lower premolar	p2	0.64	0.99
CBI-1-577	Right lower premolar	p4	1.63	0.98
CBI-1-578	Right lower premolar	p3	1.63	0.94
CBI-1-578	Right lower molar	m1	1.87	1.36
CBI-1-581	Left upper premolar	P3	1.41	1.28
CBI-1-566	Left upper premolar	P4	1.40	1.63
CBI-1-567	Left upper premolar	P4	1.50	1.67
CBI-1-544	Left maxilla	P3	1.44	1.52
		P4	1.49	1.75
		M1	1.75	2.15
		M2	1.73	2.29
		M3	1.39	2.19
CBI-1-544	IOF height = 0.71 mm			
	IOF breadth = 1.025 mm			
	IOF area = 0.57 mm^2^			
	SDLM1 = 0.89 mm			

The CBI-1-544 maxilla preserves most of the inferior orbital rim, which bears the infraorbital foramen (IOF). Teeth and IOF sizes were measured with a microscope fitted with a calibrated reticle (Measuroscope Nikon 10). MD, maximum mesiodistal length; BL, maximum buccolingual width; SDLM1, suborbital depth lingual to M1 (thickness of the bony laminae forming the floor of the orbit and the hard palate lingual to M1).

#### Type locality

Late Early to early Middle Eocene Chambi locus 1 (CBI-1), Djebel Chambi, Kassérine region, western part of Central Tunisia [42], [64] (Fig. 1).

#### Emended diagnosis

Tiny strepsirhine primate, similar in size to the modern cheirogaleid lemur *Microcebus rufus*; P1/p1 absent; P2/p2 small and single-rooted; P3-4 and p3-4 moderately sized; maxilla characterized by a very shallow sub-orbital depth (with molar roots penetrating through the orbital floor), a very anterior lateral broadening, and the presence of a large infraorbital foramen located directly above P3; P3-4 triangular in shape, with well-developed buccal cingulum bearing distinct mesial and distal styles; small and low protocone on P4, faintly visible on P3; upper molars with distal crown margin moderately notched, bearing equally sized and salient paracone, metacone and protocone, and without hypocone; paraconule small and metaconule absent; M1-2 with extensive stylar shelf development buccal to the metacone; short anterior cingulum, long posterior cingulum (without distolingual lobe), and lingual cingulum interrupted beneath the protocone (except on M3); thin, short and distally oriented postprotocrista; trigon basin closed distally by the development of a low, long and oblique metacrista connecting the base of the metacone to the postprotocrista; p3 and p4 equally sized, double-rooted (mesial root slightly offset buccally), with crown narrow and long, characterized by a single and prominent blade-like protoconid, and by a slight talonid elongation and complexity; p2 slightly reduced but with a similar structure than p3-4; p2-4 with a well-marked anterior crown elevation associated with an important tooth crowding; lower canine suboval in section with a buccolingually compressed, mesially elevated and oblique main cuspid; lower molars characterized by their trigonid slightly higher than their talonid; trigonid with well-developed premetacristid, complete postprotocristid and curved paracristid, making a continuous mesial loop encircling a mesial fovea; buccal paracristid running mesially than curving sharply with a distolingual orientation; deep talonid basin, closed lingually by the strong development of long postmetacristid and pre-entocristid; buccal cingulid interrupted beneath the hypoconid. Mean body-mass estimate of ∼ 70 g is based on m1 area (from “all-primate” and “prosimian” least-squares bivariate regression equations of Conroy [65].

### Description and Discussed Comparisons

#### Lower teeth

The morphology of lower molars and premolars of *Djebelemur* has already been described and compared in detail by Hartenberger and Marandat [41], and then by Godinot [35], [36], [44]. The most obvious and derived features of the lower molars is the structure of the trigonid, which includes a fovea built by the connection of the long and curved paracristid with a premetacristid (Figs. 2D and 3A). The premetacristid is generally absent in adapiforms but it is developed in most extinct and extant strepsirhines (lorisiforms and lemuriforms [S.*s.s.*]). On lower molars of *Djebelemur*, the course of the paracristid is also characteristic of strepsirhines (S.*s.s.*). This cristid runs mesially from the tip of the protoconid, and curves sharply, taking a slight or well-marked distolingual orientation (Figs. 2D and 3A). The morphology and arrangement of the premolars in *Djebelemur* are also on the same evolutionary path than in many crown strepsirhines.

p3 and p4 are equal in size and relatively small with respect to the molars. They are double-rooted and their mesial root is slightly offset buccally with respect to their distal root (Fig. 3B–E). These two premolars are narrow and long, with a simple morphology that is characterized by a trigonid with a single and buccolingually compressed cuspid (i.e., blade-like protoconid, Figs. 2D and 3C–E), and by a slight talonid elongation and complexity (without strong development of cristid and cuspid [presence of a minute hypoconid on p4]). Noteworthy is the degree of overlapping of the premolars associated with a degree of anterior crown elevation (Fig. 3D–E), which both indicate a process of premolar row compression as it does in the latest Eocene djebelemurid “*Anchomomys*” *milleri* [35], [36], [38], [66] and in most extinct (notably *Wadilemur* [8]) and extant strepsirhines (S.*s.s.*). Such an antemolar pattern differs from the condition observed in adapiforms (S.*s.l.*), which rather show a lengthening of their premolar row [35]. The holotype CBI-1-33 (Fig. 2D) and the new mandible fragment CBI-1-565 lack the front dentition, but mesially, they preserve two tooth root sockets (Fig. 3A–B). In the original description of the CBI-1-33 mandible, Hartenberger and Marandat (1992: Fig. 1b [41]) identified three mesial alveoli using X-rays. However, the distal most alveolus, initially identified as a root of p2, is actually the broken section of the mesial root of p3. On CBI-1-565, the distal alveolus is mesiodistally compressed and oblique, while the mesial one (although only partially preserved) appears slightly larger, suboval, and more lingually positioned with respect to the main axis of the toothrow (Fig. 3B). Given the shape, the relative size and position of these two root sockets, we are confident that only the second premolar was present on CBI-1-565 (and also on CBI-1-33), and that this tooth was small, single-rooted, and abutting a small canine, which was slightly offset lingually with respect to the premolar row.

Interestingly, we have found two isolated teeth in the CBI-1 locality, the size and morphology of which match those that might be expected for the p2 and canine of *Djebelemur*. This isolated left p2 (Fig. 3H) has its root mesiodistally compressed and a particularly narrow crown, which twists buccally to the root orientation, thereby making the crown mesiodistally elongated. This tooth shows a very similar anterior crown elevation with a blade-like protoconid as that observed on p3 and p4 of the two mandibles. The isolated canine (Fig. 3I) is not as narrow as p2 and appears more rounded in section (suboval, with a mesiodistal long axis), but it exhibits a similar tendency of anterior crown elevation with a blade-like main cuspid. So, if our dental locus attribution on this shallow dentary is correct (Fig. 3C–E), it is clear that *Djebelemur* did not have a lower anterior dentition as specialized as that characterizing many lemuriforms and lorisiforms (S.*s.s.*). In these latters (crown strepsirhines), the lower front dentition is radically modified, showing two incisors and one “incisiform” canine, which are reduced, elongated, procumbent, and closely appressed to form a tooth-comb. In many modern species, this structure is generally coupled with a p2, which is shaped like a typical canine (“caniniform”) and normally higher and/or larger than the other premolars. The new lower dental evidence gathered for *Djebelemur* indicates that this primate exhibited an antemolar pattern that could have represented, as for “*A.*” *milleri* [38], an intermediate morphological condition between the adapiforms (S.*s.l.*) and crown strepsirhines (lorisiforms and lemuriforms; S.*s.s.*).

#### Upper teeth

The maxilla of *Djebelemur* (CBI-1-544) provides the first and only evidence of the genus’ upper dentition. It is damaged but subcomplete and undistorted (Fig. 4A–E), and preserves P3-M3 and two alveoli. The most mesial alveolus, although only partially preserved, is large and suboval with a mesiodistal long axis, and is interpreted here as the alveolus for C1 (Fig. 4C). The single alveolus for P2 is smaller, rounded, and lies directly distal to the canine alveolus. A single-rooted P2 is also observed in azibiids (*Azibius* and *Algeripithecus*) known in the roughly contemporaneous sites of the Gour Lazib in Algeria [11], modern cheirogaleids, some lorisids (*Nycticebus*, *Perodicticus*), and in some advanced adapiforms (*Aframonius*, *Mahgarita*). In contrast, all other adapiforms known (adapids and notharctids), all galagids as well as *Loris* and *Arctocebus* (lorisids), lemurids and lepilemurids preserve a double-rooted P2, while indriids have no P2 (as well as the advanced African adapiform *Afradapis*). Despite the apparent lability of this character, it is worth noting that *Djebelemur* and azibiids have already achieved this dental trait early in the Tertiary.

The P3 is three-rooted and its crown is triangular in occlusal outline, being only slightly waisted distally and buccally (Fig. 4C). The crown of this tooth is dominated by a single buccal cusp (i.e., paracone) and is encircled by a low cingulum. This cingulum bears a small parastyle and metastyle on the mesiobuccal and distobuccal margins of the tooth, respectively, and a minute, faintly visible protocone on a small lingual lobe.

The P4 is slightly wider than P3, and differs in having a stronger protocone, which remains, however, lower to the paracone, and in having a low crista obliqua running from the base of the paracone to the protocone (Fig. 4F). In both teeth, the mesial and distal flanks of the paracone bear moderately developed pre- and post- paracristae. These two crests descend the faces of the paracone from its apex, and become confluent with the cingulum on P3, or with the buccal parastyle and metastyle on P4. P3-4 have neither metacone nor hypocone, and the protocone (especially on P4) is well inferior to the paracone. Such a remarkably simple, non-molariform, or even primitive structure differs substantially from the more specialized pattern characterizing P4 of azibiids, *Wadilemur*, some extant galagids (e.g., *Galago*, *Otolemur*), some lemurids (e.g., *Hapalemur*), some cheirogaleids (e.g., *Cheirogaleus*), and some adapiforms (e.g., *Leptadapis*, *Adapis*, *Sivaladapis*), which exhibit sub-molariform to molariform fourth premolar.

Molars of CBI-1-544 are particularly well-preserved (Figs. 2C and 4C). M1 and M2 are nearly similar in size, but differ in shape, M1 being longer buccally (more triangular), while M2 is wider (more rectangular and transverse). M3 is as wide as M1-2, but clearly narrower. Unlike adapiforms (except *Anchomomys gaillardi*) and azibiids, the distal crown margin of M1-2 of *Djebelemur* is moderately notched, as it is in the fossil strepsirhines (S.*s.s*) *Karanisia*, *Wadilemur* and *Saharagalago* (Late Eocene), and in most extinct and extant lorisiforms (galagids and lorisids). On CBI-1-544, the three molars have a simple structure, which consists of three equally sized and salient main cusps (paracone, metacone and protocone), with very low transverse crests. As in all extinct and extant strepsirhines (S.*s.s.*), there is no metaconule and the paraconule is small but distinct (absent on M3). On M1-2, the protocone is mesially canted, and displays a short postprotocrista, which ends at a point distal to the protocone, as is typically the case in *Karanisia*, *Wadilemur*, *Saharagalago* and *Omanodon* (an Early Oligocene taxon from Oman [67]). In addition, and similarly than in these latter taxa, as well as in azibiids, most extant strepsirhines (except *Cheirogaleus*, *Varecia* and *Lemur*) and in adapiforms (notably notharctids), there is a low, long and oblique metacrista ( = crista obliqua), which extends from the distal extremity of the postprotocrista to the lingual base of the metacone, thereby closing the trigon basin distally. Unlike early adapiforms and azibiids, the stylar shelf buccal to the metacone is particularly extensive in M1-2 of *Djebelemur*, and such stylar development resembles the condition found in *Wadilemur*, *Saharagalago* and *Karanisia*. M3 displays a well-marked and continuous lingual cingulum, while on M1-2, this structure is moderately developed and interrupted lingually, beneath the protocone. Mesially, the anterior cingulum is faintly developed (i.e., short) and limited between the protocone and paraconule. The anterior and lingual cingula are generally absent in extinct and extant lorisiforms as well as in their Eocene close relatives (*Wadilemur*, *Saharagalago*, and also *Omanodon*) and in some modern lemuriforms (lepilemurids and indriids), while this structure is well-developed and continuous in lemurids, cheirogaleids, and in *Karanisia*. In all adapiforms, the anterior cingulum is strongly developed, while the lingual cingulum is generally or variably interrupted, as it is the case in azibiids.

On molars of *Djebelemur*, the posterior cingulum is long and particularly well-marked, notably in its lingual part, but it does not form a prominent distolingual lobe as observed in *Karanisia*, *Wadilemur* and *Saharagalago*, or in extinct and extant galagids and lorisids. Furthermore, unlike azibiids and stem and crown strepsirhines (S.s.s., except *Cheirogaleus*), upper molars of *Djebelemur* have no hypocone. Upper molars without hypocone or with an incipient hypocone are found in early adapiforms [e.g., early Eocene *Cantius*, *Donrussellia*, *Asiadapis*, *Marcgodinotius*, and some middle Eocene species of *Anchomomys* (*A. gaillardi* [4] in which the hypocone is secondarily lost)], early omomyiforms, and early anthropoids (eosimiids and afrotarsiids). The development of the hypocone is particularly labile among primates, but the absence of this character in *Djebelemur* testifies here to the primitiveness of the dental pattern in this taxon. This condition contrasts markedly with that found in the coeval azibiids (*Azibius* and *Algeripithecus* [11]), which exhibit a highly specialized dental pattern, somewhat autapomorphous, with bunodont upper molars characterized by the development of a very strong hypocone. If azibiids may appear as an “aberrant” group of early stem strepsirhines (S.*s.s.*) having strong dietary specializations (see discussion below), the simple and primitive dental morphology of *Djebelemur* – which differs from that of early adapiforms – could document the dental bauplan of tooth-combed primates.

#### Facial fragment (maxilla)

CBI-1-544 preserves most of the inferior orbital rim, including a portion of the *processus zygomaticus maxillae*, and a portion of the jugo-maxillary suture, which is oblique in outline (Fig. 4E). The infraorbital foramen (IOF) is located directly above P3, and although slightly enlarged artificially due to breakage, it appears large (∼ 0.57 mm^2^; Fig. 4A; Table 1), somewhat relatively larger than the IOF of primates of such diminutive body size (e.g., *Galago demidoff* [∼ 60 g, ∼ 0.33 mm^2^], *Microcebus murinus* [∼ 90 g, ∼ 0.57 mm^2^], *Tarsius syrichta* [∼ 120 g, ∼ 0.34 mm^2^]; [68]). Given that the size of the IOF is a good proxy for the size of the nerve which innervates the maxillary mechanoreceptors [68], [69], the relatively large IOF of CBI-1-544 indicates that *Djebelemur* probably displayed a fine somatosensory acuity of the face, which was most certainly linked to the presence of numerous mystacial vibrissae.

In palatal view, the maxilla starts to broaden laterally at the level of the P2–P3 junction (Fig. 4C). By comparisons with extant and extinct primates, the loss of P1, the presence of a reduced P2, an IOF very anterior in position, and a very anterior lateral maxillary broadening, indicate that *Djebelemur* had probably a moderately short rostrum with laterally expanded orbits, as it probably did for azibiids (*Azibius* [11]). In lateral view (Fig. 4E), CBI-1-544 is very shallow dorsoventrally, particularly above the cheek teeth (beneath the orbital floor; Table 1). A coronal µCT scan section of the maxilla through M1 (Fig. 4B) shows that the suborbital space between the laminae of the orbital floor and the palate is very thin. The thinness of the plate formed by the subcomplete fusion of these laminae is such that the lingual root of the molars penetrates though the orbital floor, exposing them in the orbit (Fig. 4D–E). A strongly reduced mid-facial depth characterized by an orbitopalatal fusion and an exposure of the molar roots (the apex of which is blunt) in the orbital floor, is generally related to an eyeball hypertrophy [9], a condition that is observed in primates which exhibit nocturnality [70], [71]. Comparative coronal µCT scan sections of the maxilla through the distobuccal M1 root of some nocturnal and diurnal primates (Fig. 5) show that the degree of compression of the suborbital region (including an orbitopalatal fusion) in *Djebelemur* is comparable to that observed in *Euoticus*, and intermediate in condition between *Tarsius* and *Microcebus*, thereby suggesting very enlarged orbits in *Djebelemur*, probably associated with a nocturnal activity pattern.

#### Tarsal bone (talus)

CBI-1-545 is a complete and undistorted right talus from Chambi locus 1 (Fig. 6). The specimen is particularly well-preserved, except for the dorsal aspect of the talar body, which is slightly damaged in the proximal part of the lateral trochlear rim. Compared with ankle bones of extant primates, CBI-1-545 displays a set of derived anatomical features, which are highly diagnostic of strepsirhines (lorisiforms and lemuriforms) rather than haplorhines (tarsier and anthropoids). This is specifically shown in the inclination of the lateral talofibular facet (Fig. 6C–D), which slopes gently laterally over its entire extent, as it does also in extinct adapiforms for which the talus is known [1], [72], [73], [74]. The angle of the talofibular facet in CBI-1-545 (115°; Table 2) is however appreciably more obtuse than that observed in tali of most adapiforms, and is even one of the highest among crown strepsirhines [74]. This morphology contrasts markedly from the steep and straight-sided condition that characterizes the tali of anthropoids, *Tarsius*, and known extinct omomyiforms [1], [72], [75]. On CBI-1-545, the posteroventral region of the talar body bears a trochlear shelf, which is slightly grooved laterally for the passage of the tendon of the *flexor hallucis longus* muscle (Fig. 6D). A similar pathway in a lateral position can be seen in the tali of all strepsirhines (S.*s.s.*) and known adapiforms (S.*s.l.*), and diverges from the condition observed in extinct and extant anthropoids, tarsiids and known omomyiforms, in which this groove is plantad and in a midline position relative to the posterior trochlear facet [1], [72]. These two talar features (sloped talofibular facet and lateral groove for the flexor muscle tendon) clearly highlight the strepsirhine affinities of CBI-1-545.

**Table 2:** Metric features (in millimetres) of the CBI-1-545 talus from the Djebel Chambi CBI-1 locality.

Metric features		CBI-1-545	
Talar length	TL	5.23	
Talar neck length	NL	2.77	
Trochlear length	TRL	2.31	
Mid-trochlear width	MTRW	1.84	
Talar width^1^	TW	2.65	
Medial talar height^2^	MTH	2.41	
Medial talar body height	MTBH	1.69	
Lateral talar body height^3^	HT	2.15	
Talar head width^4^	HW	1.95	
Talar head height^5^	HHT	1.41	
Maximum ectal facet length	EFL	1.99	
Maximum ectal facet width	EFW	1.16	
Minimum ectal facet width	MEFW	0.71	
Talar neck angle^6^	T-Neck-angle (°)	20	
Talar head torsion^7^	T-Head-angle (°)	5	
Ectal facet orientation^8^	Ectal-F-angle (°)	50	
Slope of fibular facet^9^	SFF-angle (°)	115	

This primate tarsal specimen is attributed to *Djebelemur martinezi*. T-Neck-angle and talar measurements follow the works of Gebo et al. [75]. The angle between the fibular facet and lateral tibial facet (SFF-angle) is after Boyer et al. [73]. The talus was measured with a microscope fitted with a calibrated reticle (Measuroscope Nikon 10).

1 Distance from the most lateral point on the fibular facet (laterally projecting talar process) to the most medial point on the tibial facet.

2 Perpendicular distance from the most dorsal aspect of the medial trochlear margin to a chord connecting the most plantar point on the medial talar body to the plantar aspect of the talar head.

3 Perpendicular distance from the most dorsal point on the lateral trochlea margin to the chord defining the most plantar extent of the anterior and posterior aspects of the ectal facet.

4 Maximum mediolateral width.

5 Maximum dorsoplantar height.

6 Medial deviation of the talar neck relative to the anteroposterior axis of the trochlea.

7 Dorsolateral rotation of the talar head relative to the mediolateral axis of the dorsal trochlea.

8 Position of the posterior calcaneal facet relative to the talar neck.

9 Angle between the plane of the fibular facet relative to that of the lateral tibial facet.

Linear measurements of various anatomical talar features are provided in Table 2. Some talar dimensions of CBI-1-545 (i.e., TL, TW, MTRW, and HT) indicate a small primate having a body mass ranging from 50-80 g (estimated from the equations provided by Dagosto and Terranova [76] based on the “all strepsirhine” bivariate regression of talar dimensions against body mass in living primates). These estimates suggest that CBI-1-545 belonged to a tiny animal about the size of a living brown mouse lemur (*Microcebus rufus*, 82–92 g [77]). The first lower molar areas of *Djebelemur martinezi* provide mean body mass estimates of about 70 g (from the “all-primate” and “prosimian” least-squares bivariate regressions presented by Conroy [65]). The CBI-1-545 talus would therefore be appropriate in size to belong to *D. martinezi*. However, in Chambi locus 1 (CBI-1), the existence of another tiny primate is attested by the presence of two isolated teeth (CBI-1-35-36, slightly smaller in size to those of *Djebelemur* and originally described as pertaining to this taxon [41]) belonging to a taxon close to *Algeripithecus* [38], [44], [63]. The possibility exists that CBI-1-545 belongs to this *Algeripithecus*-like species and not to *D. martinezi*. However, the dental material referable to *D. martinezi* is clearly much more abundant at Chambi CBI-1, and the talar morphology of CBI-1-545 differs in some anatomical details (see below) from that of the azibiid tali (*Azibius* [39]). Given these considerations, the referral of CBI-1-545 to *D. martinezi*, seems to be the most appropriate taxonomic option here.

In overall morphology, CBI-1-545 appears tall, elongated and relatively narrow. The neck is clearly longer than the trochlea, it narrows proximally, and it is only slightly deflected medially from the body (Fig. 6A–B; Table 2). Ankle bones characterized by long necks are found primarily in small active quadrupeds such as cheirogaleid and lepilemurid lemuriforms, and galagid lorisiforms (see Dataset S6). In these talar neck features, CBI-1-545 differs substantially from all adapiforms for which the talus is known, from other quadrupedal lemuriforms, and from the slow climbing lorisiforms (i.e., lorisids), all of which generally show relatively shorter and more deflected necks (see Dataset S6). In anterior view, the head of CBI-1-545 is ovoid (nearly flat dorsally) and faintly rotated dorsomedially (Table 2). The trochlea is only moderately grooved and displays rounded, symmetrical and almost parallel medial and lateral trochlear rims, which are tightly curved dorsoproximally (Fig. 6C–E). The characteristics of the neck and the trochlea, associated with a moderately tall body and the presence of a strong posterior trochlear shelf indicate that leaping was certainly included in the locomotor repertoire of *Djebelemur*, in so far as such talar features are generally observed in quadrupedal primates who leap frequently [72]. However, in CBI-1-545 the trochlea is not deeply grooved and its rims are not as sharp as those of highly specialized leapers such as *Galago* and *Tarsius*, where only one primary plane of movement is needed at the talocrural joint (flexion and extension motions of the foot in a parasagittal plane). The rather flat (but not wedge-shaped as in azibiids) trochlea associated with a sloping fibular facet, likely allowed some degree of upper joint mobility. Plantarly, the ectal facet is moderately long and narrow, and it is oriented at roughly 50° to the talar neck (Fig. 6B). Its lateral and medial margins are deeply indented near their midpoint. This proximal plantar facet exhibits a small radius of curvature, which probably allowed a normal degree of subtalar motions, as those characterizing generalized arboreal quadrupedal primates. Finally, the medial tibial facet is extensive, deep and excavated (Fig. 6F). It extends only slightly distally onto the medial aspect of the neck, but does not flare medioplantarly to form an efficient tibial stop as in azibiids.

In sum, all these talar features characterizing CBI-1-545 are functionally more generalized than the more specialized talar anatomy of frequent leapers or climbers. These features, somewhat functionally intermediate, indicate a greater emphasis on arboreal quadrupedalism in *Djebelemur*, which was also capable of leaping with some ability, and of climbing, but to a lesser extent. In many ways, the talar morphology of CBI-1-545 differs substantially from that of adapiforms, and it is clearly not as specialized as that of most extinct and extant strepsirhines (S.*s.s.*, except cheirogaleids). The main apparent and somewhat advanced specialization of the CBI-1-545 talus could be seen in the orientation of the fibular facet, which is strongly inclined (very obtuse angle). It is worth noting that this condition of the slope of the talofibular facet in CBI-1-545 widely exceeds the angle reconstructed for the ancestor of crown Strepsirhini (i.e., 108.2°; [74]). Therefore, the presence of a markedly inclined talofibular facet in *Djebelemur* (and in azibiids [39]) would indicate that this condition of the lateral aspect of the talus was acquired early and rapidly in strepsirhines (S.*s.s.*) [74]. It has been suggested [78] that a sloping fibular facet has a weight-bearing function while the foot is grasping a vertical support (due to a more vertically oriented fibula), but also it provides a more open crurotarsal joint that allows better adduction-abduction mobility and higher angle of foot inversion; postures which are required by taxa using small diameter vertical and horizontal supports [74], [78]. The highly sloping fibular facet of the CBI-1-545 talus probably allowed enhanced lateral rotations of the upper ankle joint, which was associated with high angle of inverted foot grasping postures. Given the tiny body-size of *Djebelemur*, such an abducted and inverted foot postures indicate that this primate likely used very small-diameter supports. Despite this specialization, CBI-1-545 represents (as for azibiids) a primitive type of strepsirhine (S.*s.s.*) ankle anatomy. Cheirogaleids are generalized arboreal quadrupeds that include fast branch running, springing and climbing activities in their locomotor repertoire [79]. Given their small body-size and their talar gross morphology that is very close to that of the CBI-1-545 talus, without invoking possible close phylogenetic relationships, living cheirogaleids might appear as good analogues for *Djebelemur* in terms of locomotion (activities and positional behaviors). Interestingly, the morphology of the osseous inner ear of the CBI-1-569 petrosal (potentially referred to *Djebelemur* [40]), notably the variance from orthogonality of the plane of the semicircular canals, indicates that this primate included rapid head rotation in its way to move, as it is observed (calculated) in the cheirogaleid *Microcebus*, which exhibits fast locomotor head rotations [80].

### Phylogenetic Analyses

**Figure 7:**
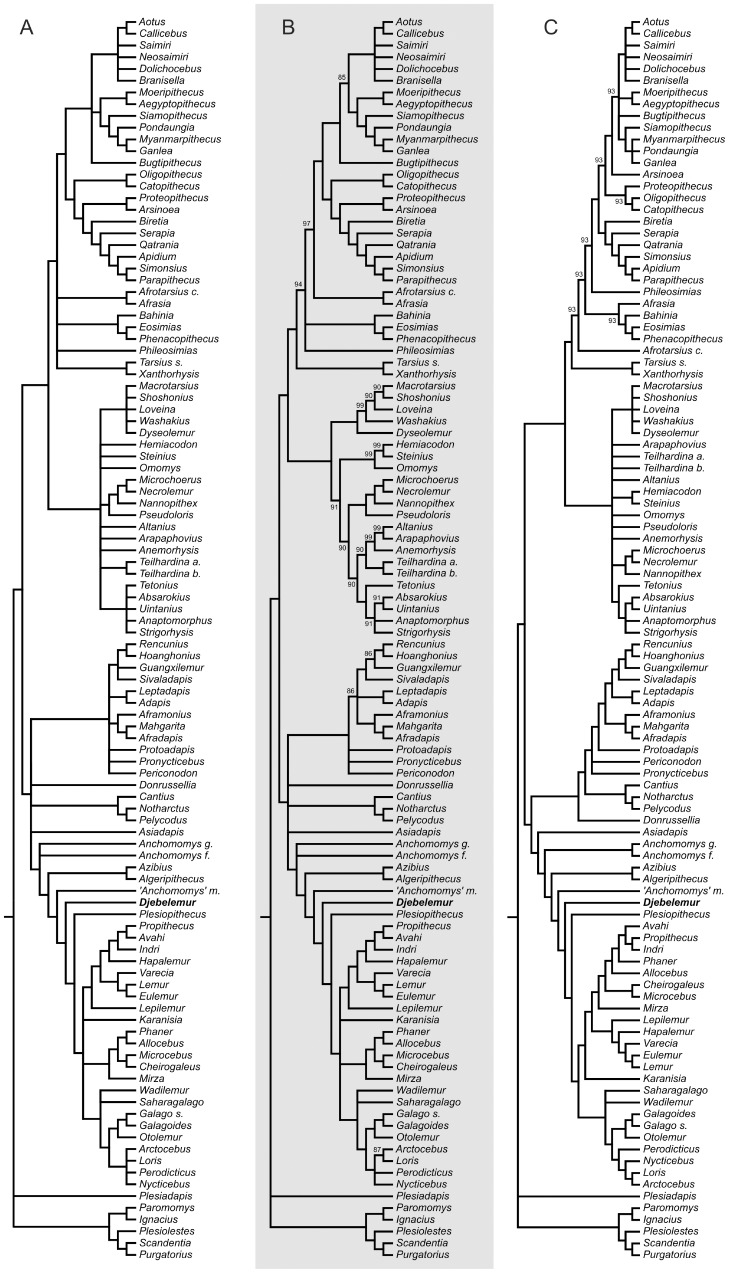
Phylogenetic position of *Djebelemur* in a high-level primate phylogeny with a cladistic assessment of the craniodental and postcranial evidence performed without molecular scaffold. **A–C**, consensus trees (**A**, Strict; **B**, Majority Rule at 80%) of 3426 equally most-parsimonious trees of 3886 steps each (CI = 0.147; RI = 0.566), which were obtained after analyses performed considering some multistate characters as ordered (i.e., if changes from one state to another require passing through intermediate states); **C**, consensus tree (Majority Rule at 80%) of 64 equally most-parsimonious trees of 3593 steps each (CI = 0.159; RI = 0.542), which were obtained after analyses performed considering all characters as unordered. On the Majority Rule consensus trees (B and C), the values above or below the nodes correspond to the rates (%) of node occurrences across the whole equally most-parsimonious trees found.

**Figure 8:**
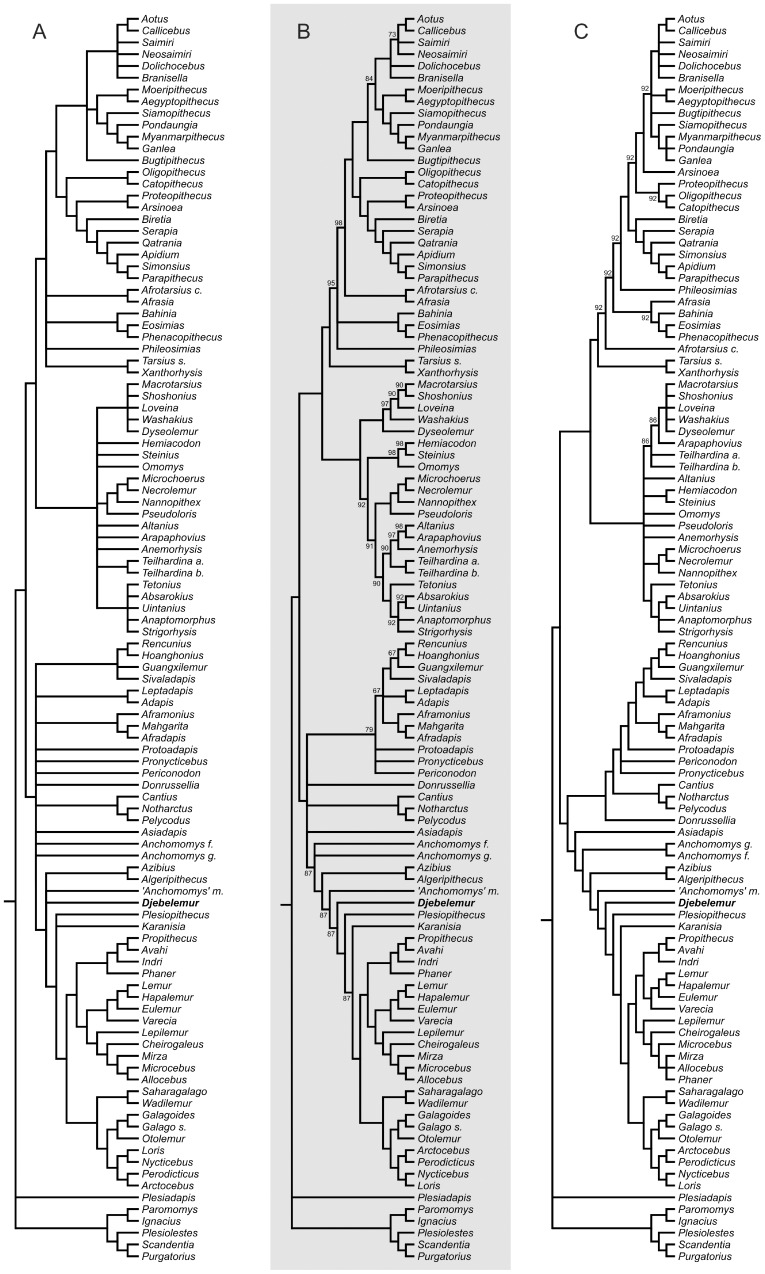
Phylogenetic position of *Djebelemur* in a high-level primate phylogeny with a cladistic assessment of the craniodental and postcranial evidence performed with a molecular scaffold. **A–B**, consensus trees (**A**, Strict; **B**, Majority Rule at 60%) of 506 equally most-parsimonious trees of 3903 steps each (CI = 0.147; RI = 0.564), which were obtained after analyses performed considering some ordered characters; **C**, consensus tree (Majority Rule at 80%) of 116 equally most-parsimonious trees of 3608 steps each (CI = 0.158; RI = 0.539), which were obtained after analyses performed considering all characters unordered. The molecular scaffold [47] was used to recover those primate clades that are supported by genomic sequences. This gene-based tree of modern taxa (Perelman et al. [28]) strongly supports the monophyly of Malagasy Lemuriformes (including a Lemuridae clade, a *Lepilemur*-Cheirogaleidae clade, and an Indriidae clade) and that of the Lorisiformes (including a Galagidae clade and a Lorisidae clade with an *Arctocebus*-*Perodicticus* subclade and a *Loris*-*Nycticebus* subclade). On the Majority Rule consensus trees (B and C), the values above or below the nodes correspond to the rates (%) of node occurrences across the whole equally most-parsimonious trees found. The molecular scaffold used here is as follows [28]: (Scandentia, ((((*Varecia*, (*Eulemur*, (*Lemur*, *Hapalemur*))), (*Lepilemur*, (*Cheirogaleus*, (*Microcebus*, *Mirza*))), (*Propithecus*, *Avahi*)), (((*Loris*, *Nycticebus*), (*Perodicticus*, *Arctocebus*)), (*Galago*, *Otolemur*))), (*Tarsius*, (*Saimiri*, *Aotus*)))).

We investigated the phylogenetic position of *Djebelemur* in a high-level primate phylogeny with a cladistic assessment of the craniodental and postcranial evidence. Whatever the multistate character assumption selected (ordered *versus* unordered), the two set of phylogenetic reconstructions (standard and constrained) recovered topologies in which the North African azibiids (*Algeripithecus* and *Azibius*), *Djebelemur*, “*Anchomomys*” *milleri*, *Plesiopithecus* and *Karanisia* are deeply nested successively closer to crown Strepsirhini than to adapiforms (Figs. 7 and 8).

Analyses without molecular scaffold and considering some multistate characters as ordered (Fig. 7A–B) failed to recover the monophyly of the Malagasy Lemuriformes, placing the Cheirogaleidae clade (*Cheirogaleus*, *Microcebus*, *Mirza*, *Allocebus* and *Phaner*) either as the sister group of a clade including Lorisiformes and all other Lemuriformes, or as a basalmost clade of Lorisiformes. In contrast, analyses without molecular scaffold and considering all characters as unordered (Fig. 7C) recovered the monophyly of the Malagasy Lemuriformes in which cheirogaleids appear however as a paraphyletic group (pectinately arranged), close to the Indriidae clade.

The constrained analyses (using a molecular scaffold; Fig. 8A–C), did fundamentally not affect the results regarding the surrounding phylogenetic structure. In the analyses performed with some multistate characters considered as ordered (Fig. 8A–B), *Phaner* (which is not considered in the gene-based tree of modern taxa [28]) is removed from the Cheirogaleidae clade and placed close to the Indriidae, while it is member of the Cheirogaleidae clade in the analyses performed considering all character as unordered (Fig. 8C).

In all these analyses (Figs. 7A–C and 8A–C), the late Eocene *Wadilemur* and *Saharagalago* from Egypt are clearly crown strepsirhines, but appear here to be stem rather than crown Lorisiformes (not stem Galagidae as formerly suggested by Seiffert et al. [8], [9], [21]). Likewise, the late Eocene *Karanisia* from Egypt, which was initially considered as a crown lorisid by Seiffert et al. [14], was placed here as a stem strepsirhine (S.*s.s.*) (as subsequently proposed by Seiffert et al. [8], [9]), close to crown groups in most analyses (Figs. 7A–B and 8A–C), except in the unconstrained analyses considering unordered characters (Fig. 7C), in which it appears as the earliest offshoot of the Lemuriformes clade. Finally, our analyses reveal the paraphyly of the Adapiformes, notably because of the placement of the middle Eocene European cercamoniine notharctid (“true” *Anchomomys* species – tribe Anchmomyini) and the early late Eocene Asian asiadapine notharctid (*Asiadapis*) as the closest out-groups of the Strepsirhini clade (S.*s.s.*) outside from the clade clustering the other adapiform taxa.

### Diet Reconstruction

**Figure 9:**
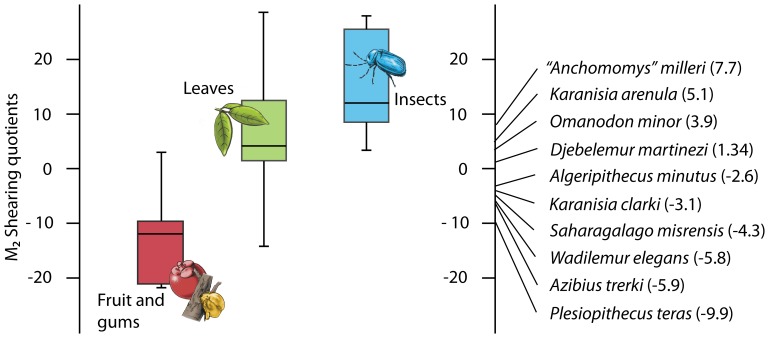
Comparison of the shearing quotients (SQ) of the different Eocene strepsirhine primates (non Adapiformes) from Africa. For each taxon, the SQ value is the ratio of m2 length to summed lengths of six principal m2 shearing crests [53], [54]. Color-coded bars (blue, insects; green, leaves; red, fruit and gums) indicate principal dietary item for extant strepsirhine taxa [54]. Low values indicate a diet based on fruit, gums or even seeds, whereas a high SQ points towards a leaf or insect based diet.

*Djebelemur martinezi* shows shearing quotients (Fig. 9) comparable to extant strepsirhine insect-eaters such as *Arctocebus calabarensis* (shearing quotients based on m2 [54], [61]). Compared to Eocene strepsirhine taxa from Africa (Fig. 9), *Djebelemur* displayed better developed shearing crests than the coeval azibiids (*Algeripithecus* and *Azibius*) from the Gour Lazib, Algeria (Fig. 1), but also better than *Wadilemur elegans* and *Plesiopithecus teras* (L-41, latest Eocene [54]) or *Saharagalago misrensis* and *Karanisia clarki* (BQ-2, early Late Eocene) from the Fayum, Egypt (Fig. 1). All these former taxa are interpreted as having been fruit-eaters (Fig. 9). Only “*Anchomomys*” *milleri* (L-41, Fayum) and *Karanisia arenula* (DT-1, Late Eocene, Libya; Fig. 1) had a higher shearing quotient than *Djebelemur* (Fig. 9). Body mass of *D. martinezi* is estimated between 60 and 80 g, thereby suggesting this primate could not have incorporated any leaves in its diet [52], [54], [81].

**Figure 10:**
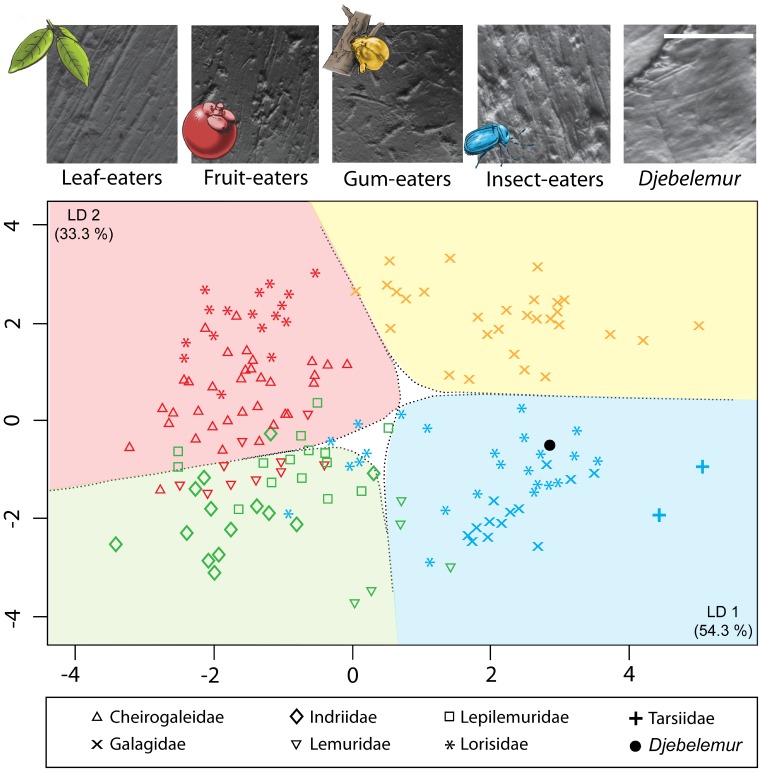
Linear discriminant analysis (LDA) based on dental microwear, shearing quotients and body mass estimations for extant strepsirhines and *Djebelemur martinezi*. Extant specimens are distinguished according to family: Cheirogaleidae (*Cheirogaleus major*, *Ch. Medius*, *Microcebus murinus*), Galagidae (*Galago senegalensis*, *Euoticus elegantulus*), Indriidae (*Propithecus verreauxi*), Lemuridae (*Eulemur fulvus*, *Hapalemur griseus*), Lepilemuridae (*Lepilemur ruficaudatus*), Lorisidae (*Perodicticus potto*, *Arctocebus calabarensis*, *Loris tardigradus*), and Tarsiidae (*Tarsius spectrum*). Dietary categories, generated by data on extant taxa, are delimited by LDA decision boundaries (dotted lines): leaf-eating is in green; fruit-eating in red; insect-eating in blue; gum-eating in yellow [61]. Photographs situated on the top of this figure are digitized images of microwear patterns for each dietary category and *Djebelemur martinezi* (top right: detail of the crushing facet of the protocone captured on the M2 of CBI-1-544). Photos were taken at 100 x using an optical stereomicroscope (Leica M 205C) connected to a camera (Leica DFC 420C). Scale bar = 100 µm.

Microwear patterns for this taxon show a high number of pits (Np = 37) and total number of microwear features (Ns = 29; Ls = 59.35; Nws = 1; Nlp = 3), making these patterns similar to those of extant insect-eaters [58], [61]. Results derived from the linear discriminant analysis (LDA) are plotted in Figure 10. The position of *Djebelemur martinezi* was predicted by projecting its corresponding transformed variables onto the linear discriminants. This prediction clearly shows that the diet of *Djebelemur* was primarily based on insects (posterior probability = 90.8%).

Additional insight into the dietary ecology of *Djebelemur* might also be provided by the size of the infraorbital foramen (IOF). Indeed, Muchlinski [68] has highlighted the correlation between the IOF area and certain ecological variables, and specifically diet. Frugivores have a significantly larger IOF than either folivores or insectivores, a finding that has led Muchlinski [68] to suggest that this anatomical structure could be useful for diet interpretations of fossil primates. As mentioned above, the maxilla of *Djebelemur* displays an IOF of 0.57 mm^2^, and as such it is greater than living primates of a similar size such as *Galago demidoff*, *Microcebus murinus* and *Tarsius syrichta* [68]. This could suggest *Djebelemur* incorporated a significant quantity of fruit in its diet. Although any dietary hypotheses must remain tentative in view of the low number of specimens, *D. martinezi* is described here primarily as an insect-eater, which most probably supplemented its diet with fruit.

## Discussion

### The Earliest Primates from Africa

Compared to North America and Eurasia, very little is known about the primates that inhabited Africa at the onset of the Tertiary [38], [82]. The only documentation for the earliest African primates has come from less than a handful of localities distributed primarily in Maghreb of North Africa (Morocco, Algeria, Tunisia [11], [41], [83]–[87] and one spot in sub-Saharan Africa (Namibia [88], [89]). Despite this very poor and scarce early record (Fig. 1), Africa has proven to be a critical land for early primate evolution and subsequent diversification of crown groups. In addition to *Altiatlasius* from the latest Paleocene of Adrar Mgorn (Morocco), which is one of the oldest crown Primates candidates, *Djebelemur*, *Azibius* and *Algeripithecus* from the late Early to the early Middle Eocene of Chambi (Tunisia) and Gour Lazib (Algeria), cf. ?*Altiatlasius* from the Middle Eocene of Aznag (Morocco), and *Namaia*, a primate of undetermined affinities from the middle Middle Eocene of Sperrgebiet-Black Crow (Namibia), are so far the only primate representatives for this early Tertiary interval in Africa. Another purported primate, relatively larger, could be also present in Chambi, but it is documented by only two isolated teeth, for which possible affinities with some advanced adapiforms (Asian sivaladapids) have been suggested, but also with non-primate hyopsodontid condylarths [90]. Based on our own observation of these two teeth, we think that the primate status of this taxon is far from being obvious in the absence of a more comprehensive fossil record, and we continue here to consider these fossils as belonging to “an enigmatic mammal” (*sensu* Court [90]). *Altiatlasius* remains a puzzling taxon due to its fragmentary nature, antiquity and geographic location (for a review, see Seiffert, 2012∶241 [38]). Nevertheless, putting aside the ongoing debate on its phylogenetic affinities, recent fossil discoveries [11], [39], [40] (this work) and re-analyses [8], [21], [35], [36] of these primates from the late Early to early Middle Eocene interval of Algeria and Tunisia have highlighted the primate diversity at that time in Africa (at least in North Africa). This diversity was actually much lower than previously thought, with only strepsirhine representatives (i.e., azibiids and djebelemurids). Indeed, if *Algeripithecus* and *Djebelemur* were in the past supposed to be allied somewhere with anthropoids (*Algeripithecus* [9], [44], [84], [85], [91], *Djebelemur* [44], [45]), these two taxa are now interpreted as closely related to crown Strepsirhini. In notable implications, this new fossil-based interpretation strongly forces rethinking the role of Africa as the ancestral homeland for anthropoids, and in turn, strengthens support not only for the hypothesis of an ancient diversification of tooth-combed primates in Africa, but also for the “Asiocentric” model of anthropoid origins [7], [11], [15], [92]–[98].

The end of the Eocene epoch (i.e., between ∼ 38 and 34 Ma; Fig. 1) is in contrast better documented regarding primates, primarily from North Africa with notably the Fayum Depression of northern Egypt (Birket Qarun Locality 2 and Jebel Qatrani Quarry L-41), the Dur At-Talah escarpment of the Sirt Basin in central Libya, and the Nementcha mountains of northeastern Algeria (Bir el-Ater). Over the past decades, fieldwork in these regions and rock units have revealed a high diversity and morphological disparity of the primate communities during this time period, including stem and basal crown strepsirhines [8], [14], [45], [66], [99], [100], adapiforms [10], [101], [102], and a wide array of anthropoids [9], [15], [45], [103]–[109]. If adapiforms could have been already present in Africa since the late Early Eocene [38], [90], undoubted anthropoids made their first appearance in the African fossil record only by the close of the Middle Eocene, likely after dispersing from Asia to Africa sometime during the Middle Eocene [7], [11], [15], [38], [109], [110].

The Eocene epoch in Africa is therefore particularly critical for primate evolution, notably for understanding the emergence of tooth-combed primates. Lorisiforms were already well-established by the Late Eocene (*Wadilemur* and *Saharagalago*), and the root of crown strepsirhines can now be traced back at least to the late Early Eocene with djebelemurids and azibiids, which are considered as pre-tooth-combed primates. Despite the fact that primates are virtually undocumented in Africa over a span of several million years (∼ 8–10 Ma; i.e., the major part of the Middle Eocene; Fig. 1), the presence of “advanced” stem strepsirhines (distinct from adapiforms) as early as the late Early Eocene in Africa better constrains the timing of the crown strepsirhine origin (i.e., Lorisiformes and Lemuriformes [S.*s.s.*]) to the Middle Eocene, rather than much earlier as estimated on molecular ground [19], [20], [23], [25]–[33].

Djebelemurids did not have an anterior lower dentition as specialized as that characterizing crown strepsirhines (i.e., “caniniform” p2 and procumbent tooth-comb complex made by the incisors and canines; except for *Daubentonia*), but they clearly exhibited an antemolar pattern that represents an intermediate morphological condition between the adapiforms (S.*s.l.*) and crown strepsirhines (S.*s.s.*). “*A.*” *milleri* is contemporaneous to the crown strepsirhine *Wadilemur* (i.e., latest Eocene [66]), but its lower dentition exhibits strong affinities with that of *Djebelemur*. “*A.*” *milleri*, which is 10–15 Ma younger than *Djebelemur*, could therefore represent a relict species of the djebelemurid lineage. The anterior dentition for *Azibius* and *Algeripithecus* is also unknown. However, one lower jaw of *Algeripithecus* preserves the anterior alveolus for the canine, which is deep, thin and forwardly inclined. In describing this alveolar pattern, Tabuce et al. [11] have suggested that the lower canine of *Algeripithecus* could have been incisiform and procumbent, and as such that a kind of incisor-canine functional unit could have existed in this stem taxa, a condition which would have predated the tooth-comb of crown strepsirhines. The existence of true tooth-combed primates at the time of *Djebelemur* and azibiids cannot be ruled out, but from our current knowledge of the African fossil record, no primate with such a derived front dentition as that of lorises and lemurs is so far documented, and only primitive transitional forms are recorded. These new fossil data rather suggest that the differentiation of a true tooth-comb must postdate the djebelemurid divergence (i.e., sometime during the Middle Eocene [35], [36]).

### The Origin of the Tooth-comb of Strepsirhine Primates

The body mass, microwear pattern, molar shearing quotient of *Djebelemur* (Figs. 9 and 10), as well as the size of its infraorbital foramen, indicate that this tiny djebelemurid primate from the late early to early middle Eocene of Tunisia was primarily an insect-eater, which most probably supplemented its diet with fruit. In contrast, *“Anchomomys” milleri* from the latest Eocene seems to have been more insectivorous, as this djebelemurid taxon displayed a higher shearing quotient than *Djebelemur* (Fig. 9). Although further studies are needed to confirm these dietary hypotheses, *Djebelemur*, and indeed “*A*.” *milleri*, seem to have incorporated more insects in their diet than azibiids (i.e., *Azibius* and *Algeripithecus*), for which insects were probably only a peripheral source of protein (Fig. 9). The recent and new lower dental evidence gathered for azibiids and djebelemurids indicate that both groups did not seem to have an anterior lower dentition as specialized as that characterizing crown strepsirhines, but they did exhibit an antemolar pattern which was on the same evolutionary path than in many crown strepsirhines (p2-4 narrow, long and simple with a blade-like protoconid; anterior crown elevation; tooth crowding; single rooted p2; deep, thin and procumbent canine root in azibiids), somewhat intermediate in condition between that characterizing adapiforms and crown strepsirhines.

Several hypotheses have sought to explain the origin and evolution of the tooth-comb in strepsirhine primates. This particular dental adaptation has previously been considered as significant in the acquisition of an exudates based diet. Indeed, Martin [111] suggested that a dietary transition towards exudate-eating could have been involved in the antemolar morphological transformations, which led to the emergence of the tooth-comb seen in crown strepsirhines. In this context, he described the anterior dentition as a tooth-scraper and that implied grooming (although an important function in primate ecology) was most probably secondary. However, other authors have noted the fragility of this dental complex [112]–[114], and favored grooming over diet as factoring in the origins of the tooth-comb.

Azibiids show very poorly developed shearing crests (Fig. 9) and very bunodont molars. Indeed, their molar morphology suggests fruit and/or gum-eating. Dental microwear analysis would allow us to distinguish between these two types of diet [58]. However, this analysis was not possible on these fossils, as most of the signal was erased during the chemical fossil extraction. Another clue as to the dietary ecology of azibiids might lie in their peculiar antemolar dental morphology. Indeed, in some living strepsirhine taxa, in addition to the anterior tooth-comb, premolars and upper canines are involved in acquiring exudates. For example, *Euoticus elegantulus* uses its high and buccolingually compressed canine and first upper premolar to reach difficult to access resources such as gums and exudates, which form the staple of its diet [115]. *Phaner*, also a specialized exudate-eater, displays a similar specialized morphology in its upper canine and first premolar [116]. Could the highly specialized, sometimes qualified as bizarre, morphology of azibiids correspond to an adaptation towards exudate-eating? Although different from those of *Euoticus* and *Phaner*, azibiid premolars are highly specialized. Both the upper and lower premolars are blade-like, long and serrated due to the development of large accessory cusps. This very peculiar morphology could represent an early adaptation towards gum-eating. Such an interpretation would be consistent with SQ results but only microwear analysis would truly confirm whether or not gum-eating may have factored into the development of such a peculiar arrangement of the front dentition that preceded the tooth-comb. It is also interesting to note that gum-eating is more commonly seen in nocturnal small bodied primates [116], which could have been the case for *Azibius* [11]. On the other hand, *Djebelemur* was also most probably nocturnal (this work) and results for this taxon would seem to point away from this type of diet. In this scenario, if this Tunisian primate was indeed an insect-eater, its pre-tooth-comb dental complex would not have been used towards gum-eating. This implies that either the adaptive origins of the tooth-comb lie elsewhere or that this complex had already moved away from its original exudate-acquiring function.

Other hypotheses have considered grooming to be instrumental in the emergence of tooth-comb morphologies, as this structure is essentially used towards that purpose in living strepsirhines [112]–[114]. Rosenberger [117] suggested that the lower anterior dentition were redirected from their role in feeding toward one in grooming. Leaf-eating in early primates would have freed up the anterior dentition from being used during feeding, which could then be used in different functions such as grooming. In this context, the ancestor of tooth-combed primates would have been a leaf-eater, with reduced upper incisors. Rosenberger [117] suggested that the strict folivorous dietary ecology seen in Eocene adapids such as *Adapis* and *Leptadapis* could have been a pre-adaptation, inasmuch as it would have relaxed selective pressures on the anterior dentition. However, the diet of late Eocene adapids may reveal to be more complicated that leaf-eating alone. Indeed, recent studies suggest a more complex dietary ecology, for which the anterior dentition might prove to have been useful [61], [118]. In this case, selective pressure on the anterior dentition would have still been in place. The African fossil record also needs to be considered in this context. Although the upper anterior dentition remains unknown for azibiids and djebelemurids, these primates were too small to have been able to feed on leaves (well-inferior to the Kay’s threshold [52]), and did not display the highly developed shearing crest pattern that would have been essential to process tough foods. Azibiids and *Djebelemur* clearly exhibited early stages of that particular lower antemolar adaptation characterizing lorises, galagos and lemurs, and given their dietary adaptation, it is likely that leaf-eating was not instrumental in the origin of the tooth-comb. However, this does not rule out grooming as factoring in the origin of the tooth-comb complex.

### The Root of the Tooth-combed Primates

Recent molecular clock estimates of crown strepsirhine origins generally advocate an early origin, near the onset of the Tertiary for Malagasy lemuriforms and Afro-Asian lorisiforms [25], [27], [29], [30]. Such an ancient antiquity of crown strepsirhines would imply that the differentiation of the tooth-comb occurred before or during the Paleocene (or even the earliest Eocene), and that the ancestral lemuriform colonized Madagascar by the earliest Tertiary [19], [20], [23], [28], [32]. Surprisingly, the same molecular estimates reveal that the main radiation of lemuriforms at the origin of almost all extant Malagasy lineages (Indriidae, Lepilemuridae, Cheirogaleidae, and Lemuridae, “except” Daubentoniidae) occurred in a time window coeval to that of the African lorisiform radiation (i.e., late Middle or Late Eocene [19], [20], [23], [28]–[30]). Interestingly, it appears that despite an early origin, the subsequent evolutionary histories of lemuriforms and lorisiforms in Madagascar and Africa, respectively, exhibit a vacuum of lineage diversification during most of the Eocene, followed by a relative acceleration in diversification from the late Middle Eocene. This apparent evolutionary stasis in the early evolution of crown strepsirhines is tentatively explained by the possibility of unrecorded lineage extinctions on both land masses during the early Tertiary [19], [23]. However, the presence of “advanced” stem strepsirhines (djebelemurids and azibiids) in the late Early Eocene of Africa strongly suggests that the origin of crown strepsirhines must postdate the djebelemurid divergence (i.e., sometime during the Middle Eocene [35], [36]). Furthermore, if we consider the ∼ 37 Myr old fossil *Karanisia* from Egypt, which seemingly displayed a “true” tooth-comb [14], [36], this taxon is neither clearly allied with Lorisiformes nor with Lemuriformes, and as such it appears as a tooth-combed stem strepsirhine of uncertain crown affinities [8], [9], [35], [36] (this work). All these fossil evidence documenting only stem strepsirhines during some part of the Eocene would hence preclude the existence of unrecorded lineage extinctions of crown strepsirhines during the earliest Tertiary as hypothesized by molecular data. This fossil-based hypothesis would imply also that the ancestral pioneer lemuriform (i.e., in the form of an already tooth-combed African stem strepsirhine, as *Karanisia* is) colonized Madagascar from Africa sometime during the Middle Eocene thank to the existence of a putative land-bridge at that time [119] or with some form of island-hopping process, or even by rafting [120] across the Mozambique Channel. This dispersal at that time would have been at the origin of crown strepsirhines (lemuriforms and lorisiforms). In that context, lemuriforms would have undergone their radiation in Madagascar by the late Middle to Late Eocene, just after their arrival on the island.

The question remains as to whether the daubentoniid lineage belongs to the same adaptive radiation than the other Malagasy lemuriforms. Some have suggested that the unique and somewhat odd living form of this family (*Daubentonia*) is representing another group of crown strepsirhines (i.e., Chiromyiformes *sensu* Anthony and Coupin, 1931 [121]), which is more closely related to the Lemuriformes *sensu stricto* (*s.s.*; Indriidae, Lepilemuridae, Cheirogaleidae, and Lemuridae) than to the Lorisiformes (Galagidae and Lorisidae). All DNA-based phylogenies show that the daubentoniid lineage is a very early offshoot of crown strepsirhines that diverged very shortly after the separation between the Lemuriformes *s.s.* and Lorisiformes, near the onset of the Tertiary owing to molecular clock estimates. Given that only primitive transitional forms of stem strepsirhines (not crown) are documented by the late Early or early Middle Eocene in the African fossil record, a very ancient divergence between Daubentoniidae and Lemuriformes *s.s.* seems inconceivable.

Recently, Godinot [35], [36] has proposed an interesting hypothesis regarding daubentoniid affinities and origins. In the cranium and mandibles of the plesiopithecid *Plesiopithecus*, a bizarre and very specialized strepsirhine from a terminal Eocene Fayum locality of Egypt (L-41 [98], [99]), Godinot has noticed an important number of morphological similarities with the living Malagasy aye-aye (*Daubentonia*). This fossil taxon exhibits a single enlarged and procumbent tooth preceding the premolar row (including four teeth: p1-4) on each side of the lower jaw. Although differing in some details, this peculiar and large front tooth was originally interpreted as the homologue and a larger version of the procumbent “incisiform” canine of crown strepsirhines [5], [45]. Godinot reinterprets the dental formulae of *Plesiopithecus*’ lower jaw in suggesting that its very large anterior tooth is more likely an incisor (as in *Daubentonia*), and that the following small tooth described as a p1 (present in only one mandible; DCP 11636) could be a “remnant of a disappearing canine” [36] “derived from a tooth-comb” [99]. Phylogenetically, *Plesiopithecus* appears closer to crown strepsirhines than djebelemurids and azibiids, a position which clearly underscores the tight linkage of this fossil taxon with the tooth-combed primate radiation. Due to its highly specialized, somewhat very odd cranial and dental morphology, it is still difficult to incorporate *Daubentonia* in phylogenetic analyses based on morpho-paleontological data [8], [9], [14], [108] (this work). Solving this pitfall might reveal that *Plesiopithecus* has perhaps some significance for daubentoniid origins in Africa, a hypothesis which would hence have critical implications on the patterns and timings of the Madagascar colonization by strepsirhine primates (i.e., two migration events of two closely related taxa [25], [35], [36]). This aspect of the early evolutionary history of Malagasy strepsirhines would need to be seriously investigated, but it requires to be substantially documented by fossils.

### The Possible Root of the Pre-tooth-combed Primates

*Djebelemur*, *Algeripithecus* and *Azibius* are the oldest representatives of two distinct but closely related African families of strepsirhines. Although poorly documented, “*A.*” *milleri* (Fayum, L-41 [66]; Fig. 1) as well as *Omanodon* (Oman, Taqah [67]; Fig. 1) show strong affinities with *Djebelemur* [8], [35], [36], [38], and are probably late diverging forms of the djebelemurid lineage. In many dental features, *Djebelemur* and azibiids were already distinct from coeval or slightly older European adapiforms (e.g., *Cantius*, *Donrussellia*, *Pronycticebus*, *Protoadapis*, and *Agerinia*), as they exhibited some morphological transformations documenting early stages “toward” a crown strepsirhine-like adaptation. In their original description, *Djebelemur* as well as “*A.*” *milleri* and *Omanodon* were considered as cercamoniine adapiforms due to their anchomomyin-like molar features (tribe Anchomomyini). This group of cercamoniines included few genera such as *Anchomomys*, *Buxella*, and *Periconodon* [122], which were common members of European primates faunas during some part of the middle Eocene (from the mid-Lutetian). However, if these European taxa shared superficial molar traits with djebelemurids and azibiids, they did not develop the derived premolar condition that characterizes these African stem strepsirhines. But we must consider that the molar resemblances between the European anchomomyin cercamoniines and the African djebelemurids-azibiids are perhaps a first step regarding the phylogenetic and geographic origins of the common ancestor of djebelemurids and azibiids, a scenario which is suggested by our phylogenetic results (Fig. 7).

Djebelemurids and azibiids, and subsequent crown strepsirhines, appear deeply nested in a cercamoniine radiation in Europe, and could share a common ancestry with some anchomomyins. However, the apparent diachronous development of these similar molar traits between the African and European taxa seems to argue in favor of a case of parallelism for explaining these dental resemblances. Considering the advanced degree of antemolar specializations of djebelemurids and azibiids at the time of Chambi or Gour Lazib deposits (late Early to early Middle Eocene; Fig. 1), it may be expected that these two primate groups have experienced an earlier phase of diversification in Africa during the Early Eocene. This would imply that the common ancestor of azibiids and djebelemurids colonized Africa from Europe or Eurasia much earlier, perhaps at the same time as the dispersal of *Altiatlasius* ancestors during the Paleocene, or during the global greenhouse warming characterizing the Paleocene-Eocene boundary [123]. It is now well-established that this rapid climatic event has involved environmental changes, which have favored numerous intercontinental dispersals of mammals, notably primates [124]–[127]. Very little is known about mammalian paleodiversity and evolution at that key time interval in Africa, and thus critical factors such as the mode of dispersal, the timing of interchange, and the pathways by which dispersal of land mammals occurred remain unknown. Only continuing field efforts in the early Paleogene deposits of Africa will allow for substantial discoveries of early African mammals, which should provide a better picture of the dawn of the tooth-combed primates, as well as some clues about their fascinating historical biogeography.

## Supporting Information

Dataset S1 **List of selected characters for the cladistic analyses.**
(DOC)

Dataset S2 **Taxa (genera and species) selected for the phylogenetic analyses.**
(DOC)

Dataset S3 **Matrix of the phylogenetic analyses.**
(RTF)

Dataset S4 **Molecular-Scaffold: gene-based tree of modern taxa deriving from Perelman et al. (2011)** [**28**] **.**
(RTF)

Dataset S5 **Matrix for the Linear Discriminant Analysis (LDA).** *Major component of the diet. The body masses are from Smith and Jungers [77]. Microwear variables: **Ns**, Number of scratches; **Np**, Number of pits; **Ls**, Length of scratches; **Nws**, Number of wide scratches; **Nlp**, Number of large pits. Institution abbreviations: **MNHN**, “*Muséum National d’Histoire Naturelle, Paris*”; **AIM**, Anthropological Institute and Museum, Zürich; **ONM**, “*Office National des Mines*”, Tunis.
(XLSX)

Dataset S6 **Talar ratio among primates (modified after Gebo et al.** [**75**] **, Marivaux et al.** [**39**] **).**
(XLSX)
