# Supplementary material for: Djebelemur, a Tiny Pre-Tooth-Combed Primate from the Eocene of Tunisia: A Glimpse into the Origin of Crown Strepsirhines
Source: PLoS One. 2013 Dec 4;8(12):e80778. doi: 10.1371/journal.pone.0080778 (PMC3851781; doi:10.1371/journal.pone.0080778)
Supplement: Dataset S1 — List of selected characters for the cladistic analyses. (DOC) [file pone.0080778.s001.doc]

**Dataset S1**

**List of selected characters for the cladistic analyses.**

Dental, cranial, and postcranial characters and character states used in the phylogenetic analysis. Characters followed by an asterisk are considered “ordered”. Some characters have been modified (different character state interpretation) from the original works (see Character references). These characters have been labeled by “ ***‘*** ” or “ ***“***”. A score of “ ? ” is used if information is unavailable due to a lack of material or if the character does not apply to a particular taxon. Tooth areas are calculated as the product of mesiodistal (md) length and buccolingual (bl) breadth.

##### Lower Teeth:

#### Incisors

1. i1*. Lower incisor number: 0 = three; 1 = two; 2 = one: I_1_ present, I_2_ absent; 3 = lower incisors absent.

2. i2. Lower incisor occlusal arrangement: 0 = arcuate battery from lateral perspective (U-shaped arcade); 1 = cusp tips staggered (V-shaped arcade).

3. i3. Lower incisor crown spacing: 0 = no spaces; 1 = spaces present between crowns.

4. i4. I_2_-C diastema: 0 = present; 1 = absent.

5. i5*. I_1-2_ size (ratio of I_1-2_ area to M_1_ area): 0 = very small (≤ 0.69); 1 = moderate sized (≥ 0.70, ≤ 1.07); 2 = large (> 1.07).

6. i6*. I_1_:I_2_ proportions (ratio of I_1_ area to I_2_ area): 0 = I_1_ much smaller than I2 (< 0.65); 1 = I_1_ smaller than I_2_ (≥ 0.65, < 0.82); 2 = I_1_ almost as large as I_2_ (≥ 0.83, < 1.00); 3 = I_1_ > I_2_ (≥ 1.01, < 1.25); 4 = I_1_ >> I_2_ (≥ 1.25).

7. i7*. I_1_ crown width (spatulate incisors only): 0 = considerably wider (m-d) than root (spatulate): 1 = narrow at apex, wider than root; 2 = “styliform” (crown apex approximately the same width as the cervical margin).

8. i8. I_2_ crown cross-sectional shape (ratio of m-d length to b-l breadth): 0 = rounded oval (≥ 0.64); 1 = mesiodistally compressed (< 0.64).

9. i9*. Lower incisors crown height (crown heights judged from cementoenamel junction to crown tip on the buccal surface): 0 = low crowned; 1 = moderately high crowned; 2 = high crowned.

10. i10. I_1-2_ crown buccal outline: 0 = gently curved in lateral perspective; 1 = acutely curved.

11. i11*. Lower incisor roots: 0 = erect or vertical; 1 = slightly procumbent; 2 = very procumbent.

12. i12*. Lower incisor crowns: 0 = erect or vertical; 1 = procumbent; 2 = very procumbent.

13. i13. Tooth comb: 0 = absent; 1 = with three teeth; 2 = with two teeth.

14. i14. I_1_ crown shape: 0 = spatulate; 1 = lanceolate, pointed.

15. i15. I_2_ heel development (a lingual swelling at the base of crown): 0 = heel absent; 1 = heel present.

16. i16. Incisor lingual enamel: 0 = well developed; 1 = very thin or absent.

17. i17*. Lower first incisor lingual cingulum: 0 = absent to weak; 1 = strong but incomplete; 2 = strong and complete.

18. i19*. Relative size of I_1_ to M_1_ (based on occlusal areas): 0 = I_1_ very small (I_1_ << M_1_); 1 = moderately enlarged (I_1_ < or = M_1_); 2 = grossly enlarged (I_1_ > M_1_).

#### Canines

19. c1*. Female C_1_ cross-sectional area relative to molar cross sectional area: 0 = very small (C_1_/M_1_ < 0.40); 1 = moderate (≥ 0.4, < 0.80); 2 = large (≥ 0.80, ≤ 1.20); 3 = very large (≥ 1.20)

20. c2*. C_1_/1 dimorphism (square root of male C_1_ area/square root of female C_1_ area): 0 = low (< 1.07); 1 = moderate (≥ 1.07, < 1.17); 2 = high (≥ 1.17).

21. c3. C_1_ cross-sectional shape: 0 = rounded oval; 1 = mesiodistally compressed; 2 = buccolingually compressed.

22. c4. C_1_ lingual crest development: 0 = rounded; 1 = sharp.

23. c5. Canine paracristid (not scored if species has canine incorporated into a tooth comb): 0 = oblique to occlusal plane; 1 = nearly horizontal to occlusal plane; 2 = forms part of cropping mechanism with I_1-2_.

24. c6. Canine height: 0 = low, squat; 1 = narrow, short; 2 = tall, at or above tooth row.

25. ML18*. Lower canine crown: 0 = erect or vertical; 1 = procumbent; 2 = very procumbent.

26. ML19*. Lower canine root: 0 = erect or vertical; 1 = slightly procumbent; 2 = very procumbent.

#### Premolars

27. p1. P^1^/1: 0 = present; 1 = absent

28. p2. P_2_: 0 = present; 1 = absent.

29. p3. P_2_ roots: 0 = single; 1 = double.

30. p4’. P_3_ roots: 0 = single; 1 = double.

31. p4”. P_4_ roots: 0 = single; 1 = double.

32. p5*. Premolar crowding (overlapping of crowns): 0 = no crowding; 1 = slightly crowded; 2 = very crowded—mesial root positioned buccal to distal root.

33. p6*. P_3_ paraconid: 0 = large; 1 = small; 2 = absent or extremely small.

34. p7*. P_4_ paraconid: 0 = large; 1 = small; 2 = absent or extremely small.

35. p9’*. P_4_ paraconid position (labiolingually = paracristid direction): 0 = mesial to protoconid; 1 = mesiolingual, between protoconid and metaconid; 2 = mesial to metaconid.

36. p9’’. P4 paraconid position (mesiodistally): 0 = widely spaced from the metaconid ; 1 = twinned with metaconid.

37. p11*. P_3-4_ cristid obliqua: 0 = absent; 1 = weak; 2 = strong.

38. p13. P_2_ protoconid height and shape: 0 = slender, projects above protoconids of P_3-4_; 1 = massive, projects above protoconids of P_3-4_; 2 = not projecting, in line with P_3_; 3 = extremely short, shorter than P_3_.

39. p14. P_4_ metaconid position: 0 = close to protoconid; 1 = widely spaced from protoconid (lingual); 2 = widely spaced from protoconid (distal).

40. p15. P_2_ metaconid size: 0 = absent or trace; 1 = small.

41. p16*. P_3_ metaconid size: 0 = absent or trace; 1 = small; 2 = large (as big as protoconid).

42. p17*. P_4_ metaconid size: 0 = absent or trace; 1 = small; 2 = large (as big as protoconid).

43. p18. P_4_ trigonid—configuration of lingual wall : 0 = closed; 1 = open.

44. p19. P_3_ entoconid and lingual talonid crest: 0 = absent; 1 = lingual talonid crest present but an entoconid does not stand out above it; 2 = entoconid forms a small discrete cusp.

45. p20. P_4_ entoconid and lingual talonid crest: 0 = absent; 1 = lingual talonid crest present but an entoconid does not stand out above it; 2 = entoconid forms a small discrete cusp.

46. p21. P_4_ lateral and medial protocristid: 0 = continuous between metaconid and protoconid; 1 = discontinuous between metaconid and protoconid.

47. p22. P_3_ lateral protocristid orientation: 0 = transversely oriented; 1 = distolingually oriented; 2 = absent.

48. p23. P_4_ lateral protocristid orientation: 0 = transversely oriented; 1 = distolingually oriented.

49. p24. P_3-4_ posterior trigonid wall: 0 = complete [taxa without metaconids are assigned this character state]; 1 = deeply notched.

50. p25. P_3-4_ hypoconid size: 0 = large; 1 = small or absent.

51. p26. P_3-4_ hypoconid (or distal terminus of oblique cristid) position: 0 = distal to protoconid; 1 = distal to metaconid, or between protoconid and metaconid

52. p27*. P_4_ hypocristid shearing development: 0 = absent; 1 = weak; 2 = strong.

53. p28*. P_2_ buccal cingulum development: 0 = absent; 1 = incomplete, broken at protoconid and hypoconid; 2 = complete.

54. p29*. Lower premolar inflation: 0 = not basally inflated; 1 = slightly basally inflated; 2 = very basally inflated.

55. p30*. P_4_ exodaenodonty: 0 = not exodaenodont; 1 = slightly exodaenodont; 2 = very exodaenodont.

56. p31*. P_4_ talonid length (ratio of midline m-d length of trigonid to m-d length of talonid): 0 = extremely short or non-existent (tri:tal ≥ 1.61); 1 = short (much shorter than trigonid) (tri:tal ≥ 1.27, < 1.61); 2 = equal or slightly shorter in length to trigonid (tri:tal ≥ 0.92, < 1.27); 3 = talonid longer than trigonid (tri:tal < 0.91).

57. p33*. Premolar orientation: 0 = Crown bases vertical in lateral perspective; 1 = slightly oblique; 2 = strongly oblique, projecting medial over the anterior.

58. p34. P_4_ anterobuccal cingulid development: 0 = absent or trace; 1 = strong.

59. p36*. P_4_ postprotoconid ridge: 0 = weak or absent; 1 = moderate; 2 = very strong.

60. p37*. P_4_ postmetaconid ridge: 0 = weak or absent; 1 = moderate; 2 = very strong.

61. p40*. P_4_ paraconid height: 0 = low; 1 = moderate; 2 = high (nearly as high as protoconid).

62. p41*. P_3-4_ protoconid height: 0 = P_3_ much lower than P_4_; 1 = P_3_ slightly lower than P_4_; 2 = P_3_ equal in height to P_4_; 3 = P_3_ higher than P_4_.

63. p42*. P_3_ to P_4_ area: 0 = 0.45-0.59; 1 = 0.60-0.69; 2 = 0.70-0.79; 3 ≥ 0.80.

64. p43*. P_4_ m-d L/ b-l W: 0 = (< 0.95); 1 = (≥ 0.96, < 1.14); 2 = (≥ 1.15, < 1.20); 3 = (≥ 1.21, < 1.35; 4 = (≥ 1.36, < 1.46); 5 = (> 1.47).

65. p44*. Ratio of P_4_ area to M_1_ area: 0 = (< 0.62); 1 = (≥ 0.63, < 0.72); 2 = (≥ 0.73, < 0.82); 3 = (≥ 0.83, < 0.92); 4 = (≥ 0.93, < 1.02); 5 = (> 1.03).

66. p45*. P_3-4_ root orientation: 0 = P_3-4_ roots aligned mesiodistally; 1 = P_3_ roots shifted laterally, P_4_ mesial root aligned mesiodistally; 2 = P_3-4_ shifted laterally. [Scored as missing if roots are single].

#### Molars

67. m1. M_3_: 0 = present; 1 = absent.

68. m2. M_1_ root number: 0 = one; 1 = two.

69. m3. M_2_ root number: 0 = one; 1 = two.

70. m4. M_3_ root number: 0 = one; 1 = two.

71. m6*. M_2_ trigonid width (ratio of buccolingual breadths of trigonid and talonid): 0 = much wider than talonid (≥ 1.11); 1 = widths similar (< 1.11, > 0.90); 2 = much narrower than talonid (≤ 0.90).

72. m7*. M_3_ trigonid width (based on relative buccolingual breadths): 0 = much wider than talonid (≥ 1.20); 1 = trigonid and talonid widths similar (≤ 1.20-1.05); 2 = trigonid narrower than talonid (< 1.05).

73. m8’*. M_1_ paraconid position: 0 = mesial to protoconid; 1 = mesiolingual, between protoconid and metaconid; 2 = mesial to metaconid.

74. m9’*. M_2_ paraconid position: 0 = mesial to protoconid; 1 = mesiolingual, between protoconid and metaconid; 2 = mesial to metaconid.

75. m10*. M_3_ paraconid position: 0 = mesial to protoconid; 1 = mesiolingual, between protoconid and metaconid; 2 = mesial to metaconid.

76. m8-9-10’*. M_2-3_ paraconid location: 0 = widely spaced from the metaconid; 1 = close to metaconid; 2 = twinned with metaconid.

77. m11. M_1_ parastylid (= premetacristid): 0 = absent; 1 = present.

78. m11’. M_2_ premetacristid: 0 = absent; 1 = present.

79. m12*. Molar metastylids (postmetacristids): 0 = absent; 1 = small; 2 = large.

80. m13. M_3_ hypoconulid: 0 = single; 1 = double

81. m14*. M_3_ heel: 0 = absent; 1 = narrower than talonid; 2 = approximately equal in width to talonid.

82. m15*. Molar enamel surface: 0 = smooth; 1 = slightly crenulated; 2 = highly crenulated.

83. m16*. M_1_ trigonid height (ratio of trigonid height to talonid height measured on the buccal aspect of the crown): 0 = higher than talonid (≥ 1.20); 1 = slightly higher than talonid (≥ 1.10, < 1.20); 2 = trigonid and talonid of similar height(< 1.10).

84. m17. M_1-2_ cusp relief: 0 = moderate to high; 1 = low.

85. m18. M_1_ trigonid lingual configuration: 0 = open; 1 = closed.

86. m19. M_1_ metaconid position: 0 = transversely aligned—lingual to protoconid; 1 = slightly distolingual to protoconid.

87. m20*. M_2_ paraconid development: 0 = absent; 1 = minute; 2 = small; 3 = large.

88. m20’*. M_1_ paraconid development: 0 = absent; 1 = minute; 2 = small; 3 = large.

89. m21. M_1-2_ lateral protocristid orientation: 0 = runs toward metaconid; 1 = runs toward hypoflexid.

90. m22. M_1_ distal trigonid wall: 0 = complete; 1 = deeply notched by protoconid/metaconid sulcus; 2 = medial and lateral protocristid do not meet but no sulcus is visible.

91. m23. M_2_ distal trigonid wall: 0 = complete; 1 = deeply notched by protoconid/ metaconid sulcus; 2 = medial and lateral protocristid do not meet but no sulcus is visible.

92. m24. M_1-3_ wear facet X: 0 = present; 1 = absent.

93. m25*. M_1-2_ entoconid: 0 = absent; 1 = barely stands out on lingual talonid marginal crest; 2 = a small discrete cusp; 3 = a large cusp.

94. m26*. M_1-2_ postentoconid sulcus: 0 = prominent; 1 = faintly visible; 2 = absent.

95. m27*. M_1_ hypoconulid size: 0 = large; 1 = moderate; 2 = small; 3 = absent.

96. m28*. M_2_ hypoconulid size: 0 = large; 1 = moderate; 2 = small; 3 = absent.

97. m29*. M_3_ hypoconulid size: 0 = large; 1 = moderate; 2 = small; 3 = absent.

98. m30*. M_1-2_ hypoconulid position: 0 = twinned to entoconid; 1 = near midline; 2 = slightly buccal to midline.

99. m31*. M_1-2_ cristid obliqua development: 0 = weak (rounded); 1 = strong (trenchant); 2 = very strong (trenchant).

100. m32*. M_1_ cristid obliqua orientation: 0 = reaches trigonid wall at a point distal to protoconid; 1 = reaches trigonid wall at a point distolingual to protoconid; 2 = reaches trigonid wall at a point distal to metaconid.

101. m33*. M_2_ cristid obliqua orientation: 0 = reaches trigonid wall at a point distal to protoconid; 1 = reaches trigonid wall at a point distolingual to protoconid; 2 = reaches trigonid wall at a point distal to metaconid.

102. m34. M_1_ cristid obliqua terminus: 0 = runs to base of trigonid; 1 = runs part way up the distal trigonid wall; 2 = connects with protoconid tip or protocristid; 3 = connects with metaconid.

103. m35. M_2_ cristid obliqua terminus: 0 = runs to base of trigonid; 1 = runs part way up the distal trigonid wall; 2 = connects with protoconid tip or protocristid; 3 = connects with metaconid.

104. m36. M_3_ cristid obliqua terminus: 0 = runs to base of trigonid; 1 = runs part way up the distal trigonid wall; 2 = connects with protoconid tip or protocristid; 3 = connects with metaconid.

105. m37. M_1-2_ centroconid development: 0 = present; 1 = absent but cristid obliqua bends sharply in hypoflexid; 2 = absent.

106. m38*. M_1-2_ hypocristid development: 0 = absent or seen only as a trace; 1 = weak; 2 = strong.

107. m39*. M_3_ hypocristid development: 0 = absent or seen only as a trace; 1 = weak; 2 = strong.

108. m40*. Lingual configuration of M_1-2_ talonid: 0 = open; 1 = notched lingually but not open; 2 = closed.

109. m41. M_1-2_ distal fovea: 0 = absent; 1 = present (weak); 2 = present (large).

110. m42. M_1-2_ hypocristid configuration: 0 = simple; 1 = with accessory cusp close to hypoconid.

111. m43. M_1-2_ cristid obliqua: 0 = notched; 1 = straight.

112. m44*. Molar cusp inflation: 0 = cusps not inflated, marginally positioned; 1 = slightly inflated; 2 = very inflated.

113. m45*. M_1-2_ buccal cingulid development: 0 = absent to trace; 1 = partial, broken at protoconid and hypoconid; 2 = complete.

114. m46*. M_1_ hypoflexid depth: 0 = very shallow; 1 = moderate; 2 = deep.

115. m47*. M_2_ hypoflexid depth: 0 = very shallow; 1 = moderate; 2 = deep.

116. m53*. Ratio of M_2_ length to M_3_ length: 0 = M_3_ much longer than M_2_ (0.71-0.80); 1 = M_3_ longer than M_2_ (0.81-0.90); 2 = M_3_ equal than M_2_ (0.91-1.00); 3 = M_3_ smaller than M_2_ (1.01-1.12); 4 = M_3_ much smaller than M_2_ (≥ 1.13); 5 = if M_3_ absent.

117. m55*. M_1_ mesiodistal length/buccolingual breadth: 0 = 1.0-1.15; 1 = 1.16-1.22; 2 = 1.23-1.32; 3 = > 1.33.

118. m56. Convergence of buccal and lingual molar cusp walls: 0 = convergent; 1 = vertically sided.

119. m57. M_1-2_ entoconid position relative to hypoconid: 0 = transverse to hypoconid; 1 = distal to hypoconid.

120. ML88*. M_1-3_ Pre-entocristid: 0 = indistinct to absent; 1 = weakly developed (low); 2 = well-developed (strong and high).

121. m58. M_1-2_ buccal paracristid orientation: 0 = oblique (mesiolingually oriented); 1 = lateral (distomesially oriented).

122. m59*. M_2_ buccal paracristid length: 0 = very short; 1 = short; 2 = long; 3 = very long.

123. m60*. M_2_ mediolingual paracristid orientation: 0 = transverse or oblique (buccolingually or mesiolingually oriented); 1 = oblique, backwardly directed; 2 = strongly oblique (strongly backwardly directed).

124. m61. M_2_ mediolingual paracristid elevation: 0 = very low (trace); 1 = low but well-marked; 2 = high.

#### **Upper Teeth:**

#### Incisors

125. I1*. I^1^-I^2^ interstitial contact: 0 = absent; teeth widely spaced; 1 = present as narrow contact; 2 = I^2^ tightly packed against I^1^, I^1^ preparacrista abbreviated.

126. I2. I^1^-I^1^ interstitial contact: 0 = present; 1 = absent: a wide space occurs in the midline between these teeth.

127. I3. I^2^-C diastema: 0 = present; 1 = absent.

128. I4*. I^1^ area:I^2^ area: 0 = areas approximately equal (≤ 1.00); 1 = I^1^ slightly larger than I^2^ (> 1.00, < 1.40); 2 = I^1^ much larger than I^2^ (> 1.40).

129. I5*. I^1^ size (I^1^ area: M^1^ area): 0 = incisor small (≤ 0.50); 1 = incisor moderate (> 0.50, < 0.56); 2 = incisor large (≥ 0.56).

130. I6*. I^1^ occlusal shape (mesiodistal length/buccolingual breadth): 0 = rounded oval (< 1.05); 1 = buccolingually compressed (> 1.05, < 1.30); 2 = extremely compressed (> 1.30).

131. I7*. I^2^ occlusal shape (mesiodistal length /buccolingual breadth): 0 = rounded oval (≤ 1.05); 1 = slightly buccolingually compressed (> 1.05, < 1.30); 2 = extremely buccolingually compressed ≥ 1.30).

132. I8. I^1^ crown shape: 0 = spatulate; no apparent occlusal cusp, mesial and distal edges continuous and rounded; 1 = semi-spatulate; central cusp present but blunt with discernable mesial and distal occlusal crests; 2 = central occlusal cusp pointed, occlusal edges steep.

133. I9. I^1^ lingual fovea: 0 = simple; 1 = dual, with mid-crown pillar.

134. I10. I^1^ occlusal edge orientation (for spatulate incisors only; all others scored as “ ? ”): 0 = occlusal edge orthogonal to long axis of root; 1 = occlusal edge wears at a steep angle to long axis of root; 2 = crown with pronounced mesial asymmetry (= mesial process) in unworn state.

135. I11*. I^1-2^ lingual cingulum: 0 = weak, discontinuous; 1 = moderate, continuous; 2 = strong.

136. I12. I^1^ basal lingual cusp: 0 = absent; 1 = present.

137. I13. I^1^-I^2^ buccal cingulum: 0 = absent; 1 = present.

#### Canines

138. C1. C^1^ cross-sectional shape: 0 = oval; 1 = rounded.

139. C2*. Upper canine occlusion: 0 = C^1^ wears against P_1-2_; 1 = C^1^ wears against P_2_; 2 = C^1^ wears against P_2-3_; 3 = C^1^ wears against P_3_.

140. C3. C^1^ mesial groove (females): 0 = shallow or absent; 1 = deep.

141. C4*. C^1^ lingual cingulum: 0 = weak or absent; 1 = strong; 2 = very strong.

#### Premolars

142. P1*. P^2^ root number: 0 = one (if tooth is absent, taxon scored “ 0 ”); 1 = two; 2 = three.

143. P2*. P^3^ root number: 0 = one; 1 = two; 2 = three.

144. P3*. P^4^ root number: 0 = one; 1 = two; 2 = three.

145. P4*. Ratio of P^2^ area to P^3^ area: 0 = P^2^ much smaller (≤ 0.85) (if tooth is absent, taxon scored “ 0 ”); 1 = P^2^ smaller (> 0.85, < 0.95); 2 = P^2^ equal (≥ 0.95); 3 = clearly larger.

146. P5*. Ratio of P^4^ area to M^1^ area: 0 = P^4^ << M^1^ (≤ 0.66); 1 = P^4^< M^1^ (> 0.66, ≤ 0.76); 2 = P^4^ = M^1^ (0.77-1.05); 3 = P^4^ > M^1^ (> 1.06).

147. P6. P^2^ occlusal outline: 0 = triangular; 1 = suboval with the long axis b-l; 2 = suboval with the long axis m-d; 3 = round.

148. P7. P^4^ occlusal outline: 0 = triangular; 1 = suboval; 2 = squared.

149. P8. P^3-4^ trigon/talon proportions: 0 = trigon > = talon; 1 = trigon < talon.

150. P9. P^3^ protocone: 0 = present; 1 = absent.

151. P10. P^4^ metacone: 0 = absent; 1 = present.

152. P11. P^4^ protocone: 0 = low relative to paracone; 1 = high relative to paracone.

153. P12. P^2^ protocone: 0 = present; 1 = absent (if tooth absent, taxon scored “ 1 ”).

154. P13’. P^2^ hypocone: 0 = absent; 1 = present.

155. P14*. P^4^ paraconule: 0 = large; 1 = small; 2 = absent.

156. P15. P^3-4^ parastyles: 0 = present; 1 = absent.

157. P16. P^3-4^ metastyles: 0 = absent; 1 = present.

158. P17. P^3-4^ postprotocrista: 0 =strong; 1 = weak, short.

159. P18*. P^2-3^ distal crown margin: 0 = smoothly rounded; 1 = slightly waisted between buccal and lingual cusps; 2 = strongly waisted between buccal and lingual cusps.

160. P18’*. P^4^ distal crown margin: 0 = smoothly rounded; 1 = slightly waisted between buccal and lingual cusps; 2 = strongly waisted between buccal and lingual cusps.

161. P19. P^3-4^ lingual cingulum: 0 = absent or weak; 1 = strong.

162. P20. P^3^ metacone: 0 = absent; 1 = present

163. P21. P^3-4^ buccal cingulum development: 0 = absent or weak; 1 = strong.

164. ML126*. P^4^ hypocone: 0 = minute to absent; 1 = present but small; 2 = strong.

165. ML127*. P^3^ hypocone: 0 = minute to absent; 1 = present but small; 2 = strong.

#### Molars

166. M1*. M^1-2^ root number: 0 = three, three; 1 = three, two; 2 = two, two.

167. M2*. M^3^ root number: 0 = three; 1 = two; 2 = one.

168. M3*. M^2^ shape (bl/md): 0 = very transverse (> 1.65); 1 = transverse (< 1.65, > 1.30); 2 = squared (≤ 1.30).

169. M4*. Ratio of M^1^ area to M^2^ area: 0 = M^1^ >> M^2^ (≥ 1.40); 1 = M^1^ > M^2^ (< 1.40, > 1.0); 2 = M^1^ ≤ M^2^ (≤ 1.0).

170. M6*. M^1-2^ pseudohypocone: 0 = absent; 1 = weak; 2 = strong.

171. M7’*. M^1-2^ metaconule: 0 = absent; 1 = single; 2 = double.

172. M9*. M^1-2^ preprotoconule: 0 = absent; 1 = weak; 2 = strong.

173. M10*. M^1^ hypocone size: 0 = large; 1 = small; 2 = minute to absent.

174. M11*. M^2^ hypocone size: 0 = large; 1 = small; 2 = minute to absent.

175. M12’*. M^1-2^ hypocone position: 0 = distal, far lingual to protocone; 1 = distal, slightly lingual to protocone; 2 = distal, slightly buccal to protocone.

176. M13*. M^1-2^ prehypocrista development: 0 = absent; 1 = weak; 2 = strong, reaches to postprotocrista, encloses the talon lingually.

177. M14*. M^3^ prehypocrista development: 0 = absent; 1 = weak; 2 = strong, reaches to postprotocrista, encloses the talon lingually.

178. M15. M^1^ or M^2^ paraconule position: 0 = attached to preprotocrista; 1 = unattached to preprotocrista.

179. M16*. M^1-2^ metaconule: 0 = absent to indistinct; 1 = small; 2 = moderate; 3 = large.

180. M16’*. M^1-2^ metaconule position: 0 = buccal (normal); 1 = lingually displaced; 2 = strongly lingually displaced (as lingual as the protocone).

181. M17’*. M^1-2^ mesostyle size: 0 = absent to indistinct; 1 = moderate; 2 = strong.

182. M17”. M^1-2^ mesostyle position: 0 = attached to ectocrista; 1 = present on buccal cingulum.

183. M20*. P^4^-M^1^ pericone: 0 = absent; 1 = small; 2 = large.

184. M22*. M^1-2^ lingual cingulum development: 0 = absent; 1 = faintly visible; 2 = well distinct; 3 = strong.

185. M22’. M^1-2^ lingual cingulum structure: 0 = mesiodistally complete; 1 = broken lingually (interrupted).

186. M24*. M^1-2^ buccal cingulum development: 0 = absent to indistinct; 1 = weak; 2 = strong.

187. M27*. M^1-2^ pre-metaconule cristae: 0 = absent to indistinct; 1 = short and weak; 2 = long and strong.

188. M28*. M^1-2^ post-metaconule cristae: 0 = absent to indistinct; 1 = short and weak; 2 = long and strong.

189. M30*. M^3^ paraconule: 0 = absent; 1 = small-moderate; 2 = large.

190. M31*. Molar protocone lingual inflation: 0 = not inflated; 1 = slightly inflated; 2 = very inflated.

191. M33*. M^2^ buccal expansion of paracone (specify which tooth): 0 = no expansion; 1 = slight expansion; 2 = considerable expansion.

192. M34*. M^3^ metacone: 0 = absent or very small; 1 = moderate (but smaller than paracone; 2 = large (equal to paracone).

193. M36*. M^3^ hypocone: 0 = absent or very small; 1 = small; 2 = large.

194. M37*. M^1^ paraconule size: 0 = absent; 1 = small-moderate (smaller than paracone); 2 = large (nearly as large as or larger than paracone).

195. M44*. M^1-3^ anterior cingulum: 0 = strong, complete, long (connected to parastyle); 1 = strong, short; 2 = weak or absent.

196. M46*. M^3^ size relative to M^1^: 0 = very small (half the size of M^1^ or less); 1 = small (two thirds); 2 = large (approximately as large).

197. ML147*. M^1-2^ metastyle: 0 = indistinct to absent; 1 = moderate; 2 = strong.

198. ML148*. M^1-2^ parastyle: 0 = indistinct to absent; 1 = moderate; 2 = strong.

199. ML149. M^1-2^ parastyle position: 0 = mesial to paracone; 1 = mesiobuccal to paracone.

200. ML150. M^1-2^ metastyle position: 0 = distal to metacone; 1 = distobuccal to metacone.

201. ML151*. M^1-3^ posterior cingulum: 0 = weakly developed; 1 = moderate, does not reach the metastyle; 2 = connected to metastyle.

202. ML151’*. M^1-2^ posterior cingulum lobe (distomedial) inflation: 0 = no inflation; 1 = slightly inflated; 2 = strongly inflated.

203. ML152*. M^1-3^ posterior margin (waisted between buccal and lingual cusps): 0 = indistinct to absent; 1 = present but shallow; 2 = present, deep.

204. ML153*. M^1-2^ postparacrista: 0 = indistinct to absent; 1 = weakly developed; 2 = well developed (but well-marked notch between postparacrista and premetacrista); 3 = strongly elevated (weak notch between postparacrista and premetacrista).

205. ML154*. M^1-2^ premetacrista: 0 = indistinct to absent; 1 = weakly developed; 2 = well developed (but well-marked notch between premetacrista and postparacrista); 3 = strongly elevated (weak notch between premetacrista and postparacrista).

206. ML155. M^1-3^ protocone arrangement: 0 = normal position; 1 = oblique.

207. ML156. M^1-2^ postprotocrista development: 0 = strong; 1 = tiny.

208. ML157*. M^1^ postprotocrista length: 0 = indistinct to absent; 1 = short; 2 = long.

209. ML158*. M^2^ postprotocrista length: 0 = indistinct to absent; 1 = short; 2 = long.

210. ML159. M^1^ postprotocrista direction: 0 = transverse, buccally directed; 1 = lateral, directed toward the lingual posterior cingulum (post-protocone fold-like).

211. ML160. M^2^ postprotocrista direction: 0 = transverse, buccally directed; 1 = lateral, directed toward lingual posterior cingulum (post-protocone fold-like).

212. ML161. M^1^ postprotocrista terminus: 0 = runs to base of metacone (with hypometacrista); 1 = runs to metaconule (at the level of the small or virtual metaconule); 2 = runs to posterior cingulum; 3 = limited at a point distal to protocone.

213. ML162. M^2^ postprotocrista terminus: 0 = runs to base of metacone (with hypometacrista); 1 = runs to metaconule (at the level of the small or virtual metaconule); 2 = runs to posterior cingulum; 3 = limited at a point distal to protocone.

214. ML163. M^1-2^ preprotocrista: 0 = low; 1 = elevated.

215. ML164. M^1^ preprotocrista connection (buccal side): 0 = connected between paracone and parastyle (by way of preparaconule crista); 1 = connected to parastyle (by way of preparaconule crista); 2 = connected to paraconule (or near to it or to a virtual paraconule).

216. ML165. M^2^ preprotocrista connection (buccal side): 0 = connected between paracone and parastyle (by way of preparaconule crista); 1 = connected to parastyle (by way of preparaconule crista); 2 = connected to paraconule (or near to it or to a virtual paraconule).

217. ML166*. M^1-2^ postparaconule crista: 0 = indistinct to absent; 1 = moderate; 2 = well-developed (connected to paracone).

218. ML168*. M^1-2^ hypometacrista: 0 = absent; 1 = weakly developed (low and short); 2 = well-developed (high).

219. ML169*. M^1-2^ hypoparacrista: 0 = absent; 1 = weakly developed (short); 2 = well-developed (high).

220. MLN*. Hypometaconulecrista (= metacrista or crista obliqua): 0 = indistinct to absent; 1 = moderate (not connected to protocone); 2 = well-developed (connected to protocone or postprotocrista).

#### **Cranial characters:**

221. Cr 1. Transverse septum arising from the cochlear housing: 0 = Absent; 1 = present and forming the lateral wall of an anterior accessory cavity pneumatized from the tympanic cavity; 2 = present and forming the lateral wall of an anterior accessory cavity pneumatized from the epitympanic recess.

222. Cr 2. Extent of pneumatization of anterior accessory cavity: 0 = Anterior accessory cavity lies anterior to the tympanic cavity and is not trabeculated; 1 = anterior accessory cavity extends medial to the tympanic cavity, and is trabeculated.

223. Cr 3*. Pneumatization of mastoid (from epitympanic recess?): 0 = absent; 1 = present (moderately inflated); 2 = 1 = present (greatly inflated).

224. Cr 4. Presence or absence of perbullar pathway: 0 = absent; 1 = present and formed exclusively by the petrosal bone.

225. Cr 5. Anteroposterior location of posterior carotid foramen in bulla: 0 = Posterior to line joining midpoints of tympanic bones; 1 = anterior to this line.

226. Cr 6*. Mediolateral position of posterior carotid foramen in bulla: 0 = medial; 1 = midline of the bulla; 2 = lateral.

227. Cr 7. Ventrodorsal position of the carotid foramen in the bulla: 0 = dorsal, adjacent to basioccipital or mastoid bone; 1 = ventral.

228. Cr 8*. Position of posterior carotid foramen relative to fenestra cochleae: 0 = posterior; 1 = ventral; 2 = anterior.

229. Cr 9. Position of the internal carotid canal relative to the fenestra cochleae: 0 = runs across ventral lip of the fenestra cochleae, shielding it from ventral view when a canal is present; 1 = internal carotid canal does not shield the fenestra cochleae from ventral view.

230. Cr 10. Position of the portion of the internal carotid/promontory artery (or its accompanying nerves) lying on the promontorium anterior to the fenestra cochleae: 0 = on ventrolateral surface of promontorium; 1 = contacting only the cupula of the cochlea.

231. Cr 11. Size of stapedial and promontory canals: 0 = both stapedial and promontory canals are large; 1 = stapedial slightly smaller than promontory; 2 = stapedial highly reduced or absent altogether; 3 = stapedial larger than promontory; 4 = both promontory and stapedial canals absent.

232. Cr 12. Morphology of promontory canal, when present: 0 = open trough; 1 = complete canal.

233. Cr 13. Presence or absence of canal for internal carotid artery or nerves: 0 = absent; 1 = present.

234. Cr 14. Position of ventral edge of the tympanic bone: 0 = intrabullar, or aphaneric; 1 = extrabullar or phaneric.

235. Cr 15. The shape of the tympanic bone: 0 = ribbon-like or only slightly expanded; 1 = laterally expanded into a collar or tube; ? = due to fusion with surrounding bones, of unknown shape.

236. Cr 16. Morphology of annular bridge: ? = This character is not analyzable in those taxa with an extrabullar tympanic, or those in which this region is not known; 0 = Linea semicircularis or partial anular bridge formed on a entotympanic bulla; 1 = linea semicircularis formed on a petrosal bulla; 2 = a complete annular bridge.

237. Cr 17. Encroachment of the auditory bulla on the pterygoid fossa: 0 = absent; 1 = present and formed by anterior accessory cavity; 2 = present and formed by the tympanic cavity.

238. Cr 18. Nature of contact between the lateral pterygoid plate and the bulla wall: 0 = absent; 1 = laminar; 2 = abutting.

239. Cr 19. Extent of contact between the lateral pterygoid plate and the bulla wall: 0 = slight; 1 = or very extensive.

240. Cr 20. Flange of basioccipital overlapping medial bulla wall: 0 = absent or minimal; 1 = extensive.

241. Cr 21. Suprameatal foramen: 0 = absent; 1 = present, small and in the posterior root of the zygomatic arch; 2 = present, large, and above the external auditory meatus.

242. Cr 22. Patent parotic fissure: 0 = present; 1 = absent.

243. Cr 23*. Size of orbits: 0 = small; 1 = large; 2 = extremely large.

244. Cr 24*. Postorbital closure: 0 = none; 1 = postorbital bar present; 2 = postorbital septum present.

245. Cr 25. Composition of the postorbital septum: 0 = zygomatic forms most of the septum; 1 = frontal forms most of the septum.

246. Cr 26. Zygomatic-lacrimal contact: 0 = present; 1 = absent.

247. Cr 27. Pronounced interorbital constriction: 0 = absent; 1 = present below olfactory tract.

248. Cr 28. Contact between lacrimal and palatine: 0 = present; 1 = separated by a large fronto-maxillary contact (and in some taxa, a small os planum of the ethmoid); 2 = separated by a large os planum.

249. Cr 29. Foramen rotundum: 0 = absent; 1 = present.

250. Cr 30. Position of lacrimal foramen: 0 = outside orbital margin; 1 = within the orbit or on the rim.

251. Cr 31. Metopic suture in adult: 0 = unfused; 1 = fused.

252. Cr 32. Orbital convergence: 0 = less convergent than primates; 1 = primate-like values for convergence.

253. Cr 33*. Posterior nasal spine: 0 = reduced or absent; 1 = small but distinct; 2 = robust and long

254. Cr 34. Posterior palatine torus: 0 = present; 1 = absent.

255. Cr 35. Pyramidal processes: 0 = medially placed; 1 = laterally placed.

256. Cr 36*. Length of medial pterygoid plate: 0 = long medial pterygoid plate extending one-third to one half of the distance to the anterior surface of the bulla; 1 = short but distinct from lateral pterygoid plate for its entire dorsoventral extent; 2 = medial pterygoid plate entirely absent, or reduced to a low rugosity.

257. Cr 37. Snout length: 0 = long snouts; 1 = short snouts.

258. Cr 38. Maxillary depth: 0 = deep; 1 = shallow.

259. Cr 39. Complete symphyseal fusion: 0 = absent; 1 = present.

260. Cr 40. Temporomandibular joint morphology: 0 = biconcave and transversely wide; 1 = anteroposteriorly oriented trough.

261. Cr 41. Entoglenoid process morphology: 0 = weak or absent; 1 = strong.

262. Cr 42. Inter-incisor diastema width: 0 = broad and wider than that of extant haplorhines; 1 = narrow, haplorhine-like.

263. Cr43. Coronoid height relative to condyle: 0 = very far above; 1 = slightly above or equal.

264. Cr44*. Condyle height relative to toothrow: 0 = at level of tooth row; 1 = slightly above; 2 = well above tooth row.

265. Cr45. Corpus robusticity: 0 = shallow; 1 = deep.

266. Cr46. Zygomatico-parietal contact at pterion: 0 = no postorbital closure; 1 = zygomatico-parietal contact; 2 = alisphenoid-frontal contact.

267. Cr47. Enclosure of intratympanic portion of facial nerve in a bony canal: 0 = no canal, facial runs in a sulcus; = bony canal present.

268. Cr48. Epitympanic crest: 0 = absent; 1 = present.

269. Cr49. Broad ascending wing of premaxilla: 0 = narrow; 1 = broad.

270. Cr 50/301. Basioccipital stem: 0 = narrow; 1 = broad.

271. Cr51/302. Choanal shape: 0 = narrow; 1 = broad.

272. Cr52/292. Orientation of the mandibular symphysis: 0 = symphysis procumbent; 1 = symphysis erect.

#### **Postcranial characters:**

#### Humerus

273. H1*. Shape of distal edge of the humeral trochlea: 0 = cylinder, distal edge perpendicular to shaft; 1 = distal edge somewhat angled to shaft; 2 = distal edge very angled.

274. H2. Relative heights of medial and lateral edges of humeral trochlea: 0 = subequal; 1 = medial edge more flared than lateral edge.

275. H3*. Trochleocapitular ridge: 0 = absent; 1 = weak but distinct; 2 = moderately distinct; 3 = very distinct.

276. H4. Waisted trochlea (Minimum trochlear diameter/maximum trochlear diameter x 100): 0 = > 70 (unwaisted); 1 = < 70 (waisted).

277. H5*. Width of capitulum relative to trochlea (100 x ventral capitulum width/ventral trochlear width): 0 = < 100; 1 = between 100 and 140; 2 = 140-200; 3 = greater than 200.

278. H6. Entepicondylar foramen: 0 = present; 1 = variable; 2 = absent .

279. H7. Entepicondylar foramen position: 0 = above medial epicondyle; 1 = above ventral trochlea; 2 = above dorsal trochlea.

280. H8. Medial epicondyle size: 0 = reduced; 1 = prominent.

281. H9. Dorsal placement of medial epicondyle: 0 = parallel ; 1 = slight dorsal; 2 = large dorsal angle.

282. H10’*. Shape of the lateral edge of the dorsal trochlea: 0 = not pronounced; 1 = moderately pronounced; 2 = very pronounced.

283. H10”*. Shape of the medial edge of the dorsal trochlea: 0 = not pronounced; 1 = moderately pronounced; 2 = very pronounced.

284. H11*. Dorsoepitrochlear fossa: 0 = present (strong); 1 = small, shallow; 2 = absent.

285. H12*. Olecranon fossa shape: 0 = shallow; 1 = moderate; 2 = deep.

286. H13. Supinator crest: 0 = prominent; 1 = low.

287. H14*. Brachialis flange; 0 = broad; 1 = moderate; 2 = narrow.

288. H15. Bicipital groove morphology: 0 = shallow; 1 = deep.

289. H16. Deltopectoral crest: 0 = prominent; 1 = low; 2 = flattened superiorly.

290. H17. Deltotriceps crest: 0 = low; 1 = prominent.

291. H18/. Capitular tail: 0 = ventral articular wdth <2.5 times the ventral capitular width; 1 = ventral articular wdth > 2.5 times the ventral capitular width.

292. H19/*. Ratio of humerus length to femur length (H/F): 0 = 100* H/F ≤ 65; 1 = H/F > 65, ≤ 80; 2 = H/F > 80.

#### Carpal bones

293. W1. Size of os centrale, orientation of centrale-trapezoid facet, and articulation with hamate: 0 = small os centrale, facet faces distally, no articulation with hamate; 1 = large centrale, facet faces distoradially, articulation with hamate.

294. W2. Ulnar-pisiform articulation: 0 = Facet on pisiform for ulnar styloid process is roughly equal in size to that for triquetrum; 1 = Facet on pisiform for ulnar styloid process is much enlarged and deeply excavated.

#### Os pelvis

295. OP1/299. Gluteal tuberosity: 0 = present; 1 = absent.

296. OP2/300. Position of posterior gluteal tuberosity: 0 = Proximal to or level with lesser trochanter; 1 = distal to lesser trochanter.

#### Femur

297. F1*. Length of femoral neck: 0 = < 75; 1 = 75-120; 2 = > 120.

298. F2*. Angle of femoral neck: 0 = < 60; 1 = 60-70; 2 = > 70.

299. F3. Angle of lesser trochanter: 0 = medial (0-30^o^); 1 = posterior (>30^o^)

300. F4*. Size of third trochanter: 0 = large; 1 = small; 2 = low crest or absent.

301. F5*. Knee index (Antero-posterior diameter of distal femur/ mediolateral diameter of distal femur ): 0 = < 90 (shallow knee); 1 = 90 – 100; 2 = > 100 (deep knee).

302. F6*. Femoral head shape: 0 = spherical; 1 = semicylindrical; 2 = cylindrical.

303. F7. Anterior extension of greater trochanter: 0 = no extension; 1 = extension present.

304. F8. Anterior bend of proximal femur: 0 = none; 1 = bent

305. F9*. Relative length of trochanteric fossa: 0 = long (> 125); 1 = moderate (110-125); 2 = very short (< 110).

306. F10. Presence of intertrochanteric crest: 0 = crest absent; 1 = crest present.

307. F11*. Size of lesser trochanter: 0 = large; 1 = intermediate; 2 = small.

308. F12. Lateral rim of knee: 0 = low; 1 = high.

#### Tibia

309. T1’. Fusion of tibia and fibula: 0 = absent; 1 = present.

310. T1”*. Articulation tibia/fibula: 0 = small; 1 = moderate; 2 = extensive.

311. T3. Shape of distal surface of tibia: 0 = square/parallel; 1 = triangular.

312. T4*. Rotation of the medial malleolus: 0 = none; 1 = slight; 2 = strong.

313. T5*. Shape of medial malleolar articular surface: 0 = flat; 1 = anteriorly convex, posteriorly flat; 2 = all convex.

314. T6. Shape of distal tibial shaft: 0 = no compression; 1 = anteroposteriorly compressed.

315. T7. Position of tibialis posterior groove: 0 = on medial side of malleolus; 1 = on posterior side of malleolus.

#### Talus

316. A1. Position of the flexor hallucis longus groove: 0 = lateral to trochlea; 1 = central to trochlea.

317. A2’*. Shape of talo-fibular facet: 0 = steep-sided; 1 = steep-sided with a platar lip; 2 = sloped obliquely.

318. A4’*. Development of the talar posterior trochlear shelf: 0 = none; 1 = weakly developed; 2 = well developed (prominent).

319. A5’. Talar neck length (NL/TL x 100): 0 = short (< 50); 1 = long (> 50).

320. A6. Medial talo-tibial facet: 0 = short (does not reach to plantar edge of bone); 1 = long.

321. A7/295. Lateral talar trochlear asymetry: 0 = absent; 1 = present.

322. A8/296. Talar cotylar fossa: 0 = shallow; 1 = deep, medially projecting.

323. A9’/297. Width of the head of the talus (HW/HHT x 100): 0 = < 120; 1 = > 120.

324. GEB1*. Talar neck angle: 0 = < 20°; 1 = 20-30°; 2 = > 30°.

325. GEB2*. Talar body height (HT/MTRW x 100): 0 = < 100; 1 = 100-120; 2 = 120-150.

326. GEB3*. TW/TL x 100: 0 = < 60; 1 = > 60.

#### Calcaneus

327. C1*. Anterior calcaneal elongation: 0 = not elongate (ACL or anterior calcaneal ratio < 40); 1 = moderate (ACL ≥ .40-45); 2 = long (> .45).

328. C2*. Position of the peroneal tubercle: 0 = distal to joint; 1 = at joint; 2 = proximal to joint.

329. C3. Posterior calcaneal bowing: 0 = absent; 1 = present.

330. C4/298. Calcaneo-cuboid articulation: 0 = articular wedge absent (fan-shaped); 1 = articular wedge present (more circular).

#### Navicular

331. N1*. Length relative to width: 0 = short (<90); 1 = moderate (100-150); 2 = long (>150).

332. N3. Morphology of the naviculocuboid articulation: 0 = cuboid facet on navicular contacts only the ectocuneiform; 1 = cuboid facet contacts the ectocuneiform and mesocuneiform facet.

#### Entocuneiform

333. E1*. Shape of Entocuneiform/MT1 articulation: 0 = dorsally reduced; 1 = dorsal moiety of joint enlarged relative to ventral moiety; 2 = dorsal moiety greatly enlarged.

334. E2. Lateral process of entocuneiform: 0 = small; 1 = hypertrophied.

#### General Foot

335. O1. Foot axis: 0 = mesaxonic; 1 = paraxonic; 2 = ectaxonic.

336. O2. Toilet claw: 0 = absent; 1 = present.

337. O3. Prehallux: 0 = present; 1 = absent.

338. O4. Metatarsus length: 0 = short; 1 = long.

#### Metatarsal

339. MT1*. Peroneal tubercle of MTI: 0 = very large; 1 = large; 2 = small.

340. MT2. Hallux length: 0 = short; 1 = long.

#### **Visual system:**

341. V1/288. Optic fovea: 0 = absent; 1 = present.

342. V2/290. Tapetum lucidum: 0 = present; 1 = absent.

#### **Miscellaneous other characters:**

343. 289. Haplorhini vs strepsirrhine: 0 = strepsirrhine; 1 = haplorhine.

#### **Molecular and physiological:**

344. MOL1/303. SINE (short interspersed nuclear elements) markers at the human locations 12p13-pter on chromosome 12: 0 = SINE absent; 1 = SINE present.

345. MOL2/304. SINE (short interspersed nuclear elements) markers at the human location 7q22, on chromosome 7: 0 = SINE absent; 1 = SINE present.

346. MOL4/291. Ability to synthesize Vitamin C: 0 = synthysis possible; 1 = synthysis not possible.

#### **Placentation:**

347. PL1/305. Placentation: 0 = Diffuse, epitheliochorial; 1 = Discoidal, hemochorial.

348. PL2/306. Blastocyst attachment: 0 = noninvasive; 1 = invasive.

349. PL3/307. Amniotic cavity: 0 = primordial cavity absent; 1 = primordeal cavity present.

350. PL4/308. Choriovitteline placenta: 0 = present; 1 = absent.

351. PL5/309. Embryonic body stalk: 0 = absent; 1 = present.

352. PL6/310. Allantois development: 0 = large, vesicular; 1 = rudimentary.

**Character sources:**

- For details about the source of most characters: Ross *et al.*, 1998, Kay *et al.*, 2004;
- PL1/305, PL2/306, PL3/307, PL4/308, PL5/309, PL6/310: Luckett, 1975; Luckett, 1976; Luckett & Hartenberger, 1985;
- MOL1/303, MOL2/304, MOL3/305: Schmitz & Zischler, 2004;
- MOL4/291: Pollock & Mullin, 1987;
- 289: Pocock, 1918;
- V1/288, V2/290: Martin, 1990;
- C4/298, A7/295, A8/296, A9/297, OP1/299, OP2/300, H18, H19, Cr50/301, Cr51/302: Seiffert *et al.*, 2004;
- Cr52/292: Beard & MacPhee, 1994;
- A1, A2’, A4, A5’, A6, GEB1-3: Gebo *et al.*, 2001;
- m8’, m9’ m8-9-10’, p4’, p4’’, p9’, p9’’, m11’, m20’, P18’, M7’, M12’, M16’, M17’, M17’’, M22’, ML18-19, ML88, ML126-127, ML147-169, MLN: Marivaux *et al.*, 2001 ; Marivaux et al., 2005 ; Marivaux in Tabuce et al., 2009 ; this work.

Beard KC, MacPhee RDE (1994) Cranial anatomy of *Shoshonius* and the antiquity of Anthropoidea. In: Fleagle JG, Kay RF, editors. Anthropoid Origins. New York: Plenum Press. pp. 55-97.

Gebo DL, Dagosto M, Beard KC, Qi T (2001) Middle Eocene primate tarsals from China: implications for haplorhine evolution. Am J Phys Anthropol 116: 83-107.

Kay RF, Williams BA, Ross CF, Takai M, Shigehara N (2004) Anthropoid origins: a phylogenetic analysis. In: Ross CF, Kay RF, editors. Anthropoid Origins: New Visions. New York: Kulver/Plenum. pp. 91-135.

Luckett WP (1975) Ontogeny of the fetal membranes and placenta: their bearing on primate phylogeny. In: Luckett WP, Szalay FS, editors. Phylogeny of the Primates. New York: Plenum Press. pp. 157-182.

Luckett WP, Hartenberger J-L, editors (1985) Evolutionary Relationships among Rodents. A Multidisciplinary Analysis. Plenum ed. New York, London: Nato. 721 p.

Marivaux L, Antoine P-O, Baqri SRH, Benammi M, Chaimanee Y, et al. (2005) Anthropoid primates from the Oligocene of Pakistan (Bugti Hills): data on early anthropoid evolution and biogeography. Proc Natl Acad Sci USA 102: 8436-8441.

Marivaux L, Welcomme J-L, Antoine P-O, Métais G, Baloch IM, et al. (2001) A fossil lemur from the Oligocene of Pakistan. Science 294: 587-591.

Martin RD (1990) Primate Origins and Evolution. A Phylogenetic Reconstruction; Martin RD, editor. London: Chapman and Hall. 804 p.

Pocock RI (1918) On the external characters of the lemurs and of *Tarsius*. Proc zool Soc Lond 1918: 19-53.

Pollock JI, Mullin RJ (1987) Vitamin C biosynthesis in prosimians: evidence for the anthropoid affinity of *Tarsius*. Am J Phys Anthropol 73: 65-70.

Ross C, Williams B, Kay RF (1998) Phylogenetic analysis of anthropoid relationships. J Hum Evol 35: 221-306.

Schmitz J, Zischler H (2004) Molecular cladistic markers and the infraordinal phylogenetic relationships of Primates. In: Ross CF, Kay RF, editors. Anthropoid Origins: News Visions. New York: Kluwer/Plenum. pp. 65-77.

Seiffert ER, Simons EL, Simons CVM (2004) Phylogenetic, biogeographic, and adaptive implications of new fossil evidence bearing on crown anthropoid origins and early stem catarrhine evolution. In: Ross CF, Kay RF, editors. Anthropoid Origins: New Visions. New York: Kulver/Plenum. pp. 157-181.

Tabuce R, Marivaux L, Lebrun R, Adaci M, Bensalah M, et al. (2009) Anthropoid *vs.* strepsirhine status of the African Eocene primates *Algeripithecus* and *Azibius*: craniodental evidence. Proceedings of the Royal Society of London, B 276: 4087-4094.
