# Supplementary material for: Djebelemur, a Tiny Pre-Tooth-Combed Primate from the Eocene of Tunisia: A Glimpse into the Origin of Crown Strepsirhines
Source: PLoS One. 2013 Dec 4;8(12):e80778. doi: 10.1371/journal.pone.0080778 (PMC3851781; doi:10.1371/journal.pone.0080778)
Supplement: Dataset S2 — Taxa (genera and species) selected for the phylogenetic analyses. (DOC) [file pone.0080778.s002.doc]

**Dataset S2**

**Taxa (genera and species) selected for the phylogenetic analyses.**

| 1. Scandentia |
| --- |
| 2. *Paromomys maturus - P. depressidens* |
| 3. *Plesiadapis tricuspidens - P. jepi* |
| 4. *Plesiolestes problematicus* |
| 5. *Ignacius frugivorous - I. fremontensis* |
| 6. *Purgatorius unio* |
| 7. *Notharctus robustor - N. nunienus - N.* sp. |
| 8. *Cantius eppsi* |
| 9. *Pelycodus jarrovii* |
| 10. *Pronycticebus gaudryi* |
| 11. *Protoadapis curvicuspidens* |
| 12. *Periconodon jaegeri* |
| 13. *Leptadapis magnus* |
| 14. *Adapis parisiensis* |
| 15. *Anchomomys gaillardi* |
| 16. *Anchomomys frontanyensis* |
| 17. *Donrussellia gallica - D.* sp. |
| 18. *Mahgarita stevensi* |
| 19. *Aframonius dieides* |
| 20. *Afradapis longicristatus* |
| 21. *Asiadapis cambayensis* |
| 22. *Rencunius zhoui* |
| 23. *Hoanghonius stehlini* |
| 24. *Guangxilemur singsilai* |
| 25. *Sivaladapis nagrii* |
| 26. *Plesiopithecus teras* |
| 27. *Azibius trerki* |
| 28. *Algeripithecus minutus* |
| 29. "*Anchomomys*" *milleri* |
| 30. *Djebelemur martinezi* |
| 31. *Wadilemur elegans* |
| 32. *Karanisia clarki* |
| 33. *Saharagalago misrensis* |
| 34. *Galago senegalensis* |
| 35. *Galagoides demidoff* |
| 36. *Otolemur crassicaudatus* |
| 37. *Arctocebus calabarensis* |
| 38. *Perodicticus potto* |
| 39. *Nycticebus coucang* |
| 40. *Loris tardigradus* |
| 41. *Lemur catta* |
| 42. *Eulemur fulvus* |
| 43. *Varecia variegata* |
| 44. *Lepilemur ruficaudatus* |
| 45. *Hapalemur griseus* |
| 46. *Propithecus verreauxi* |
| 47. *Indri indri* |
| 48. *Avahi laniger* |
| 49. *Phaner furcifer* |
| 50. *Microcebus murinus* |
| 51. *Cheirogaleus major* |
| 52. *Mirza coquereli* |
| 53. *Allocebus trichotis* |
| 54. *Altanius orlovi* |
| 55. *Absarokius abotti - A.* sp. |
| 56. *Anaptomorphus westi - A.* sp. |
| 57. *Anemorhysis savagei - A. tenuiculus* |
| 58. *Arapaphovius gazini* |
| 59. *Dyseolemur pacificus* |
| 60. *Hemiacodon gracilis* |
| 61. *Loveina zephyri* |
| 62. *Macrotarsius montanus* |
| 63. *Microchoerus erinaceus* |
| 64. *Nannopithex pollicaris - N. zuccolae - N.* sp. |
| 65. *Necrolemur antiquus* |
| 66. *Omomys carteri - O.* sp. |
| 67. *Pseudoloris parvulus* |
| 68. *Shoshonius cooperi* |
| 69. *Steinius vespertinus* |
| 70. *Strigorhysis bridgerensis - S.* sp. |
| 71. *Teilhardina americana* |
| 72. *Teilhardina belgica* |
| 73. *Tetonius homunculus - T.* sp. |
| 74. *Uintanius ameghini* |
| 75. *Washakius insignis - W. woodringi* |
| 76. *Tarsius spectrum* - *T.* sp. |
| 77. *Xanthorhysis tabrumi* |
| 78. *Afrotarsius chatrathi* |
| 79. *Afrasia djijidae* |
| 80. *Eosimias sinensis - E. centennicus - E.* sp. |
| 81. *Bahinia pondaungensis* |
| 82. *Phenacopithecus krishtalkai - P. xueshii* |
| 83. *Phileosimias kamali - P. brahuiorum* |
| 84. *Proteopithecus sylviae* |
| 85. *Biretia fayumensis - B. megalopsis* |
| 86. *Serapia eocaena* |
| 87. *Simonsius grangeri* |
| 88. *Qatrania wingi* |
| 89. *Apidium phiomense* |
| 90. *Parapithecus fraasi* |
| 91. *Arsinoea kallamos* |
| 92. *Oligopithecus savagei - O. rogeri* |
| 93. *Catopithecus browni* |
| 94. *Aegyptopithecus zeuxis* |
| 95. *Moeripithecus markgrafi* |
| 96. *Siamopithecus eocaenus* |
| 97. *Pondaungia cotteri* (+ "*A. mogaungensis*") |
| 98. *Ganlea megacanina* |
| 99. *Myanmarpithecus yarshensis* |
| 100. *Bugtipithecus inexpectens* |
| 101. *Branisella boliviana* |
| 102. *Dolichocebus gaimanensis* |
| 103. *Neosaimiri fieldsi* |
| 104. *Saimiri sciureus* |
| 105. *Callicebus moloch* |
| 106. *Aotus trivirgatus - A. lemurinus* |
