# Supplementary material for: Djebelemur, a Tiny Pre-Tooth-Combed Primate from the Eocene of Tunisia: A Glimpse into the Origin of Crown Strepsirhines
Source: PLoS One. 2013 Dec 4;8(12):e80778. doi: 10.1371/journal.pone.0080778 (PMC3851781; doi:10.1371/journal.pone.0080778)
Supplement: Dataset S3 — Matrix of the phylogenetic analyses. (RTF) [file pone.0080778.s003.rtf]

Dataset S3

Matrix of the phylogenetic analyses.

In order to replicate the phylogenetic results presented in the paper, the matrix (below) should be copied, pasted and saved as a new text document with extension '*.nex'. The matrix should then be loaded within PAUP*. 

In order to replicate the phylogenetic results presented in the paper using a molecular scaffold, the constraint tree (Dataset_S4-Molecular-Scaffold) should be copied, pasted and saved as a new text document with the same name as the matrix but with extension '*.tre'. The matrix should then be loaded within PAUP* but the brackets ( [ ] ) of the following command line texts must be removed: [loadconstr asbackbone=yes;] and [enforce=yes;]

After removing the brackets of these command line texts, the backbone constraint will load automatically when you will execute the matrix file by PAUP*.


#NEXUS

[ File saved by NDE version 0.5.0, Fri Mar 22 09:42:32 2013 ]

BEGIN DATA;

DIMENSIONS  NTAX=106 NCHAR=352;

FORMAT DATATYPE=STANDARD MISSING=? GAP=- SYMBOLS="012345";
	
	
	MATRIX
		Scandentia           00???????0 22?1????00 00?2111000 1120100200 0010001101 ?0000020?? 100??00111 1022200000 1000012200 0130112021 1000221100 10222??101 0{01}020?012? ???????0?0 0122002001 0010201022 00?0000020 001100000- 0?00-20000 0101111111 1(01)20000220 0001220000 0?00000010 0110000000 100(01)?00000 1000000100 0001001111 0010011001 1112001000 1?0001?100 0010000000 0000010000 1011000000 0000010120 0000000000 00
		Paromomys            ?????????? ?????????? ????001011 1021101210 011?20?001 01?1?21001 00?5000111 1112220011 2000011201 1132330?11 1000221200 11(12)2112111 1102?????? ?????????? ?122?1?211 11??{12}00000 0000000120 1022-00010 0?00-20010 1101021111 2011110221 1221111001 ?????????? ?????????? ?????????? ?????????? ????0????? ?????????? ?????????? ?????????? ?????????? ?????????? ?????????? ?????????? ?????????? ??
		Plesiadapis          111?24??2? 2101?02200 ???1--1001 1022--0??0 00111?2??1 00?110100? -231100111 2122210001 2000012200 0131120112 1300222200 1112202110 0101?????? ?????????? ?222012100 1110000110 0010000020 1022-00010 2100-20010 010112011? 2001100221 1221110001 0??0020000 4010020??0 0000??0100 00100?0?00 110000001? ?010112001 0110000000 ??????1000 0000200000 0000010000 ?01100000? ??01??0??? ?????????? ??
		Plesiolestes         1?1014?020 2101000200 0001001001 102110220? 1212201101 02?0?????? 1?????0111 2122200001 2000011200 0132220011 1300222100 1122203100 0102?????? ?????????? ???2?2?01? 11?0100111 0??0000120 1022-00010 0?00-20210 010121011? 1010001221 1221110002 ?????????? ?????????? ?????????? ????????0? ????0????? ?0???????? ?????????? ?????????? ?????????? ?????????? ?????????? ?????????? 0????????? ??
		Ignacius             ????????20 2201???2?? ??????11?1 1022--1??? 0011201100 02?102001? -004000111 1222220011 2000012200 0132230122 2220221200 1012202111 1102?????? ?????????? ???2?1?21? 11??1010?? 0?10?00120 1022-00010 0?00-210?0 0?011?1111 2101110221 1221111002 0??0?20000 40?1100000 2000?10?01 0000000?00 1????????? ?????????? ?????????? ?????????? ?????????? ?????????? ?????????? ?????????? 0????????? ??
		Purgatorius          0????????? ????????0? ?????00011 1020102200 0011101101 0000020000 1015100111 1122200000 1000012200 0132110122 2333222200 1012223000 0102?????? ?????????? ???2?1?00? 11?02000?? 0??0?0?020 1011100020 0?00-{12}12?0 ???1??1111 1020001110 0110111000 ?????????? ?????????? ?????????? ????????0? ?????????? ?????????? ?????????? ?????????? ?????????? ?????????? ?????????? ?????????? ?????????? ??
		Notharctus           1001010000 110010102? 0?02000011 1021101210 0211101100 0100010110 1224100111 2022221011 1100113300 0132220211 1311222110 1{12}(12)2202111 0101?1???0 ?{01}0??????0 ?022{01}12000 0110201020 0010000122 1022-00010 100(12)(01)21011 0101{01}12111 2001100221 1331110001 0??0020000 301??????0 ?111?????? ????????00 0?010?111? ?01?30201? 0?????1??? ?0????1100 1000001100 0220102201 001020110? 11??0?1?1? 00???????? ??
		Cantius              ?????????? ?????????? ???????011 1021102?1? 0211101100 01?0020110 0234200111 2022210011 1000012200 0132220211 1301222200 1122203111 0101?????? ?????????? ??22?1?000 01?0201020 0010000120 1022-00010 0?01121011 0101011{01}01 2001100111 1330110001 ?????????? ?????????? ?????????? ????????0? ????0????? ?????????? ?????????? ?????????? ?????????? ?????02201 ??1020???? ?????????? ?????????? ??
		Pelycodus            ?????????? ?????????? ???????011 1021101?2? 0210101101 00?1010010 0224000111 2022221011 1100113300 0132220112 1311222120 1112102111 02{01}1?????? ?????????? ?????????? ?????????? ?????00122 1022-00010 2002(01)21010 0101{01}12111 2001100221 133111{01}001 ?????????? ?????????? ?????????? ?????????? ?????????? ?????????? ?????????? ?????????? ?????????? ?????????? ?????????? ?????????? ?????????? ??
		Pronycticebus        1???010?0? 000010??1? ????000011 1021101?1? 0110201101 01?0020100 0?24300111 212---0000 0000010100 0132330?11 1201222100 1012202111 0101?????? ???????0?? ?222?0?000 01??200112 0000000120 1001100010 0?00-210?1 1211121111 200100100? ?331111002 0?00020000 311001{12}101 0111?00110 01??1???00 ??12101111 0????????? ?????????? ?????????? ?????????? ?????????? ?????????? ?????????? 00???????? ??
		Protoadapis          1???01???? ????????1? 00?2000011 1022--2200 0111101100 10?0020101 -3(23)3500111 2111-00010 100001(01)100 0132331?12 1311222100 1112202111 0101?????? ?????????? ??22?1?00? 01??2100?? 001000?220 0011{01}0000- 0?020(12)0000 0201011100 2001100220 000111000(01) ?????????? ?????????? ?????????? ????????0? ?????????? ?????????? ?????????? ?????????? ?????????? ?????????? ?????????? ????1????? ?????????? ??
		Periconodon          ?????????? ?????????? ?????????? ???1001?-? ?01?1-?-00 00?002?100 0??51?0111 2(12)1---1000 1{12}00110100 0132{23}{23}1211 1111222110 11222021(01)1 0101?????? ?????????? ?????????? ????????1? ?????00120 101(01)01001? 0?2(12)111010 020112010? 2001100111 13(23)1110002 ?????????? ?????????? ?????????? ?????????? ?????????? ?????????? ?????????? ?????????? ?????????? ?????????? ?????????? ?????????? ?????????? ??
		Leptadapis           1??0?0??00 00001?201? 1102000011 1021002200 0210101100 0220010100 0234400111 110---0020 1020010100 0121320212 1211222000 1022203011 0210?????? ?0??????0? ?122022210 1110200100 1010000220 000110000- 0?00-20010 0211121100 20012011(01)1 1331110000 0?10020000 30?0010??0 0101?00??0 ?1??12??10 0???1?111? ?010311011 1121000000 ?????????? ?????????? ?????02201 00122112?? ??00??1?2? 00???????? ??
		Adapis               1001010000 000010201? 0122000011 1011002210 0210201100 022?031100 0214300111 110---0020 1010010100 0131330?11 1{23}11222000 1112203110 011010?0?2 20?2??100? ?122112201 1110110100 1010000220 000110000- 0?01120010 0201121100 2002201101 1331110000 0?10020000 (13)010010200 0101?001?0 (01)110121010 0?01101111 0010112011 0001000000 0?01001000 0000101100 1220102201 0112111101 ??00??1?2? 00???????? ??
		'Anchomomys g.'      ?????????? ?????????? ?????????? ???2--1??? ?01?0???01 00??01?1?? -??52?0111 121---0000 1010010100 0132220112 2332222100 1112223111 1121?????? ?????????? ?????????? ?????????? ?????00220 0022-0010- 0?01120010 02011211{01}1 2011000111 1330110102 ?????????? ?????????? ?????????? ????????0? ??020????? ?0???????? ?????????? ?????????? ?????????? ?????????? ?????????? ?????????? ?????????? ??
		'Anchomomys f.'      ?????????? ????????2? 00?20?0011 1?220012-0 00100-2-01 002101?11- 0?253?0111 221---0000 1010010100 0132{23}{23}0112 1321222200 1112223111 1111?????? ???????00? 1122113101 0110?01101 10(01)00002{12}0 10211000{01}0 0-01120010 02011{12}110{01} 2011110111 1331110102 ?????????? ?????????? ?????????? ?????????? ?????????? ?????????? ?????????? ?????????? ?????????? ?????????? ?????????? ?????????? ?????????? ??
		Donrussellia         ?1?1?????? ??0??????? ??????0011 1022001?1? 011?10?101 02?00(12)0120 01?5300111 1122100000 100001{12}200 0132220211 13(12)1222100 1(01)122(01)(23)101 0101?????? ?????????? ???2?1?000 01?0211011 0010000120 {01}022-000(01)0 0?011(12)1000 020112010? 2001111100 ?331110001 ?????????? ?????????? ?????????? ????????0? ??000??1?? ?????????? ?????????? ?????????? ?????????? ?????????? ?????????? ?????????? ?0???????? ??
		Mahgarita            1?0100???? 1?00???02? 0002001001 1022--12?0 00101?1101 00?0010111 -335200111 11----0000 1010010000 0132??1?1? 1?22222200 1012213?10 0011?????? ???????021 1022013100 0110111000 1010000220 1000100010 0?02021000 02111100?? 2002101221 0111110001 0?10020000 (12)110010110 0101?10??0 ??10121010 0?011011?? ?0???????? ?????????? ?????????? ?????????? ?????????? ?????????? ?????????? 00???????? ??
		Aframonius           10?10??0?? 000????02? 0002001001 1022002300 011110?10? 0000020011 032(34)100111 12----0000 1010010000 0131{23}{23}0111 1{23}{23}1222000 1002213110 0111?????? ????????2? ?022013101 0010201010 0010000220 1010101010 0?01120010 0211121101 2002110110 1331110001 ?????????? ?????????? ?????????? ????????1? ????1????? ?0???????? ?????????? ?????????? ?????????? ?????????? ?????????? ?????????? ?????????? ??
		Afradapis            100?110010 010010112? 0?020011-1 1022002-1- 011{01}101100 01-0020110 0335510111 11----0000 1(01)10010000 0131330-12 1222021000 1012213110 02111??100 00?101?031 102200-100 00--101110 0010000220 000022000- 2002(01)20010 0211111111 2002200220 0111110002 ?????????? ?????????? ?????????? ????????1? ????1????? ?0???????? ?????????? ?????????? ?????????? ?????021{01}1 001210???? ?????????? ?????????? ??
		Asiadapis            ?????????? ????????2? 0????01001 102{12}101?0? 0111102101 01?0010111 0235200111 111(12)?20000 1000011200 1132120211 {01}211212200 1111(01)231(01)1 010{12}?????? ?????????? ??22?2?000 00??201011 00{01}0000120 001210000- 0-0{12}100001 0201111101 2001100111 1331111{01}02 ?????????? ?????????? ?????????? ?????????? ????0????? ?????????? ?????????? ?????????? ?????????? ?????????? ?????????? ?????????? ?????????? ??
		Rencunius            ?????????? ?????????? ?????????? 1??2????1? ??1?0????0 10?001?100 ???52??11? 1?21?0001? ?010011200 013100?{01}12 130?21?210 11221?3101 1101?????? ?????????? ???2?1?10? 00??1?11?0 1?10?0???0 101?00?020 00230200?0 ???10?1200 10111010?0 ?3?12?0002 ?????????? ?????????? ?????????? ?????????? ?????????? ?????????? ?????????? ?????????? ?????????? ?????????? ?????????? ?????????? ?????????? ??
		Hoanghonius          ?????????? ????????2? 0??3001011 1022--1210 0010101101 110001000? -225200111 112---0010 1010010200 0131000021 1111222100 1122223101 11{01}1?????? ?????????? ?????????? ????????11 ?????0?1?0 10?110?010 10230200?0 ???10?1??1 2011110?0? 1?31?20002 ?????????? ?????????? ?????????? ?????????? ????0????? ?????????? ?????????? ?????????? ?????????? ?????????? ?????????? ?????????? ?????????? ??
		Guangxilemur         ?????????? ?????????? ?????????1 ??2?--1??? 0??0??1?01 1????????? -?????011? 1??---?01? ?010010?01 0?3100?021 100?22?100 11111?31(01)1 1102?????? ?????????? ???2?1?2?? 11?11000?0 1?10?0?120 001110??0- 00130202?0 0??11?1200 1012210?0? 1?31110002 ?????????? ?????????? ?????????? ?????????? ?????????? ?????????? ?????????? ?????????? ?????????? ?????????? ?????????? ?????????? ?????????? ??
		Sivaladapis          100???0?00 1000??103? 0003001011 1021102110 0210201100 0200030001 1?04500111 122---0020 1010010100 0131000021 1311222010 1112223100 0102?????? ?????????? ???2?2?201 11?0201011 1010000220 1022-00010 2003020211 010112020? 0012200001 1331110001 ?????????? ?????????? ?????????? ????????1? ????0????? ?0???????? ?????????? ?????????? ?????????? ?????????? ?????????? ?????????? ?????????? ??
		Plesiopithecus       3????????? ??0??????? 21?2110001 12220000-0 00111-2101 012102211- 0224120111 120---1120 1010110100 0132330-2{01} 0112221200 1120031012 1211?????? ???????000 01210?1101 0010?00111 0000000110 1022-000{01}0 0-020100?0 010010110? 2011101{12}{12}1 122111000{12} ?????????? ???????10? ??11-????0 1????11000 ??011????? ?0???????? ?????????? ?????????? ?????????? ?????????? ?????????? ?????????? ?????????? ??
		Azibius              ?????????? ?????????? ?????????1 11?2--0?2? 02100-2-01 00?211?10- -3?4300111 10----0100 1010010000 0132330-{01}2 1321221000 1122213010 1210?????? ?????????? ?02202?20(01) 1010100000 0011(01)00120 00000(01)000- 0-020(12)0000 110111000- 200110100- ---011000{12} ?????????? ?????????? ??1??????? ??????11?? ????0????? ?????????? ?????????? ?????????? ?????????? ?????02211 001010???? ?????????? ?????????? ??
		Algeripithecus       ?????????? ?????????? ?????1?001 1122000?2? 12100---01 00?211110- -225400111 110---(01)1(01)0 1010010100 0132330-{01}2 2331221(01)00 112221301{01} 1220?????? ?????????? ?022?22200 1010210(01)00 011(01)000120 10000000(01)0 0-{01}(12)(01)(12)0000 11(01)111010- 200110100- ---011000{12} ?????????? ?????????? ?????????? ????????0? ????0????? ?0???????? ?????????? ?????????? ?????????? ?????????? ?????????? ?????????? ?????????? ??
		'"Anchomomys" m.'    ?????????? ????????1? 0002001001 1122--02-0 00100-1101 0100021110 -23500011? 1?00?0111? ?010111100 013233?-22 232?21?220 11222?3111 1220?????? ?????????? ?????????? ?????????? ?????????0 ?????????? ?????????? ?????????? ?????????? ?????????? ?????????? ?????????? ?????????? ????????0? ????0????? ?0???????? ?????????? ?????????? ?????????? ?????????? ?????????? ?????????? ?????????? ??
		Djebelemur           ?????????? ????????1? 2????11001 1122--1?-? 00101-2-01 01?0011{01}1- -235000111 11----1110 1010110000 0132330-12 1311221200 1012123112 1221?????? ???????0?? ?022?1?000 00??201101 0010000220 0022-0000- 0-0{12}110000 0101111101 2021101111 1330110102 1?00?{12}?000 30???????? ??1??????? ??????110? ????0????? ?0???????? ?????????? ?????????? ?????????? ?????02211 0010(12)0???? ?????????? ?????????? ??
		Wadilemur            1??1?????? 1???????0? 2?-0?11001 11220020-0 0010101100 0200022001 0235010111 10----1100 1000110000 0132330-1{12} 1111222110 1122123111 1121?????? ?????????? ???2?1?20? 00??2011?2 0?11?0?120 000000?00- 0-00-200?1 0??12?2201 2021111111 1331110{01}02 ?????????? ?????????? ?????????? ????????0? ????0????? ?0???????? ?????????? ??????1100 ?21?001??? ?????????? ?????????? ?????????? ?????????? ??
		Karanisia            ???1?????? 1???????0? 20-0111001 1??2--0?-? ?01?0-?-01 01?000??10 -?????0111 1100?01110 1000111100 0132{23}30111 1111222210 1021103112 1120?????? ?????????? ??22?0?100 00??201111 1010000210 001100000- 0-02020000 01111211{01}1 2111110211 1331110(01)02 ?????????? ?????????? ?????????? ?????????? ????0????? ?????????? ?????????? ?????????? ?????????? ?????????? ?????????? ?????????? ?????????? ??
		Saharagalago         ?????????? ?????????? ?????????? ?????????? ?????????? ?????????? ???????1?? ??-???1?1? ?02011?000 ?132{23}??112 ?3??22?211 1122??3111 1?20?????? ?????????? ?????????? ?????????? ?????0???0 000?01?00- 0-011100?0 ???12?1101 20211101?1 ?3?11?0112 ?????????? ?????????? ?????????? ?????????? ?????????? ?????????? ?????????? ?????????? ?????????? ?????????? ?????????? ?????????? ?????????? ??
		'Galago s.'          100102?120 1211101000 20-0211001 1122--2010 0210202100 0101031000 -235120111 10----0000 1000110000 0131330-21 1000211100 000112210{01} 1110010000 01??000010 0122122200 1110101112 0002000120 10000{01}1100 0-00-00211 0101211101 1221100110 0330220002 2?10?000?? 4-011-0200 0111-{01}12?0 01?0120100 000100?00? 0010???0?1 0112000?0? ?010??0{12}01 22?0200?01 {01}{12}10002?(01)1 ??0(01)20220? 2100211001 0000000000 00
		Galagoides           100102?120 1211101000 20-0211001 1122--2010 0210202100 0201031000 -235120111 10----1110 1010110000 0132330-21 1000222200 1101123111 1111111000 010?000010 0122122210 1110100012 0002000120 000001010- 0-00-00211 0101211101 1221100220 0330220002 2?2000000? 4-011-0100 0111-{01}1200 0101110100 0001001001 0010201011 0112000100 0{01}11000{12}02 220020{01}100 {01}{12}10002101 0101101200 2100211001 00000????? ??
		Otolemur             100102?120 121110?000 20-0211001 1222002100 0110202100 0101021001 0235120111 10----1110 1000110000 0132330-21 1122222200 1101102111 11{12}1010000 01??000010 0122212211 1110101112 0002000120 000001000- 0-0{01}100010 110{01}201100 2221100111 1331110102 2?20?0000? 4-011-00-0 0111-{01}1200 0??0110100 000110?00? 0010???0?1 {01}112000?0? ?{01}1???0{12}0{01} 22?0{12}00?00 1{12}{01}0102211 00(01)120220? 2?00211001 00000????0 ?0
		Arctocebus           100102?120 1211102000 20-0211000 1222--2110 0210202100 0201032100 -203120111 21----0000 1000110000 0132331-21 1222222{12}00 1022211100 1111111000 010?100010 {01}122313210 1110201001 1012200220 000100000- 0-02020{01}00 010{01}112111 2210000220 0331110002 2?20?000?? 4-011-00-0 0101-10200 0??1110100 000100?00? 0010???2-0 1112{12}12?1? ?2?-??0{01}02 00?1{12}0??00 1220102?01 ??1(12)21{01}21? ????211021 00000????0 ?0
		Perodicticus         100102?120 1111101000 20-0111001 1022--01-0 00100-2101 -001000000 -230120111 100---111- 0011110100 0122333-00 0222222200 0{12}01032{01}01 1121010000 010?000010 0022313101 0010201001 0000000120 001100000- 0-0{01}100000 100{01}200001 2{01}11100111 1331110002 2?10?011?? 4-011-0200 0101-{01}0200 0??1110100 000110?00? 0010?1?2-{01} 011{12}00{01}?0? ?21-??0{01}01 00?0200?00 1220002111 0012(12)1020? 0?00211021 00000????0 ?0
		Nycticebus           100102?120 1111100000 20-0111001 1122--00?0 00100-1101 000100001? -223120111 100---1100 1010110100 0130330-11 1222222200 1101112001 1021010201 110?000010 0022203210 0010201101 1000000110 000001000- 0-01120001 1101201111 2221100111 1331110002 2?100010?0 4-011-00-0 0111-{01}1200 {01}211121100 000100100? 0010012020 0112112011 ?21-??0{01}0{12} 0000200100 12201021(01)1 101211{01}21? 1100211021 00000?0000 00
		Loris                100102?120 1111100000 20-0111001 1122--1010 0210101101 0110020000 -225020111 11----1110 1010110001 1130330-11 2131211100 1112213110 1020111000 000?000010 0122202200 0010101122 0001000220 000001000- 0-00-00010 0221211101 2221101001 1330110002 2?10?0101? 4-011-00-0 0111-11200 0??0110100 100100?00? 0010???0?0 0112212?1? ?{12}1-??{01}{01}02 00?0200?00 1220102101 101201{12}21? 010?211021 00000????0 ?0
		Lemur                100102?120 2211101000 20-0221001 1022--1010 0110001101 0101020021 -105300111 220---102- 0020110100 0132333-11 1000222100 1101123010 1120010001 010?000010 0122012001 0010200101 0000000210 0021100-0- 0-23000001 0100110100 1011101001 1321110002 0?00020000 3010010100 0101-00010 1100110100 0001001111 00203010(12)1 0002001000 0{01}110?{12}200 2000001100 {01}220102201 011(12)21120? 1100211011 0000000000 00
		Eulemur              100102?120 2211101000 20-0221001 1022101010 0111100001 1101020011 0205310111 100---112- 0020{01}{01}0100 0112333-11 1000222200 1101133{01}11 12{12}1011001 110?000010 0122012000 00102001(01)0 0000000210 0012000-0- 0-20-00001 1200110100 {01}01{12}{12}10121 133100000{12} 0?00?2?00? 3010010100 0101-00010 1??0110100 000100?11? 0020?0?0?1 0002001?0? ?{01}11??1{12}00 20?0001?00 {01}220102201 01(01)(12)(12){01}120? 1100211011 0000000000 00
		Varecia              100102?120 2211101000 20-0221001 1022--1010 0110101101 0101030021 -205210111 11----112- 0021100000 0112333-11 1000222200 11(01)0033002 1221010001 110?000010 012201200{01} 0010201111 0000000220 0022-00-0- 0-03000001 0100010100 2012210111 13(23)0000000 0?00?2100? 3010010200 0101-00?10 {01}??0100100 000100?10? 0020?0?0?1 111200{01}?0? ?110??{12}{01}0{12} 20?0001?00 1220102201 01122112?? 0?0?211011 ??000????? ??
		Lepilemur            100102?120 2211101000 20-0221001 1122--1010 0210001101 1100010111 -233120111 11----0020 1010110000 0120330-11 1211222220 1021123102 1320?????? ???????011 0122212000 0010201111 0000000220 0022-00-0- 0-00-10001 0200221101 2012211221 1221110000 0?00?1100? 3010010200 0101-{01}0100 0??01{01}01{01}0 000100?10? 0000?0?0?{01} 0112000?0? ?010??120{01} 20?0001?00 12{12}01022(01)1 00111(01){12}0?? 1???211011 00000????? ?0
		Hapalemur            100102?120 2211101000 20-0221001 1120202000 0210201100 0201020021 2205410111 1100-000(02)- 0(01)10011100 0132333-21 0111222000 1111123110 1221011000 010?000010 012202021{01} 1110201110 0000000220 0022-00-0- 0-00-10001 0200211100 20{01}2211001 1330110000 ??0??????? ????0?0000 0101-00??0 1???111000 000010???? 00???0?0?1 ?????0??0? ?0????2100 20??001?0? ???0?02201 111{12}21???? ?????1???? ??000????? ??
		Propithecus          2?01---120 2221101000 20-02211-0 0222--2--- 00100-2100 01-1021021 -335020111 202---1011 0020010210 0130332-21 1220222000 1101123110 0321110211 111?200030 0012-0-001 00--201100 0010000220 1000120010 2000-20000 0102211111 2002200221 133111000{12} 0?00?2100? 3010010200 0101-00100 1??1111001 001210?10? 0000?0?0?1 011200{01}?0? ?{01}10??{12}{12}00 20?0101?00 0100102201 00(01){12}1{01}120? 0100211011 00000????0 ?0
		Indri                2?01---120 2221100010 20-02211-0 1222--2--- 00100-2-01 01-101002- -335120111 201---01(01)0 00200102(01)0 2130332-22 1310222{01}00 1102113100 0311110001 110?000030 0012-0-001 0---201-00 0000000220 000012000- 1000-10000 0102221111 2002{12}00120 0331110012 ??0??????? ????0?0000 0101-00??0 1???110001 ?01210???? 00???1?0?1 ?????1??0? ?0????1200 10??101?0? ???0?02201 00021{01}???? ?????1???? ??000????? ??
		Avahi                2?01---120 2221101000 20-02211-0 1221202-1- 0210002100 00-1022011 -335020111 202---{01}110 11200102(01)0 1130332-21 1220222000 1002223110 0{23}11010200 110?000030 1011-0-001 0---201100 0010000210 1000120010 2000-20100 0202{12}02211 2002200220 0331110002 ??0??????? ????0?0000 0111-00??0 1???111101 ?01210???? 00???0?0?1 ?????0??0? ?0????1{12}01 20??001?0? ???0?022(01)1 00121{01}???? ?????1???? ??000????? ??
		Phaner               100112?120 2211?00000 20-0221000 1022--00-0 00100-2-01 10000{01}0110 -225020111 21----111- 0010110000 1122333-11 1100222200 11{12}1120112 112{01}010211 110?100010 0002302001 0010201100 0010000220 001100000- 0-0{01}120000 0101211101 2011100121 0311110102 0?00?????? ???0010100 0111-00??0 0???110100 ?00000???? 00???0?0?1 ?????0??0? ?0????2201 20??001?0? ???0?022(01)1 00022(01)???? ?????1???? ??000????? ??
		Microcebus           100102?120 2211101000 20-0221000 1122--00-0 00100-1-00 102101111- -234120111 11----111(01) 1010110000 0132330-11 1000222200 1112203101 1121011201 010?210010 1022013001 0010200100 1010000120 0011000-0- 0-(01)3010000 0210(12)1000{01} 2010010111 1331000202 0?000{12}0000 4-00010100 0111-01200 0100110100 000100101? 0000300021 00020000{01}1 ?111??{12}{12}01 {12}001001101 11201022(01)(01) 001(01)10220? 2100211011 0000000000 00
		Cheirogaleus         100102?120 2211101000 20-0221000 1122--2010 0110202100 0201031(01)21 -305010111 21----1120 10(12)1110000 0112331-10 0000222200 111002211(12) 1(12)21011211 010?200010 0022212201 1010201001 1000000120 0022-00-0- 0-03010000 0200111100 20(01)2210221 1221110{01}01 0?00?1000? 4-00010200 0111-00100 0??{01}110100 000100?01? 0020???0?1 0002000?0? ?11???{12}{12}01 10?1000100 1220{01}02211 001(01)1(01)220? 1100211011 0000000000 00
		Mirza                100102?120 2211101000 20-0221000 1022--00-0 00100-2-01 000101111- -335120111 22----1110 0010110001 0132331-11 1111222210 1121133112 1121011200 010?100010 0022202001 0010201101 0010000120 0011100-0- 0-02010000 020(01)121101 201111022{01} {01}331110112 ??0??????? ???0010200 0111-00??0 0???110100 ?00100???? 00???0?0?1 ?????0??0? ?1????2101 ?0??00{01}?0? ???0?02211 001120???? ?????1???? ??000????? ??
		Allocebus            100102?120 2211101000 20-0221000 1(01)22--10-0 00100-2-01 100001111- -315010111 10----1110 1000110000 0132330-21 1000221210 1111112012 1121010001 010??00010 0012302001 0010201101 0010000220 00111(01)000- 0-020200?0 0201121001 2{01}11010001 1331110102 ??0??????? ???0010200 0111-01??0 0???110100 ?00100???? 00???0?0?1 ?????0??0? ?1????1100 20??001?0? ???0?02211 001011???? ?????1???? ??000????? ??
		Altanius             1??1?????? 0?0??????? 0????00011 1121201?1? 0110201001 01?2111100 1211100111 1022220000 1000013301 0132330?12 1311221200 1122100101 0101?????? ?????????? ?122?1?000 00??201111 0010000020 1022-00010 (01)100-21211 0201021101 1011111221 1220111002 ?????????? ?????????? ?????????? ????????0? ?????????? ?????????? ?????????? ?????????? ?????????? ?????????? ?????????? ?????????? ?????????? ??
		Absarokius           100102???? ??0????00? 0??0001001 1222--1300 011(01)102101 100220(12)000 -000500111 1(01)22220010 1(01)10012200 0132220211 (01)001211200 1122220001 0002?????? ?????????? ?022033100 00102(01)1011 0000000020 1022-00010 0?00-210(01)(12) 110(01)10010? 2011101121 1310121001 ?????????? ?????????? ?????????? ????????0? ?????????? ?????????? ?????????? ?????????? ????????02 0111111111 ??1110110? ?????????? ?????????? ??
		Anaptomorphus        100102???? 0?0????10? 1?????11?1 11?2101?0? 011?102101 10?2111000 0?{01}1300111 1122211010 1010012200 0132220211 (01)302211200 11(01)2(12)22001 0102?????? ?????????? ???2???10? 00?02000?? ?0???00020 1022-00010 0?00-100(01)2 110111010? 2001010111 122111{01}001 ?????????? ?????????? ?????????? ????????0? ?????????? ?????????? ?????????? ?????????? ?????????? ?????????? ?????????? ?????????? ?????????? ??
		Anemorhysis          11?124???? 2?0????20? ?????01001 1121101?0? 0110201101 10?1012100 011(12)100111 1122220000 (12)010012200 0132330?21 (01)300221200 112211(12)101 0102?????? ?????????? ??22?1?100 00??211111 0010000020 1022-00010 0?00-21211 0101111101 2000011111 1321111002 ?????????? ?????????? ?????????? ????????0? ?????????? ?????????? ?????????? ?????????? ?????????? ?????????? ?????????? ?????????? ?????????? ??
		Arapaphovius         110124?020 220110220? 2010001001 1120201300 1211201101 1001102111 112(23)(01)00111 1122220010 2210012200 0132220(12)21 1311222200 1022211(01)01 0102????02 ?1??{12}?0?1? ?022002110 001020101? 0010000020 1022-00020 0?01121111 0(12)01121101 1001111221 1221111001 ?????????? ?????????? ?????????? ????????0? ?????????? ?????????? ?????????? ?????????? ?????????? ?????11111 ??0120210? 201???1?0? ?????????? ??
		Dyseolemur           100103??10 0?03?0?00? 000???1001 11?2201?00 0110101101 01?2111111 1?21100111 1111110120 1(12)10012200 0132220211 1000222000 1(12)22200000 000210?100 020?100110 2022203100 00102002?? 0010000(01)20 1022000020 0?0112{01}110 ?2011100?? 2001101121 1?21110001 ?????????? ?????????? ?????????? ????????0? ?????????? ?????????? ?????????? ?????????? ?????????? ?????????? ?????????? ?????????? ?????????? ??
		Hemiacodon           110113?0?? 2{12}0?1?{12}10? 2??{01}001001 1110202310 02(01)2201001 0100011111 2233000111 2122120{01}0(01) (12)210012200 0131220111 1{01}11222100 0122(12)12101 0102??1??? ???????010 0022{01}0?100 00??2000?1 0(01)10000120 102{12}000020 0?(01)0-21221 (01)20(12)0(12)1101 2011101111 132022{01}000 ?????????? ?????????? ?????????? ????????0? ?????????? ??103?1011 01110????? ??????0200 2111101101 0110111111 001110110? 2011??1?0? ?????????? ??
		Loveina              10?102???? {01}?0????0?? ??????1001 1111001?1? 0110001101 01?2112111 1132200111 2122210010 1010012200 01322(23)0(12)21 1000222100 11(12)2202000 0102?????? ?????????? ???2?1?10? ?1??20?1?0 0?10?00020 1022-00010 1100-20{01}11 110102011? 2011101011 1331110001 ?????????? ?????????? ?????????? ????????0? ?????????? ?????????? ?????????? ?????????? ?????????? ?????????? ?????????? ?????????? ?????????? ??
		Macrotarsius         1??103???? 1?0????1?? ??????1001 10?1101?1? ?21?200001 01?2110121 1??(12)000111 1222220010 1120(01)12200 0131110121 1001222200 1112201002 0002?????? ????????1? ??22?1?10? 01??1011?0 0?10000220 1022-00020 21?0-21011 1101022211 2002200221 112122{01}000 ?????????? ?????????? ?????????? ????????0? ?????????? ?????????? ?????????? ?????????? ?????????? ?????????? ?????????? ?????????? ?????????? ??
		Microchoerus         110124??20 1101?0220? 2010000000 1222100?1? 0211101001 11?2112111 0231000111 112---1111 222101030{01} 0132320211 1302122000 1122210100 0112111220 021?010030 1022012210 0010201000 0011000220 2000100130 (01)100-2{01}111 0101111100 2001110111 133011(01)001 ?????????? ???????1?1 ??11-01?00 11???1000? ???10?11?? ??103?1011 0110000??? ?????????0 ??11201??? ?????11201 1002201??? ?????????? 0????????? ??
		Nannopithex          110124??20 1201102?0? 0010?01100 122210121? 0110001101 01?2212001 0002200111 1121110101 1(01){01}0012200 0132330?11 1302222200 1122212101 (01)00211122? ?20??1?030 1122011100 0010201000 0000000(01)10 1022-00010 11011(01)1{01}(01)0 11010(12)1100 2001001121 1321111002 ?????????? ?????????? ?????????? ????????0? ??020????? ?????????? ?????????? ????????02 ?011????01 0????????? ?????????? ?????????? 00???????? ??
		Necrolemur           110124??20 1201?0220? 0010001000 1222101?1? 0211101101 11?2112111 0231000111 112---1101 1120010300 0132330211 1302222000 1122211100 021211122? ?2??0???3? ?022012(12)10 0010201000 00100002(12)0 201110(12)(01)20 0?00-2{01}001 1101111100 2000010111 1330111001 0?10000010 1110?22111 2011-01100 1110010001 1001001111 1????????? ?????????? ????002112 2011011?1? 0111011111 0001101?1? ??????1?0? 00???????? ??
		Omomys               11012(34)?0{12}0 11010?210? 0001011001 1122101300 0110101001 0100011111 023(34)200111 1121110010 101001{23}200 0132(23)(23)0(12)21 {01}000222100 1112112101 01011???0? ???????11? ?022012100 0010201011 00(01)0000120 1022100010 0?(01)1121210 0201021101 2011000111 0111111000 0?10000000 011??????? ?021?????0 ??????0?0? ??????11?? ?????????? ?????????? ????000200 ?0112???01 011?011111 001110110? 2????????? 00???????? ??
		Pseudoloris          11?124?0?? 2????0?20? 0?1?001000 122210(01)?0? 0110101101 00?1112101 001(34)100111 111---0000 1010(01)10100 0132330?21 1{03}00222100 1021112101 {01}21111122? 1?????0030 1122112200 0010201001 0010000120 1011100010 0?00-21210 0201022101 1010000120 0111110001 ?????????? ?????????? ?????????? ????????0? ??020????? ?????????? ?????????? ?????????? ?????????? ?????????? ?????????? ?????????? ?????????? ??
		Shoshonius           10?102???? 0?0????00? 0011001001 1111201310 1110(01)00101 0002112111 11(23)(12)200111 1122210020 (12)(12)(12)0012200 0132220121 1300222100 1112202001 0002?????? ?????????? ?022011100 (01)11?200111 0110000(01)?0 1022-00020 1110-20010 1201021111 2001100221 1221110001 0?000200?0 0110?22111 0021?01??? ?110011?01 1?010011?0 1?10??0011 11110?010? 00??000200 211110??02 0111011111 000110210? ??1?????0? 00???????? ??
		Steinius             11?103???? {12}?0?????1? ??????0001 1022101?0? 01(01)2201101 00?0012111 1233100111 2122220010 1010012200 0132(23)(23)1(12)11 0101222100 1022112101 0102?????? ????????{01}? ??22?1?100 00?020101? 0010000110 1022-00010 0?011210(01)0 0101011111 2011100111 1221111001 ?????????? ?????????? ?????????? ????????0? ?????????? ?????????? ?????????? ?????????? ?????????? ?????????? ?????????? ?????????? ?????????? ??
		Strigorhysis         10?112???? 0?0????10? ??????1101 12?2??1?0? 011?(12)00101 1102111001 ?001300111 11?2210000 2210012200 0132?2?211 (01)00?222000 12(01)2(12)(12)0001 0002??1??? ?????????? ?02202?100 001020001? 0000000020 1022-00010 0?00-100(01)2 1101(12)11100 2001010221 1221221001 ?????????? ?????????? ?????????? ?????????? ?????????? ?????????? ?????????? ?????????? ?????????? ?????????? ?????????? ?????????? ?????????? ??
		'Teilhardina a.'     110113???? 2?0????{01}0? 000100(01)001 1021201300 0111(12)01101 10001(01)1100 01(12)2100111 1022210010 1000012200 0132(23)(23)0221 (01)100222200 11(12)2212(01)01 0102?????? ?????????? ??22?(01)?100 00?0201110 0?10000020 1022-00010 0?0{01}1212(01)1 110(01)011101 2011100221 1221111001 ?????????? ?????????? ?????????? ????????0? ?????????? ?????????? ?????????? ?????????? ?????????? ?????????? ??????200? ?????????? ?????????? ??
		'Teilhardina b.'     11?10{23}???? ??0????00? ????00(01)001 1021101?0? 0111201101 00?0011000 11(12)(34)300111 1022210010 1000012200 0132220221 (01)100222200 1112212(01)01 0102?????? ?????????? ??22?{01}2100 00??201110 0000000020 1022-00010 0?0{01}1112(01)1 010(01)11{01}101 1010000111 1321111002 ?????????? ?????????? ?????????? ????????0? ?????????? ?????????? ?????????? ?????????? ?????????? ?????11111 0011202??? ?????????? ?????????? ??
		Tetonius             110124?020 220110220? 0011011001 1221101300 0111102101 1202111001 1011(23)00111 1022210000 1000013200 0132220211 (01)(012)00222201 1(12)(01)2(12)20001 0102{12}0122{12} ?1??20?1?0 1022023100 00102(01)0111 (01)(01)(01)0000010 1022-00010 0?010212(01)1 110(01)011101 2001010111 1220111002 0??00????0 0110?2211? ?011?01??0 11100?1?0? ????00111? ?????????? ?????????? ?????????? ?????????? ?????11111 ??1???1??? ?????????? ?????????? ??
		Uintanius            10?10(23)???? 1?0????00? ??????1001 1221001?0? 0010002101 10?2201001 2110500111 1111110010 1000012200 0132220221 1000222200 11{12}2112001 0002?????? ?????????? ??22?2?001 00??200010 0000000120 1022-00020 0?00-(01)0010 ?101(01)(12)1200 2011011011 1331110001 ?????????? ?????????? ?????????? ?????????? ?????????? ?????????? ?????????? ?????????? ?????????? ?????????? ?????????? ?????????? ?????????? ??
		Washakius            100102?0{01}? 0?0110200? 00?{01}001001 1111200(23)10 11(01)0001101 0002112111 212(12)200111 1121110{01}(12)1 22(12)0012200 0132110121 1201122000 1112202000 0002?????? ????????10 2022011100 (01)000201110 0011100(01)20 2011000020 1110-20120 010(12)021111 2001111001 1330110000 ?????????? ?????????? ?????????? ????????0? ?????????? ?0???????? ?????????? ?????????? ?????????? ?????11111 ??0???2??? ?????????? ?????????? ??
		'Tarsius s.'         2??1???120 0001?00010 0102001000 1222--1210 011{01}{01}01101 0021101111 -00(12)(01)00111 1(01)11110010 1000013200 01(23)2{23}{23}1120 0000211200 1{01}21111001 1101200200 120?201111 1022003101 00102(01)1022 0010000120 10{12}21000{01}0 0?020(12){01}{01}10 0201(01)2010? 2011100220 0111110100 1001121211 21111?1111 2022101210 1210011101 1111021000 1100302011 0002000000 0000000200 221120111? 0111011001 00(01)0001110 2011211011 1111111111 11
		Xanthorhysis         ?????????? ????????1? 0????01001 1221101?1? 0111101101 00?1101111 0103100111 1111110000 1000012200 0132330?20 0000222200 1122122001 0001?????? ?????????? ?????????? ?????????? ?????????? ?????????? ?????????? ?????????? ?????????? ?????????? ?????????? ?????????? ?????????? ?????????? ????0????? ?????????? ?????????? ?????????? ?????????? ?????????? ?????????? ?????????? ?????????? ??
		'Afrotarsius c.'     ?????????? ?????????? ?????????? ?????????? ?????????? ????1????? ???0?20111 ?011100000 1000002200 0132222110 0000222210 1022121101 0101?????? ?????????? ?????????? ?????????? ?????????? ????-????? ?????????? ?????????? ?????????? ?????????? ?????????? ?????????? ?????????? ?????????? ?????????? ?????????? ?????????? ?????????? ????????1? ?????????? ?????????? ?????????? ?????????? ??
		Afrasia              ?????????? ?????????? ?????????? ?????????? ?????????? ?????????? ??????0?11 21?110?100 1000012?0? 0120?31-1? 0?00222210 102?02?102 0202?????? ?????????? ?????????? ?????????? ?????0?120 1022-0?010 0-02(01)2(01)(02)?1 0??11?2211 1011100220 0111110{01}{01}0 ?????????? ?????????? ?????????? ?????????? ?????????? ?????????? ?????????? ?????????? ?????????? ?????????? ?????????? ?????????? ?????????? ??
		Eosimias             1001000000 000010002? 0002001001 1121201310 0110{01}0110(01) 1011111100 1203220111 10(12)1100(01)00 1000013300 01(12)2(12)(12)1{12}21 1000222100 1011113101 0102?????? ?????????? ??22???000 00???01121 101000?{01}?0 0022-0000- 0?02020010 01011?1111 2011001220 0111110110 ???????110 0????????? ?????????? ????????0? ??011????? ?11?2??011 1??0?00??? 1????????? ????????00 01???11111 01111001?? ????????2? ?????????? ??
		Bahinia              1?0?000000 000010102? 0??2001001 10?2??22?0 001110?101 0100011112 ?{12}332101?? ??1???1?1? ?00011?300 ?1123???11 ?0??22?200 1001??2101 0???1?010? ?0?0???011 102201301(01) 0010201?01 ?01000?120 0022-0??0- 0?020200?0 0??01?1111 2011100220 0111110110 ?????????? ?????????? ??0??1???0 ??????1?0? ????1???1? ?1???????? ?????????? ?????????? ?????????? ?????????? ?????????? ?????????? ?????????? ??
		Phenacopithecus      ???????000 0000100??? ???????001 1?22??1310 0110{01}01101 012111?100 -?332?0111 101110(01)110 1000012300 0122(12)20111 1111222100 1011113101 0202?????? ?????????? ???2?0?00? 00??2011?1 1?10?0?120 1022000000 0?03020010 01011?2111 2021100220 01111101(01)0 ?????????? ?????????? ??0??????? ?????????? ?????????? ?????????? ?????????? ?????????? ?????????? ?????????? ?????????? ?????????? ?????????? ??
		Phileosimias         ?????????? ?????????? ?????????1 1?21201?1? 0110101101 01?111?102 0??31?0111 2111100010 1000012300 0131110110 0000221(12)10 11{12}11?2101 0102?????? ?????????? ?????????? ?????????? ?????00120 1022-00020 0?0(12)010011 0101111101 2011100220 0111110000 ?????????? ?????????? ?????????? ?????????? ?????????? ?????????? ?????????? ?????????? ?????????? ?????????? ?????????? ?????????? ?????????? ??
		Proteopithecus       1????????? ?????????2 ???2001001 1221101010 0210201001 1121120100 1112210111 1011-10010 (01)010011200 013112(123)011 0000222100 1111130102 0001???1?? ?0?????011 1022020100 00002011?0 1011001110 1011100?{01}0 0?02020000 1000100100 2001100220 0111110?00 1111001210 ?1110?00?0 0?02011??1 1221111000 11020???01 01110?1121 1110101?0? 11??002210 10?0?10??? ?????10000 000221???? ?????????? 11???????? ??
		Biretia              ?????????? ?????????? ???????001 1022101?10 0110111101 0020?10100 0233?20111 1011-10000 1010(01)0{12}200 0?30110(01)11 0000211210 10111(23)2101 0101?????? ?????????? ?222111100 0000201100 0001100120 1000100020 0?02010010 1101110100 200110100? 0?111101(01)0 ?????????? ?????????? ?????????? ?????????? ?????????? ?????????? ?????????? ?????????? ?????????? ?????????? ?????????? ?????????? ?????????? ??
		Serapia              2????????? ????????1? 1002001001 1121101010 0110101001 1011010000 0200120111 1011-10{01}00 1011011200 0130000001 0000221110 1111130000 0002?????? ?????????? ?????????? ?????????? ?????????? ?????????? ?????????? ?????????? ?????????? ?????????? ?????????? ?????????? ?????????? ????????0? ?????????? ?1???????? ?????????? ?????????? ?????????? ?????????? ?????????? ?????????? ?????????? ??
		Simonsius            3????????? ??0?????1? 0002001001 1011?00100 1110011011 0001020011 ?230220111 00----1110 1021110011 1030002110 0000211110 1111120001 0102?????? ????????1? ?122011100 0000010000 0000000210 1000100120 0?020(12)0011 1001100100 200110?00? ???1110010 1111001??? 21110?0110 ??02011??1 1201101010 11?102??01 01???????? ?????????? 1???1????? ?????????? ?????????? 000??????? ?????????? 11???????? ??
		Qatrania             2????????? ?????????? ???????001 10?2101?0? ?11?01?111 10?0120000 0??(01)020111 001---0100 1011010200 0130001101 00002{01}1120 1212101001 0002?????? ?????????? ?????????? ?????????? ?????0?1?? 100?10?020 0?02?000?1 ???10????? 200??000?0 ???02?0??0 ?????????? ?????????? ?????????? ?????????? ?????????? ?????????? ?????????? ?????????? ?????????? ?????????? ?????????? ?????????? ?????????? ??
		Apidium              1001010000 0000102012 0002001001 1022--0000 1110011110 1012010000 -131120111 111---1110 2021110111 1030001101 0000011101 1212120000 0002?????? ?0??????1? ?122111100 0000000{01}00 0000000120 1200010130 0?23010021 0022111100 200110?00? ???1110010 1111?0?210 21110????? ?1020?1??1 12????101? ???10110?? ?0110(01)(12)021 1110101000 10??1?2112 2000010002 0110111010 00(01)2210101 0021?????0 11???????? ??
		Parapithecus         ?00101{01}000 000010201? 0002001001 1022--0100 1110011110 1012020000 -222320110 00----1111 1021110011 1030001101 1100211120 1212120000 0102?????? ????????1? ?????????? ?????????? ?????????? ?????????0 ?????????? ?????????? ?????????? ?????????? ?????????? ?????????? ?????????? ????????1? ?????????? ?1???????? ?????????? ?????????? ?????????? ?????????? ?????????? ?????????? ?????????? ??
		Arsinoea             10011{01}?010 000010201? 10020010?? 1222101200 1111101101 1001111100 1210120111 1022-10010 1011113300 0121111100 0001211200 1201131001 0102?????? ?????????? ?????????? ?????????? ?????????? ?????????? ?????????? ?????????? ?????????? ?????????? ?????????? ?????????? ?????????? ????????0? ?????????? ?0???????? ?????????? ?????????? ?????????? ?????????? ?????????? ?????????? ?????????? ??
		Oligopithecus        ?????????? ?????????? 00020011?1 1021102?1? 0210201001 01?1020011 1233110111 1121-10010 1020011200 0131110010 0000211100 1001131101 000(12)?????? ?????????? ?02201?100 0110200010 1000000120 0011100?0- 0?03000001 010011(01)100 2021100220 0111110210 ?????????? ?????????? ?????????? ?????????? ?????????? ?????????? ?????????? ?????????? ?????????? ?????????? ?????????? ?????????? ?????????? ??
		Catopithecus         1?0??10010 000010??12 00020011?1 1011100?1? 0210101001 11?0020011 1221110111 1111-10110 101000{12}200 0131111010 0000211200 1011030101 0102100001 1000200031 002201?100 0010201000 1010000120 0011100?0- 1103020001 0100(01)11101 2011000220 0111110110 ??1?001210 ???10????0 01020?1??1 12?1??1000 11020???11 00101?0001 1110101??? 12??011210 ?000000??? ?????11010 110211???? ?????????? 11???????? ??
		Aegyptopithecus      1000010010 0000101012 00020011?1 1022--1?1? 0200101001 00?1030101 -330120111 11----1110 102110000(01) (01)030000100 0001211220 1211010002 0002?????? ???????031 102201?100 0110211000 1002200120 1100100010 0?03010001 200{01}12010? 2001000100 0111110110 101?011210 21110?00?0 0102011??1 1221110010 1112121011 011010(01)001 1110101000 12??01?0?(12) 0?0??0??00 ?????11000 1112110101 ??21??0?20 11???????? ??
		Moeripithecus        ?????????? ?????????? ??????11?1 1022--1?1? 0201101001 11?1?20011 -331220111 111---1110 1021100100 0020000101 0101211200 1111020001 010{12}?????? ?????????? ?01201?100 01?0201000 100000?120 000111??0- 0?130100?(01) 2??02?010? 2001100210 0111110110 ?????????? ?????????? ?????????? ?????????? ????1????? ?????????? ?????????? ?????????? ?????????? ?????????? ?????????? ?????????? ?????????? ??
		Siamopithecus        ?????????? ????????1? 0002001001 1022--1?0? 0110100001 01?1111001 -23(12)210111 10----?100 1121100000 0122221200 0112221200 1200000002 0102?????? ????????1? ??2201?10? 00??2(01)0000 0000001010 1122---032 0?00-00202 2?001000?? 2001110221 11111102(12)0 ?????????? ?????????? ??0??????? ??????1?0? ????1????? ?1???????? ?????????? ?????????? ?????????? ?????????? ?????????? ?????????? ?????????? ??
		Pondaungia           1?0103?0?? 0?0????02? 0??2001001 102110(12)210 1100(12)01000 0101121010 12(23)(01)020111 1(01)11-11110 1211113201 1032{23}{23}0{12}00 01{12}22112{01}0 1(12)100(012)0002 01021???22 ?000100010 1022013100 00??1010?0 0000000120 1(01)22---(01)(23)(12) (01)10(12)(01)101?1 21?001010? 2001110111 1111220220 ?????????? ?????????? ?????????? ??????110? ???11????? ?1???????? ?????????? ?????????? ?????????? ?????11010 001211???? ?????????? ?????????? ??
		Ganlea               1??101?010 000?00?02? 00?3001001 1101001?0? 0100212100 01?1111010 223002011? 1?----112? ?211110000 013233??00 011?21?210 11110?0112 0102?????? ?????????? ?????????? ?????????0 ?????0?2?0 10?{12}10?021 0?010100?1 0???0?00-- 2001110?1? 1?11?20210 ?????????? ?????????? ?????????? ????????0? ????1????? ?1???????? ?????????? ?????????? ?????????? ?????????? ?????????? ?????????? ?????????? ??
		Myanmarpithecus      ?????????? ????????1? 0??2001001 ?2?????2?0 0??1??1?01 ??01??{12}??? ??????0111 12?---?110 1210110?00 0132331?00 0012211210 11(01)1100112 0102?????? ?????????? ???2?0?10? 01??1110?0 0000000220 1011100021 1102010001 0201011100 2001110111 1111220210 ?????????? ?????????? ?????????? ????????0? ????1????? ?????????? ?????????? ?????????? ?????????? ?????????? ?????????? ?????????? ?????????? ??
		Bugtipithecus        ?????????? ?????????? ?????????? 1??2--0?1? ?11?10?001 00??01?001 -??3??0111 ?2-?--1120 101011?000 ??323?1?10 ?0?0211100 1111??1112 0????????? ?????????? ???2???10? 00??2010?0 0?00?0?2?0 100012-010 0?020100?0 0??00?1100 20{01}1100220 0111220120 ?????????? ?????????? ?????????? ?????????? ?????????? ?????????? ?????????? ?????????? ?????????? ?????????? ?????????? ?????????? ?????????? ??
		Branisella           110111?1?0 000?00?01? 00??001000 0022--0210 1211200001 1101010001 -220020111 11----1110 0011110000 0132222100 0110221200 0101130101 0002?????? ?????????? ?01100?100 01??201100 1000001110 000000000- 0?{01}3000000 0000201200 2?01100100 0131110100 ?????????? ?????????? ?????????? ????????1? ?????????? ?1???????? ?????????? ?????????? ?????????? ?????????? ?????????? ?????????? ?????????? ??
		Dolichocebus         1?????0000 ?000101?12 0002??1000 0122??1201 1211201101 0002000002 ??301?011? 1?1-?1112? ?020110100 013022?111 012?22?100 11110?0101 01021000?1 0000100011 1111?00100 00?02011?0 1001101120 000011000- 0?13010000 1111101100 2101100110 0331110200 ?0???????? ?????????? ??02?????? ????????1? ??????1??? ?????????? ?????????? ?????????? ?????????? ?????11010 00?211???? ?????????? 11???????? ??
		Neosaimiri           1000111000 000?10?0?? 000?001000 0022--1211 2201200001 0001010012 -2302?0110 10----112? 0010110000 0131(12)23110 02222212{01}0 1111132101 0112???111 ?000100110 1111210100 0101201100 1001002110 000011?00- 0?13000000 {01}000201100 2101100210 0111110200 ?????????? ?????????? ?????????? ????????1? ?????????? ?????????? ?????????? ?????????? ?????????? ?????????? ?????????? ?????????? ?????????? ??
		Saimiri              1001210000 000010?022 0002001000 0022--0210 2202200001 0001010001 -230(23)-0110 (01)0----111? 0020110000 0132{23}{23}3111 0001222200 1112140101 0112100221 ?000200111 21111(12)0100 0111210000 1000012110 000011??0- 0?130(01)0000 {01}110101100 2101100110 0111110210 1111011210 21110?00?0 (02)1020?1211 1211111010 0112111001 01102(01)(23)001 (12)110102000 1200(01)01(01)12 1000002001 0110011010 00111021?1 0021100120 1111111111 11
		Callicebus           1001011110 000010?010 0002001000 0022--0210 2201200001 ?100020121 -2300-0110 11----112? 0021110000 0131113110 0222221110 0001030101 0102100111 0000210110 2111100100 0000210000 1001101(12)10 0000122?0- 0?02000000 001010010? 2002200110 0331110210 1111011210 21110?00?0 01020?1211 1201121010 110211100? ?110(12)(01)22?1 1110102000 ??00??2012 1000002001 0110011010 ??1210110? 0021100120 1111111111 11
		Aotus                1001120000 000010?010 0002001000 0022--1211 2201100000 0100020011 -2(23)0(12)-0110 11----111? 0020100000 0132333?10 02222{12}{01}200 1001130101 0102100222 0000200110 11112(01)0100 0100210000 00000122{12}0 0000122?0- 0?01000000 11102100?? 2001100110 0111220220 1111011110 21110?00?0 01120?1211 1211111010 1112111011 01(01)01(01)(123)101 (01)110102000 1?00(01)02012 1000102002 0110011010 00121011?1 0021100120 1111111111 11
	;
ENDBLOCK;


BEGIN ASSUMPTIONS;

	OPTIONS  DEFTYPE=unord PolyTcount=MINSTEPS ;
	
	TYPESET * default = ORD: 1 5 - 7 9 11 - 12 17 - 20 25 - 26 32 - 35 37 41 - 42 52 - 57 59 - 66 71 - 76 79 81 - 83 87 - 88 93 - 101 106 - 108 112 - 117 120 122 - 125 128 - 131 135 139 141 - 146 155 159 - 160 164 - 177 179 - 181 183 - 184 186 - 198 201 - 205 208 - 209 217 - 220 223 226 228 243 - 244 253 256 264 273 275 277 282 - 285 287 292 297 - 298 300 - 302 305 307 310 312 - 313 317 - 318 324 - 328 331 333 339;

	WTSET * saved  = 1: 1-352;

ENDBLOCK;


BEGIN PAUP;

	[DELETE xxx xxxx/only;]
	
    OUTGROUP Scandentia Plesiadapis Plesiolestes Ignacius Paromomys Purgatorius/only;
    
    ASSUME ANCSTATES= STANDARD;

	SET/INCREASE= AUTO;

	[loadconstr asbackbone=yes;]
	   
ENDBLOCK;


log file = Hsearch.out;

HSEARCH
NREPS = 1000
ADDSEQ = RANDOM;
[enforce=yes;]
