# Supplementary material for: Djebelemur, a Tiny Pre-Tooth-Combed Primate from the Eocene of Tunisia: A Glimpse into the Origin of Crown Strepsirhines
Source: PLoS One. 2013 Dec 4;8(12):e80778. doi: 10.1371/journal.pone.0080778 (PMC3851781; doi:10.1371/journal.pone.0080778)
Supplement: Dataset S4 — Molecular-Scaffold: gene-based tree of modern taxa deriving from Perelman et al. (2011) [28] . (RTF) [file pone.0080778.s004.rtf]

Dataset S4


Molecular-Scaffold: gene-based tree of modern taxa deriving from Perelman et al. (2011).

Perelman P, Johnson WE, Roos C, Seuanez HN, Horvath JE, et al. (2011) A molecular phylogeny of living Primates. Plos Genetics 7: e1001342.


#NEXUS

[File saved Fri Apr 06 18:22:30 2012]

BEGIN TAXA;
	DIMENSIONS NTAX = 20;
	TAXLABELS
		Scandentia
		Varecia
		Eulemur
		Lemur
		Hapalemur
		Lepilemur
		Cheirogaleus
		Microcebus
		Mirza
		Propithecus
		Avahi
		Loris
		Nycticebus
		Perodicticus
		Arctocebus
		Galago_s.
		Otolemur
		Tarsius_s.
		Saimiri
		Aotus
		;
ENDBLOCK;


BEGIN TREES;
	TRANSLATE
		1	Scandentia,
		2	Varecia,
		3	Eulemur,
		4	Lemur,
		5	Hapalemur,
		6	Lepilemur,
		7	Cheirogaleus,
		8	Microcebus,
		9	Mirza,
		10	Propithecus,
		11	Avahi,
		12	Loris,
		13	Nycticebus,
		14	Perodicticus,
		15	Arctocebus,
		16	Galago_s.,
		17	Otolemur,
		18	Tarsius_s.,
		19	Saimiri,
		20	Aotus
		;
	UTREE * PHYLIP_1= (1,((((2,(3,(4,5))),(6,(7,(8,9))),(10,11)),(((12,13),(14,15)),(16,17))),(18,(19,20))));
ENDBLOCK;
